# Supplementary material for: Observation of mechanical kink control and generation via acoustic waves
Source: Nat Commun. 2026 Feb 6;17:2428. doi: 10.1038/s41467-026-68688-7 (PMC12988211; doi:10.1038/s41467-026-68688-7)
Supplement: Supplementary file 1 — Supplementary Information [file 41467_2026_68688_MOESM1_ESM.pdf]

Observation of mechanical kink control and generation via acoustic waves

Kai Qian[1,2], Nan Cheng[3+], Francesco Serafin[3,4+], Nicolas Herard[1], Kai Sun[3], Georgios Theocharis[5*], Xiaoming Mao[3*], and Nicholas Boechler[1,6*]

[1]Department of Mechanical and Aerospace Engineering, University of California San Diego, La Jolla, CA 92093, USA
[2]George W. Woodruff School of Mechanical Engineering, Georgia Institute of Technology, Atlanta, GA 30332, USA
[3]Department of Physics, University of Michigan, Ann Arbor, MI 48109, USA
[4]Department of Physics and Materials Science, University of Luxembourg, 2 Avenue de l'Universite L, 4365 Esch-sur-Alzette, Luxembourg
[5]Laboratoire d'Acoustique de l'Université du Mans (LAUM), UMR 6613, Institut d'Acoustique - Graduate School (IA-GS), CNRS, Le Mans, France
[6]Program in Materials Science and Engineering, University of California San Diego, La Jolla, CA 92093, USA
[*]these authors are co-corresponding authors: georgios.theocharis@univ-lemans.fr; maox@umich.edu; nboechler@ucsd.edu
[+]these authors contributed equally to this work

Supplementary Note 1. THEORY ON THE KANE-LUBENSKY (KL) CHAIN

1.1 Geometric phase diagram of the zero-energy kink with unique unit cell geometry

We introduce a geometric phase diagram of the zero-energy kink in the KL chain parameterized by two dimensionless geometric variables, $\tilde{\bar{l}} \equiv \bar{l}/a$ and $\tilde{r} \equiv r/a$, and overlay the widths of the F- and WF-phase kinks, as shown in Supplementary Fig. 1.

To construct this geometric phase diagram, we begin with the instantaneous spring length between rotors n and $n+1$ in the KL chain

$$l_{n,n+1}(\psi_n, \psi_{n+1}) = \sqrt{(a - r\sin\psi_n + r\sin\psi_{n+1})^2 + (r\cos\psi_n + r\cos\psi_{n+1})^2}, \tag{1}$$

which yields the normalized unstretched spring length

$$\tilde{\bar{l}} \equiv \frac{\bar{l}}{a} = \frac{l_{n,n+1}(\bar{\psi}, \bar{\psi})}{a} = \sqrt{1 + 4\tilde{r}^2\cos^2\bar{\psi}}. \tag{2}$$

By considering $\bar{\psi} \in [0, \pi/2]$, Supplementary Eq. 2 indicates two boundary condition functions of $\tilde{\bar{l}}$ in the phase diagram at $\bar{\psi} = 0$ and $\pi/2$, which correspond to

$$\tilde{\bar{l}} = \sqrt{1 + 4\tilde{r}^2} \tag{3}$$

and

$$\tilde{\bar{l}} = 1, \tag{4}$$

respectively. Supplementary Equations 3 and 4 define the upper and lower boundaries of the available zero-energy kink states in Supplementary Fig. 1. Outside these boundaries, there is no kink state with unstretched springs, *i.e.*, no zero-energy kinks. Then, from $d = 2\tilde{r}\sin\bar{\psi}$[1], which separates WF and S phases, we have

$$\cos^2\bar{\psi} = 1 - \sin^2\bar{\psi} = 1 - \frac{d^2}{4\tilde{r}^2}. \tag{5}$$

By substituting Supplementary Eq. 5 into Supplementary Eq. 2, we have

$$\tilde{\bar{l}} = \sqrt{1 + 4\tilde{r}^2 - d^2}. \tag{6}$$

Since $d = 1$ indicates the boundary between F and WF phases[1], using Supplementary Eq. 6, we obtain the boundary function between F and WF phases

$$\tilde{\tilde{l}} = 2\tilde{r}, \tag{7}$$

which is denoted as a black dashed line in Supplementary Fig. 1. Last, since $d = 2/\sin\bar{\psi}$ separates WF and S phases[1], we can write

$$2\tilde{r}\sin\bar{\psi} = \frac{2}{\sin\bar{\psi}} \tag{8}$$

as its critical condition, where $\sin\bar{\psi}$ can be obtained using Supplementary Eq. 2 such that

$$\sin\bar{\psi} = \frac{\sqrt{1-\tilde{\tilde{l}}^2+4\tilde{r}^2}}{2\tilde{r}}. \tag{9}$$

From Eqs.8 and 9, we obtain the boundary function between WF and S phases

$$\tilde{\tilde{l}} = 2\tilde{r}-1, \tag{10}$$

which is denoted as a black solid line in Supplementary Fig. 1.

We note that Supplementary Eq. 6 yields

$$d = \sqrt{1+4\tilde{r}^2-\tilde{\tilde{l}}^2}, \tag{11}$$

which shows that multiple pairs of $(\tilde{r}, \tilde{\tilde{l}})$ can produce the same d, *i.e.*, this phase index alone is insufficient to determine the unit cell geometry in the KL chain, which consequently affects the kink's dynamic properties (discussed further in a later section). In contrast, in Supplementary Fig. 1, each point corresponds to a distinct unit cell geometry.

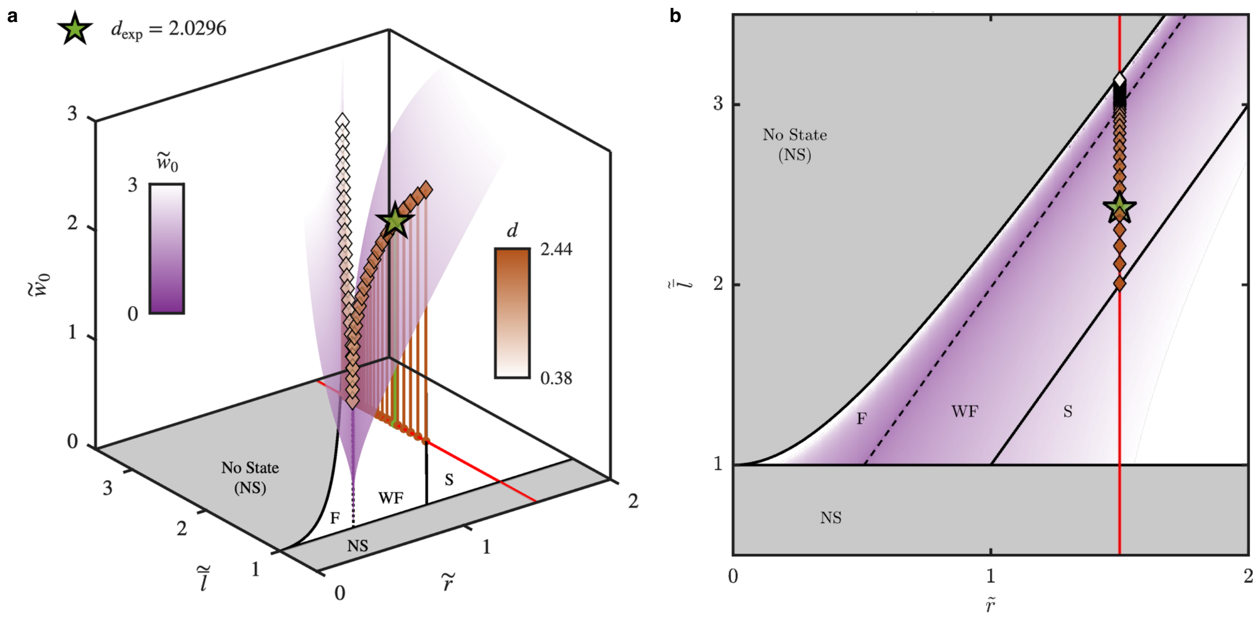

Supplementary Figure 1. Geometric phase diagram of the zero-energy kink, where the red line indicates $\tilde{r} = 1.5$, orange diamonds indicate kink states shown in Fig. 2**b** and **c**, and the green star denotes the kink state examined in experiments. **a** Three-dimensional (3D) view. **b** Top view.

1.2 Equations of motion

To avoid confusion, we clarify that two types of angular notation are used to describe the rotation of rotors in the KL chain: θ_n, defined as counterclockwise from the negative y direction, and ψ_n, where $\psi_n = \theta_n$ for the odd sites and $\psi_n = \pi - \theta_n$ for the even sites, with n as the rotor index, as shown in Supplementary Fig. 2.

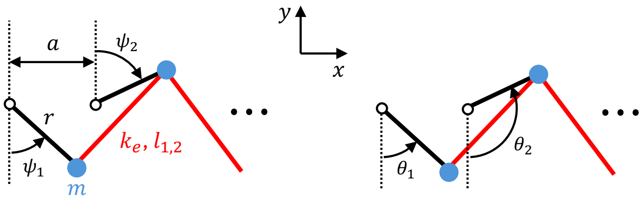

Supplementary Figure 2. Schematics for using ψ_n and θ_n notations in the KL chain.

Consider a 1D chain consisted of equally spaced harmonically coupled rotors with open boundary conditions and identical linear normal springs. The Lagrangian of such rotor chain is

$$L = \sum_n \left(\frac{1}{2} m r^2 \dot{\theta}_n^2 - \frac{1}{2} k_e (l_{n,n+1} - \bar{l})^2 \right), \tag{12}$$

where m is the mass at the end of the rotor, r is the radius of the massless rotor, k_e is the linear spring stiffness, and $\bar{l}$ is the unstretched (or rest) length of the spring. Using the Euler–Lagrange equation, from Supplementary Eq. 12, we have the equation of motion

$$mr^2\ddot{\theta}_n + k_e r \frac{(a\cos(\theta_n) - r\sin(\theta_{n-1} - \theta_n))\left(l_{n-1,n} - \bar{l}\right)}{l_{n-1,n}} - k_e r \frac{(a\cos(\theta_n) - r\sin(\theta_n - \theta_{n+1}))\left(l_{n,n+1} - \bar{l}\right)}{l_{n,n+1}} = 0. \tag{13}$$

From Supplementary Eq. 13, for such rotor chain with N rotors and open boundary conditions at both ends, the N equations of motion are

$$\begin{aligned}
\ddot{\theta}_1 &= \frac{k_e}{mr} \frac{(a\cos(\theta_1) - r\sin(\theta_1 - \theta_2))\left(l_{1,2} - \bar{l}\right)}{l_{1,2}}, \\
&\cdots \\
\ddot{\theta}_n &= -\frac{k_e}{mr} \frac{(a\cos(\theta_n) - r\sin(\theta_{n-1} - \theta_n))\left(l_{n-1,n} - \bar{l}\right)}{l_{n-1,n}} + \frac{k_e}{mr} \frac{(a\cos(\theta_n) - r\sin(\theta_n - \theta_{n+1}))\left(l_{n,n+1} - \bar{l}\right)}{l_{n,n+1}}, \\
&\cdots \\
\ddot{\theta}_N &= -\frac{k_e}{mr} \frac{(a\cos(\theta_N) - r\sin(\theta_{N-1} - \theta_N))\left(l_{N-1,N} - \bar{l}\right)}{l_{N-1,N}},
\end{aligned} \tag{14}$$

with $1 < n < N$.

In order to include onsite damping in simulations, we add an extra term $-c\dot{\theta}_n/(mr^2)$ to the right-hand side of each equation in Supplementary Eqs. 14, where c is the onsite damping coefficient. In order to apply the excitation, we add the term $\tau(t)/(mr^2)$ to the right hand side of the equation corresponding to the excited rotor in Supplementary Eqs. 14, where $\tau(t)$ is the driving torque.

1.3 Dispersion relation and acoustic wave group velocity

Consider a homogeneous KL chain with $\psi_n = \bar{\psi}$ and periodic boundary conditions. By applying an infinitesimal angular change $\delta\psi_n$ from the homogeneous configuration, we can expand the spring elongation $\delta l_{n,n+1}$ using a Taylor series such that

$$\delta l_{n,n+1} = l_{n,n+1}(\bar{\psi} - \delta\psi_n, \bar{\psi} - \delta\psi_{n+1}) \approx c_1 \delta\psi_n + c_2 \delta\psi_{n+1}, \tag{15}$$

where

$$c_1 = \frac{r\cos\bar{\psi}(2r\sin\bar{\psi} + a)}{\bar{l}} \tag{16}$$

and

$$c_2 = \frac{r\cos\bar{\psi}(2r\sin\bar{\psi} - a)}{\bar{l}}. \tag{17}$$

Note here c_1 and c_2 map the staggered hoppings in the Su-Schrieffer-Heeger (SSH) model[1,2]. By applying the Bloch's theorem, from Supplementary Eq. 15 we can have

$$\delta l_{n,n+1} = \left(c_1 + c_2 e^{ika}\right)\delta\psi_n = C(k)\delta\psi_n, \tag{18}$$

where k is the wavenumber. Supplementary Equation 18 leads to the homogeneous dispersion relation

$$\omega(k) = \pm\sqrt{\frac{k_e}{mr^2}}|C(k)| = \pm\sqrt{\frac{k_e}{mr^2}}\sqrt{c_1^2 + c_2^2 + 2c_1c_2\cos ka}. \tag{19}$$

By analytically rearranging Supplementary Eq. 19 analytically for k, we have the homogeneous inverse dispersion relation

$$k(\omega) = \frac{1}{a}\arccos\left(\frac{mr^2\omega^2 - k_e\left(c_1^2 + c_2^2\right)}{2k_e c_1 c_2}\right). \tag{20}$$

Analytically taking the derivative of Supplementary Eq. 19 with respect to k results in the homogeneous acoustic wave group velocity

$$v_g = \frac{d\omega_+(k)}{dk} = -\sqrt{\frac{k_e}{mr^2}}\frac{ac_1c_2\sin ka}{\sqrt{c_1^2 + c_2^2 + 2c_1c_2\cos ka}}, \tag{21}$$

where ω_+ indicates Supplementary Eq. 19 with a positive sign.

1.4 Non-unique kink's dynamical properties for a given phase index d

As noted earlier, d alone cannot uniquely determine kink's dynamic properties. To illustrate this, we show the dispersion relation of the homogeneous KL chain as a function of d.

We substitute Supplementary Eqs. 5 and 6 into Supplementary Eqs. 16 and 17, yielding

$$c_1 = \frac{r(d+1)\sqrt{1 - \frac{d^2}{4\tilde{r}^2}}}{\sqrt{1 - d^2 + 4\tilde{r}^2}} \tag{22}$$

and

$$c_2 = \frac{r(d-1)\sqrt{1 - \frac{d^2}{4\tilde{r}^2}}}{\sqrt{1 - d^2 + 4\tilde{r}^2}}. \tag{23}$$

Further substituting Supplementary Eqs. 22 and 23 into Supplementary Eq. 19, we have

$$\omega(k) = \pm\sqrt{\frac{k_e}{m}}\sqrt{\frac{(d^2 - 4\tilde{r}^2)(d^2 + 1 + (d^2 - 1)\cos ka)}{2\tilde{r}^2(d^2 - 4\tilde{r}^2 - 1)}}. \tag{24}$$

This result confirms that d alone does not fully determine the dispersion relation, where an additional parameter, such as $\tilde{r}$ (or $\tilde{\bar{l}}$), is required. Consequently, for a given d, variations in the unit cell geometry can lead to distinct dynamical behaviors of the KL chain and, in turn, its zero-energy kink.

Supplementary Note 2. THEORY ON THE DISCRETE ϕ^4 CHAIN

2.1 Equations of motion

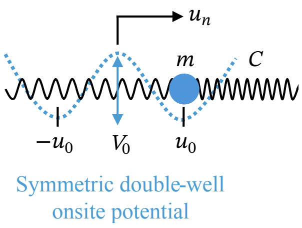

Supplementary Figure 3. Schematic of a discrete ϕ^4 chain section.

Consider the one-dimensional (1D) ferroelectric model introduced in Ref.[3] and shown in Supplementary Fig. 3, which consists of a chain of coupled particles, each in a double-well potential. The discrete Hamiltonian of such model reads

$$H = \sum_n \left(\frac{1}{2} m \dot{u}_n^2 + \frac{1}{2} C \left(u_{n+1} - u_n\right)^2 + V_0 \left(1 - \frac{u_n^2}{u_0^2}\right)^2 \right), \tag{25}$$

where m is the mass of the particle, C is the coupling constant, V_0 is the height of potential well, u_n is the displacement of the particle with respect to the midpoint of its two equilibrium positions, and u_0 is the local distance between the highest and lowest potential points. By introducing the momentum $p_n = m\dot{u}_n$ and using the Hamiltonian equation $dp_n/dt = -\partial H/\partial u_n$, from Supplementary Eq. 25, we obtain the discrete equation of motion

$$\ddot{u}_n - \frac{c_0^2}{a^2}\left(u_{n+1} - u_n\right) - \frac{c_0^2}{a^2}\left(u_{n-1} - u_n\right) - \omega_0^2 u_n \left(1 - \frac{u_n^2}{u_0^2}\right) = 0, \tag{26}$$

where a is the lattice spacing, $\omega_0 = \sqrt{4V_0/(m u_0^2)}$ and $c_0 = \sqrt{Ca^2/m}$. From Supplementary Eq. 26, for a finite discrete ϕ^4 chain with N particles and open boundary conditions at both ends, the N equations of motion are

$$\begin{aligned}
\ddot{u}_1 &= \frac{c_0^2}{a^2}\left(u_2 - u_1\right) + \omega_0^2 u_1 \left(1 - \frac{u_1^2}{u_0^2}\right), \\
&\dots \\
\ddot{u}_n &= \frac{c_0^2}{a^2}\left(u_{n+1} + u_{n-1} - 2u_n\right) + \omega_0^2 u_n \left(1 - \frac{u_n^2}{u_0^2}\right), \\
&\dots \\
\ddot{u}_N &= \frac{c_0^2}{a^2}\left(u_{N-1} - u_N\right) + \omega_0^2 u_N \left(1 - \frac{u_N^2}{u_0^2}\right),
\end{aligned} \tag{27}$$

with $1 < n < N$. In order to apply the excitation, we add the term $F(t)/m$ to the right hand side of the equation corresponding to the excited particle in Supplementary Eq. 27, where $F(t)$ is the driving force.

2.2 Dispersion relation and acoustic wave group velocity

Consider the discrete ϕ^4 chain in a homogeneous state with $u_n = u_0$ and periodic boundary conditions. By applying an infinitesimal displacement δu_n from the homogeneous configuration, assuming harmonic solutions, and employing the Bloch theorem, from Supplementary Eq. 26, we have

$$-\omega^2 \delta u_n + \frac{2c_0^2}{a^2}\left(1 - \cos ka\right)\delta u_n + \frac{\omega_0^2}{u_0^2}\left(\delta u_n \left(u_0 + \delta u_n\right)\left(2u_0 + \delta u_n\right)\right) = 0, \tag{28}$$

where k is the wavenumber and ω is the angular frequency. Expanding the last term on the left-hand side of Supplementary Eq. 28 in a Taylor series to the first order yields the homogeneous dispersion relation

$$\omega(k) = \pm\sqrt{2\omega_0^2 + \frac{2c_0^2}{a^2}(1-\cos ka)} = \pm\sqrt{\frac{8V_0 + 2Cu_0^2(1-\cos ka)}{mu_0^2}}. \tag{29}$$

By analytically rearranging Supplementary Eq. 29 for k, we have the homogeneous inverse dispersion relation

$$k(\omega) = \frac{1}{a}\arccos\left(1 + \frac{8V_0 - mu_0^2\omega^2}{2Cu_0^2}\right). \tag{30}$$

By analytically taking the derivative of Supplementary Eq. 29 with respect to k, we have the homogeneous acoustic wave group velocity

$$v_g = \frac{d\omega_+(k)}{dk} = \frac{Ca\sin ka}{2\sqrt{m\left(C\sin^2\frac{ka}{2} + \frac{2V_0}{u_0^2}\right)}}, \tag{31}$$

where ω_+ indicates Supplementary Eq. 29 with a positive sign.

Supplementary Note 3. NEWTON–RAPHSON METHOD FOR STATIC KINK STATES, DISCRETE MODAL SPECTRA, AND MODE SHAPES

To compute the static kink configurations in the discrete ϕ^4 chain and the KL chain, as well as the associated discrete modal frequencies and mode shapes (except for kink center rotor angle sweep results in the KL chain), we proceed as follows. For each set of parameters, a static kink solution is obtained numerically using the Newton–Raphson method[4], starting from an appropriate initial guess.

For discrete ϕ^4 chains, the initial guess for an intersite-centered kink is taken from the anti-continuum limit[5] and represented by the sequence

$$(-u_0,\ldots,-u_0,-u_0,u_0,u_0,\ldots,u_0).$$

For onsite-centered kinks, we instead use

$$(-u_0,\ldots,-u_0,-u_0,0,u_0,u_0,\ldots,u_0).$$

Analogous initial guesses are used in the KL chain. For intersite-centered kinks, we use

$$(\bar{\psi},\ldots,\bar{\psi},\bar{\psi},-\bar{\psi},-\bar{\psi},\ldots,-\bar{\psi}),$$

and for onsite-centered kinks, we use

$$(\bar{\psi},\ldots,\bar{\psi},\bar{\psi},0,-\bar{\psi},-\bar{\psi},\ldots,-\bar{\psi}).$$

In cases where convergence is not achieved for the KL chain, we generate the initial guess using the transfer and inverse transfer functions of the chain (Supplementary Eqs. 34, 35, 38, and 39), constrained to alternate rotor leaning directions as detailed in the previous section.

At each Newton-Raphson iteration, once the current state $\boldsymbol{\alpha} = (\alpha_1, \alpha_2, \ldots, \alpha_N)^T$ is specified ($\alpha = u$ for the discrete ϕ^4 chain and $\alpha = \psi$ for the KL chain), the residual force/torque balance vector $\mathbf{R}(\boldsymbol{\alpha})$ (force for the discrete ϕ^4 chain and torque for the KL chain) and the corresponding Jacobian $\mathbf{J}(\boldsymbol{\alpha}) = \partial \mathbf{R}(\boldsymbol{\alpha})/\partial \boldsymbol{\alpha}$ are evaluated. The correction vector $\boldsymbol{\Delta\alpha} = (\Delta\alpha_1, \Delta\alpha_2, \ldots, \Delta\alpha_N)^T$ is then obtained by solving

$$\mathbf{J}(\boldsymbol{\alpha})\boldsymbol{\Delta\alpha} \;=\; -\mathbf{R}(\boldsymbol{\alpha}), \qquad \boldsymbol{\alpha} \leftarrow \boldsymbol{\alpha} + \boldsymbol{\Delta\alpha}, \tag{32}$$

and the iteration continues until $||\boldsymbol{\Delta\alpha}|| \leq 10^{-8}$. Symmetry constraints are imposed near the kink center to stabilize the iteration. For example, for onsite-centered kinks, we enforce $\alpha_{n_{\text{kink}}} = 0$ and $\alpha_{n_{\text{kink}}-1} = -\alpha_{n_{\text{kink}}+1}$; for intersite-centered kink, we enforce $\alpha_{n_{\text{kink}}} = -\alpha_{n_{\text{kink}}+1}$, where n_{kink} denotes the kink center rotor index. The extent of the constraints (*i.e.*, how many rotors around the kink center are included) is adjusted depending on the quality of convergence.

Once a static kink configuration $\boldsymbol{\alpha}_{\text{kink}}$ is obtained, the equations of motion (Supplementary Eqs. 27 and 14) are linearized about this equilibrium. This yields the Jacobian matrix $\mathbf{J}_{\text{kink}}$

$$\mathbf{J}_{\text{kink}} = \left.\frac{\partial \mathbf{R}(\boldsymbol{\alpha})}{\partial \boldsymbol{\alpha}}\right|_{\boldsymbol{\alpha}=\boldsymbol{\alpha}_{\text{kink}}}, \tag{33}$$

which governs the evolution of small perturbations about $\boldsymbol{\alpha}_{\text{kink}}$. Solving the eigenvalue problem for $\mathbf{J}_{\text{kink}}$ provides the discrete modal frequencies (eigenfrequencies), as well as the corresponding mode shapes (eigenvectors), of the kink state $\boldsymbol{\alpha}_{\text{kink}}$. The resulting eigenfrequency spectrum consists of extended pass band modes together with localized modes residing inside the bandgap of infinite homogeneous chain (*i.e.*, in-gap modes).

Supplementary Note 4. DISPERSION RELATION EXAMPLES OF BOTH KL CHAINS AND ϕ^4 CHAINS

According to Supplementary Eqs. 16 and 17, the sign of effective coupling term c_2 depends on $2r\sin\bar{\psi} - a$, while the sign of c_1 does not. In other words, the choice of d can change the sign of ratio c_2/c_1, as a result, changing the concave direction of the dispersion curve. Notably, $d = 1$ leads to $c_2 = 0$, which corresponds to a vanishing penetration length of zero mode[1]. To demonstrate how the dispersion curve changes for d, we choose $\tilde{\tilde{l}} = 1.1$ and sweep $\tilde{r}$ from 0.3 to 1.3, which is equivalent to sweep d from 0.3873 to 2.5593, across all three phases, as shown as the red line in Supplementary Fig. 4**a**. The other parameters used are $a = 1$ m, $k_e = 100$ N m^{-1}, and $m = 1$ kg. Supplementary Fig. 4**b** shows the pass band range with eigenfrequencies of a finite, homogeneous chain with $N = 18$ rotors for this parametric sweep. There is no internal mode in the homogeneous chain, except for the zero mode. For KL chains in the F phase ($d < 1$), $c_2/c_1 > 0$ and the shape of dispersion curve resembles that of the SSH model (Supplementary Fig. 4**c**). When $d = 1$, $c_2/c_1 = 0$ and the shape of dispersion curve becomes flat (Supplementary Fig. 4**d**). As the chain is in the WF or S phase ($d > 1$), $c_2/c_1 < 0$ and the dispersion curve resembles that of the SSH model consisting of alternating positive and negative couplings exist (Supplementary Fig. 4**e** and **f**).

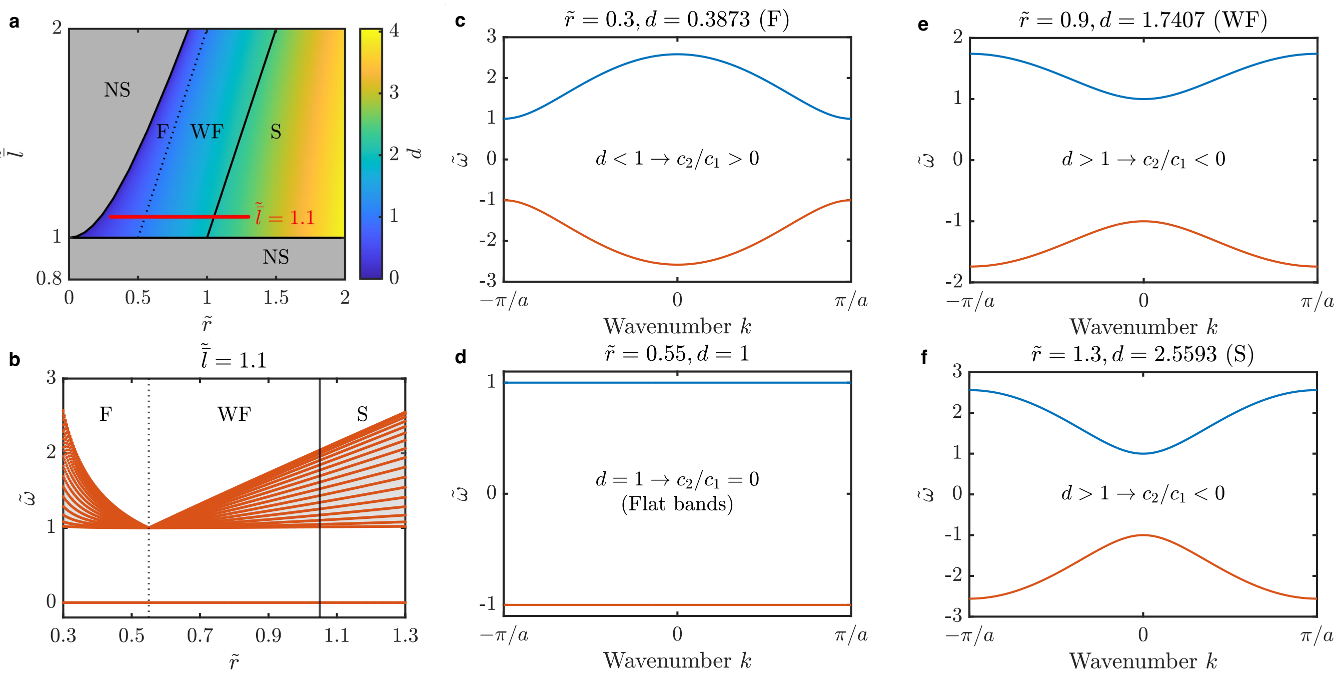

Supplementary Figure 4. **a** Geometric phase diagram of zero-energy kink states in the KL chain, with red line indicated swept cases ($\tilde{\tilde{l}} = 1.1$) in panel **b**. NS denotes "no state". **b** Normalized eigenfrequencies for finite homogeneous KL chain with 18 rotors. Shaded area indicates the pass band range of the infinite homogeneous chain. **c–f** Different examples of dispersion curves, including the flat band case for $d = 1$ (**d**).

An example of dispersion relation (Supplementary Eq. 29) of an infinite homogeneous ϕ^4 chain is shown in Supplementary Fig. 5, where $a = 1$ m, $u_0 = 0.25$ m, $V_0 = 0.015625$ J, $C = 3$ N m^{-1}, $m = 1$ kg, and the frequencies are normalized by the lower limit of the pass band ($\omega^2 > 0$).

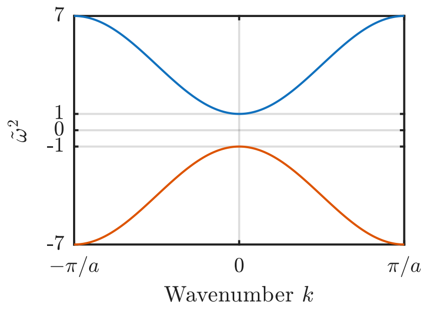

Supplementary Figure 5. Example dispersion relation (normalized squared frequency) of an infinite homogeneous ϕ^4 chain.

Supplementary Note 5. KINK SOLUTIONS AND THEIR SPECTRA IN DISCRETE ϕ^4 CHAINS

For comparison with finite KL chain's F- and WF-phase kinks, we consider a finite discrete ϕ^4 chain consisting of $N = 280$ particles with open boundary conditions. The static kink solutions and their stability are numerically calculated using the Newton-Raphson method[4], based on the equations of motion, and the corresponding Jacobian matrix. The parameters are arbitrarily chosen as $a = 1$ m, $u_0 = 0.25$ m, $V_0 = 0.015625$ J, $m = 1$ kg, and the kink width is adjusted by varying C. The normalized kink width of the discrete ϕ^4 kinks is defined as $\tilde{w}_0 = 1/\beta$, where β is obtained by fitting the chain with $u_n = \alpha \tanh(\beta(n-\gamma))$.

Supplementary Figs. 6 and 7 show the eigenfrequencies of discrete ϕ^4 chains containing intersite-centered and onsite-centered kink states shown in the insets as a function of kink width, respectively. Shaded areas denote the pass band ranges of the infinite homogeneous configurations. Each frequency is normalized by the lower limit of their respective pass band. We observe that the pass band collapses as $\tilde{w}_0$ approaches 0, which is the so-called anti-continuum limit of the discrete ϕ^4 chain[5] ($C \to 0$ in our case). In addition, we observe extra internal modes emerging in narrower (more discrete) kinks. In Supplementary Fig. 6, we can see that the lowest frequency internal mode (LFIM) of the intersite-centered ϕ^4 kinks decreases nearly linearly on a logarithmic scale as the kink width increases for $\tilde{w}_0 > 1$. This approach toward vanishingly low energy is consistent with the expected zero mode present in the continuum ϕ^4 kink[3,5–7]. In Supplementary Fig. 7, we can see that the LFIM of onsite-centered ϕ^4 kinks have negative values, indicating they are not stable solutions[5,7]. As the intersite-centered ϕ^4 kink approaches the continuum limit, these unstable modes approach zero, consistent with the expected zero-energy mode in the continuum ϕ^4 kink[3,5,7].

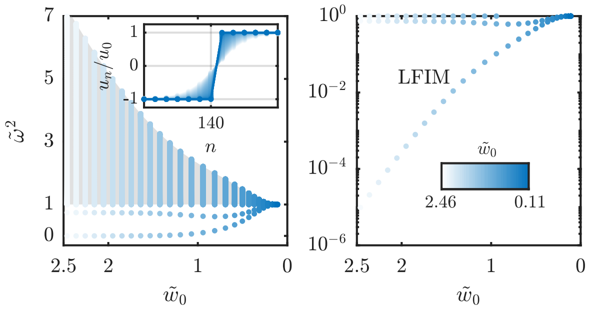

Supplementary Figure 6. Computed results of intersite-centered kink's shapes and spectra in discrete ϕ^4 chains. Normalized eigenfrequencies of intersite-centered kinks as a function of normalized kink widths for discrete ϕ^4 chains with 280 particles. Darker blue shading denotes smaller kink width (more discrete kink). Left: Shaded area indicates the pass band range of the infinite homogeneous chain. Insets show corresponding zoomed-in, normalized static kink solutions for discrete ϕ^4 chains (u_n/u_0). Right: Zoomed-in bandgap, where LFIM denotes the lowest frequency internal modes.

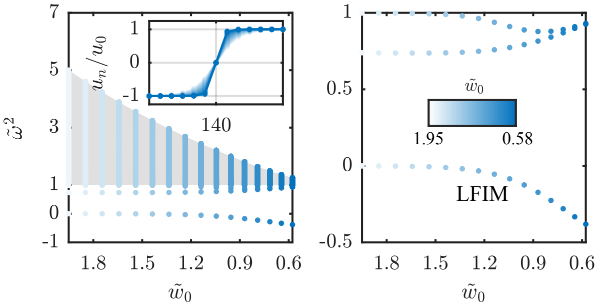

Supplementary Figure 7. Computed results of onsite-centered kink's shapes and spectra in discrete ϕ^4 chains. Normalized eigenfrequencies of onsite-centered kinks as a function of normalized kink widths for discrete ϕ^4 chains with 280 particles. Darker blue shading denotes smaller kink width (more discrete kink). Left: Shaded area indicates the pass band range of the infinite homogeneous chain. Insets show corresponding zoomed-in, normalized static kink solutions (u_n/u_0). Right: Zoomed-in bandgap, where LFIM denotes the lowest frequency internal modes.

Supplementary Note 6. SHORT-TIME DYNAMICS OF KINKS IN BOTH KL CHAINS AND DISCRETE ϕ^4 CHAINS

To characterize the energy barriers that the kinks (both in KL chain and discrete ϕ^4 chain) must overcome in order to initiate motion, we examine the short-time dynamics of kinks by assigning initial velocities to each site in the kink state, proportional to the eigenmode amplitudes of the zero mode in the KL chain (as shown in Fig. 2**b** and **c**) and the LFIM in the discrete ϕ^4 chain (as shown in Supplementary Fig. 6). Examples of these initial velocities are shown in the insets of Supplementary Fig. 8. Using these velocities, we characterize the input energy as $\sum_n mr^2\dot{\psi}_{n,t=0}^2/2$ for the KL chain and $\sum_n m\dot{u}_{n,t=0}^2/2$ for the discrete ϕ^4 chain. The kink velocity is determined by measuring the time it takes for the kink's center to shift by one site—specifically, when the 141st rotor (or particle) achieves the same angle (or displacement) as the 140th rotor (or particle). Supplementary Fig. 8**a** and **b** shows no energy barrier for the KL chain kink, as evidenced by the linear relationship between input energy and kink velocity, and the absence of a cutoff point. This confirms the absence of the PN barrier for the kink in the KL chain. In contrast, in Supplementary Fig. 8**c**, as the ϕ^4 kink becomes more discrete and the kink narrows (with narrower kinks denoted by darker blue shading), cutoff points appear at greater and greater input energies, below which the input energy is insufficient to achieve a kink velocity above the resolution limit.

Here, we explain how we perform the time domain simulations as shown in Supplementary Fig. 8. For the KL chain, the parameters are $a = 1$ m, $r = 1.5$ m, $k_e = 100$ N m^{-1}, $m = 1$ kg, $N = 280$, and the kink width is adjusted by varying $\bar{l}$. For the ϕ^4 model, the parameters are $a = 1$ m, $u_0 = 0.25$ m, $V_0 = 0.015625$ J, $m = 1$ kg, $N = 280$, and the kink width is adjusted by varying C. The total input energy to the kink is varied from 10^{-8} to 10^{-3} J and is distributed as initial velocities proportional to the lowest mode's mode shape for each kink state. The kink velocity is defined as the inverse of the time it takes for the kink's center to shift by one site, thus in the unit of a s^{-1}. Specifically, instead of fitting, we choose to quantify it as the time for the 140th rotor in the KL chain to rotate to the initial angle of the 141st rotor, or for the 140th particle in the discrete ϕ^4 model to move to the initial position of the 141st particle. The total simulation time is 10^3 s, which leads to a minimum velocity resolution of 10^{-3} a s^{-1}. Kinks that do not shift by one site during the total simulation time are assigned a velocity of zero.

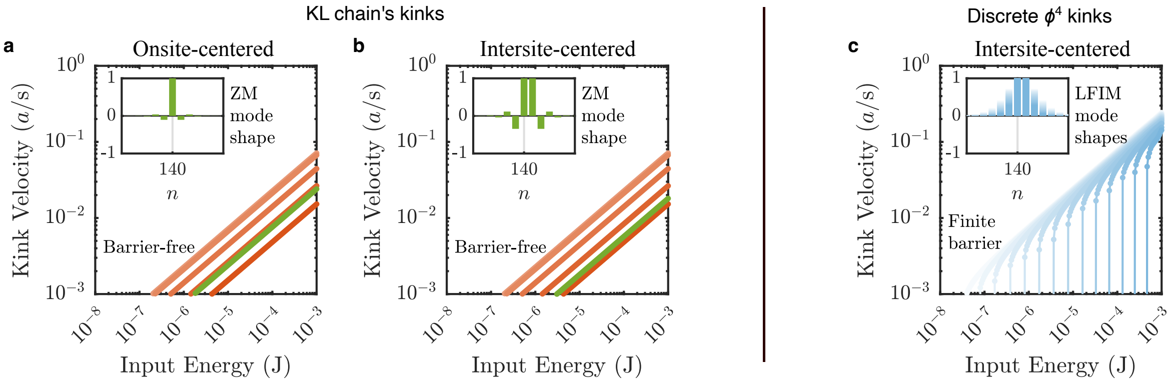

Supplementary Figure 8. Simulation results of kink's short-time dynamics in both KL chains and discrete ϕ^4 chains. Short-time kink propagation velocity versus initial input energy for **a** onsite-centered KL chain's kink states as shown in Fig. 2**b**, **b** intersite-centered KL chain's kink states as shown in Fig. 2**c**, and **c** intersite-centered discrete ϕ^4 kink states as shown in Supplementary Fig. 6, where the y-axis is truncated to 10^{-3} a s^{-1} due to the velocity resolution. The inset in **a** and **b** shows zoomed-in, normalized initial velocities for rotors in the KL chain with experimental unit cell geometry, whose magnitudes are based on the mode shapes of corresponding zero mode. The inset in **c** shows zoomed-in, normalized initial velocities for particles in the discrete ϕ^4 chain, whose magnitudes are based on the mode shapes of the LFIM for each chain configuration. In **a** and **b**, only cases with $\tilde{w}_0 = 0, 0.5, 1, 1.5, 2$, along with the experimental case, are presented. In **c**, only cases where $\tilde{w}_0 > 1$ are shown.

Supplementary Note 7. COMPARISON OF INTERNAL MODES IN KL CHAIN'S F/WF-PHASE KINKS AND ϕ^4 KINKS

7.1 Internal-mode frequencies of KL chain's F-phase kinks and ϕ^4 kinks as a function of kink width

We plot the internal-mode frequencies of F-phase kinks in the KL chain (orange) and compare them with those of kinks in the ϕ^4 chain (blue), as a function of kink width, as shown in Supplementary Fig. 9**a**. We find that as the kink width of the F-phase kink increases, only a single internal mode remains in addition to the zero mode in the KL chain. Its frequency asymptotically approaches the theoretical internal mode frequency, $\tilde{\omega}_s^2 = 3/4$ (black dashed line), predicted in the continuum ϕ^4 kink model[3,5–7]. This result further supports the mapping between the F-phase kink in the continuum limit of the KL chain and the ϕ^4 field theory, as introduced in Ref.[1]. In addition, as $\tilde{\omega}_0 \to 0$, all modes of the intersite-centered ϕ^4 kink, as well as all modes of the onsite-centered ϕ^4 kink except for the lowest-frequency internal mode, converge to a single point at $\tilde{\omega}^2 = 1$, which is known as the anti-continuum limit[5]. In contrast, for KL chain's F-phase kinks, multiple internal modes emerge in both intersite- and onsite-centered kink states as $\tilde{\omega}_0 \to 0$. In addition, we compare the kink profiles (Supplementary Fig. 9**b** and **d**) and the mode shapes of their corresponding lowest and second-lowest eigenmodes (Supplementary Fig. 9**c** and **e**) in both chains when the kinks have a normalized width close to 2, find similarities between F-phase kinks and ϕ^4 kinks as they approach their respective continuum limits.

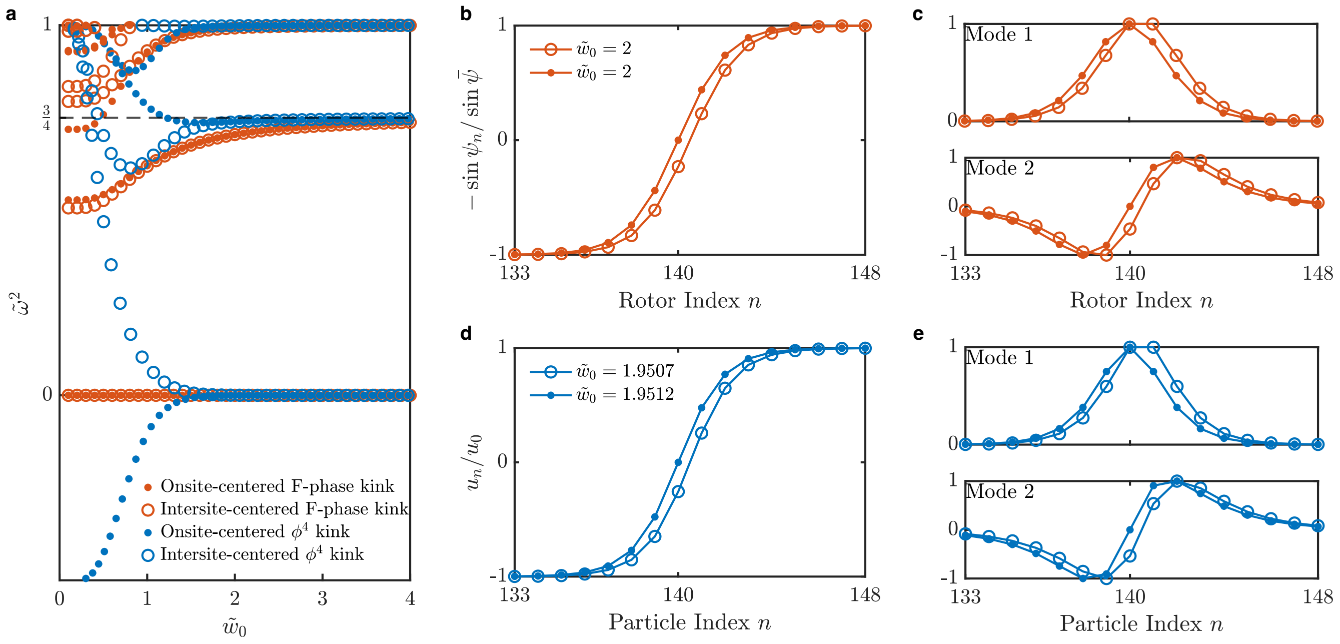

Supplementary Figure 9. **a** Eigenfrequencies of the internal modes as a function of kink width for KL chain's F-phase kinks (orange) and ϕ^4 kinks (blue). **b–e** Zoomed-in kink profiles (**b** and **d**) and normalized mode shapes (**c** and **e**) of their lowest (Mode 1) and second-lowest eigenmodes (Mode 2) for a normalized kink width around 2. Results for the onsite-centered and intersite-centered kinks are represented by dots and circles, respectively.

7.2 Mode shapes of internal modes of highly discrete intersite-centered kinks

In Supplementary Fig. 10, we visualize the mode shapes of the internal modes of highly discrete intersite-centered kinks in the KL chain for $\tilde{r} = 1.5$ and selected values of d that yield small $\tilde{w}_0$.

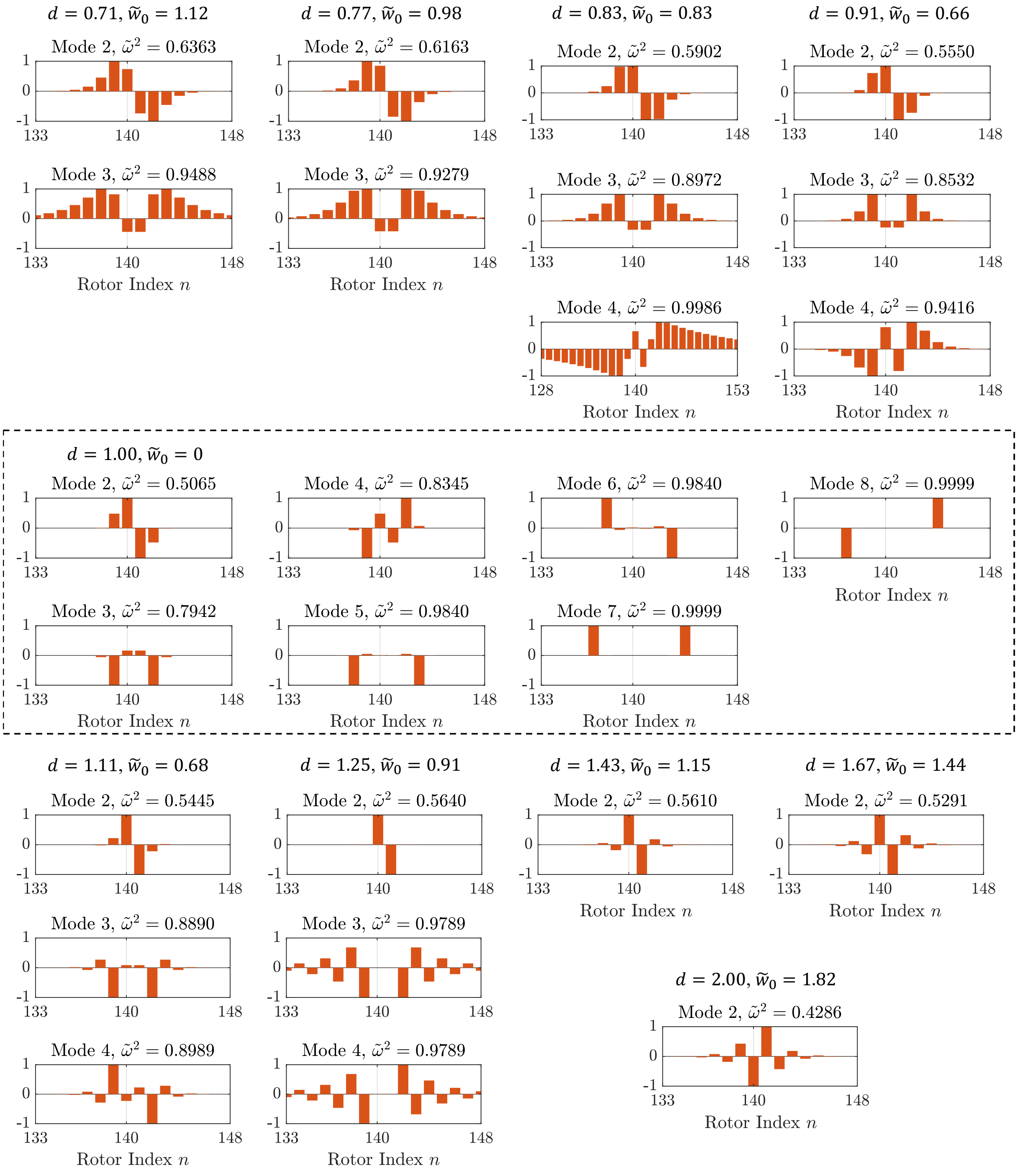

Supplementary Figure 10. Zoomed-in mode shapes of internal modes in the KL chain with a highly discrete central intersite-centered kink.

In Supplementary Fig. 11, we also visualize the mode shapes of the internal modes of highly discrete intersite-centered ϕ^4 kinks for selected values of $\tilde{C} \equiv Ca^2/V_0$ (shown rounded to two decimal places in the figure for readability) that yield small $\tilde{w}_0$.

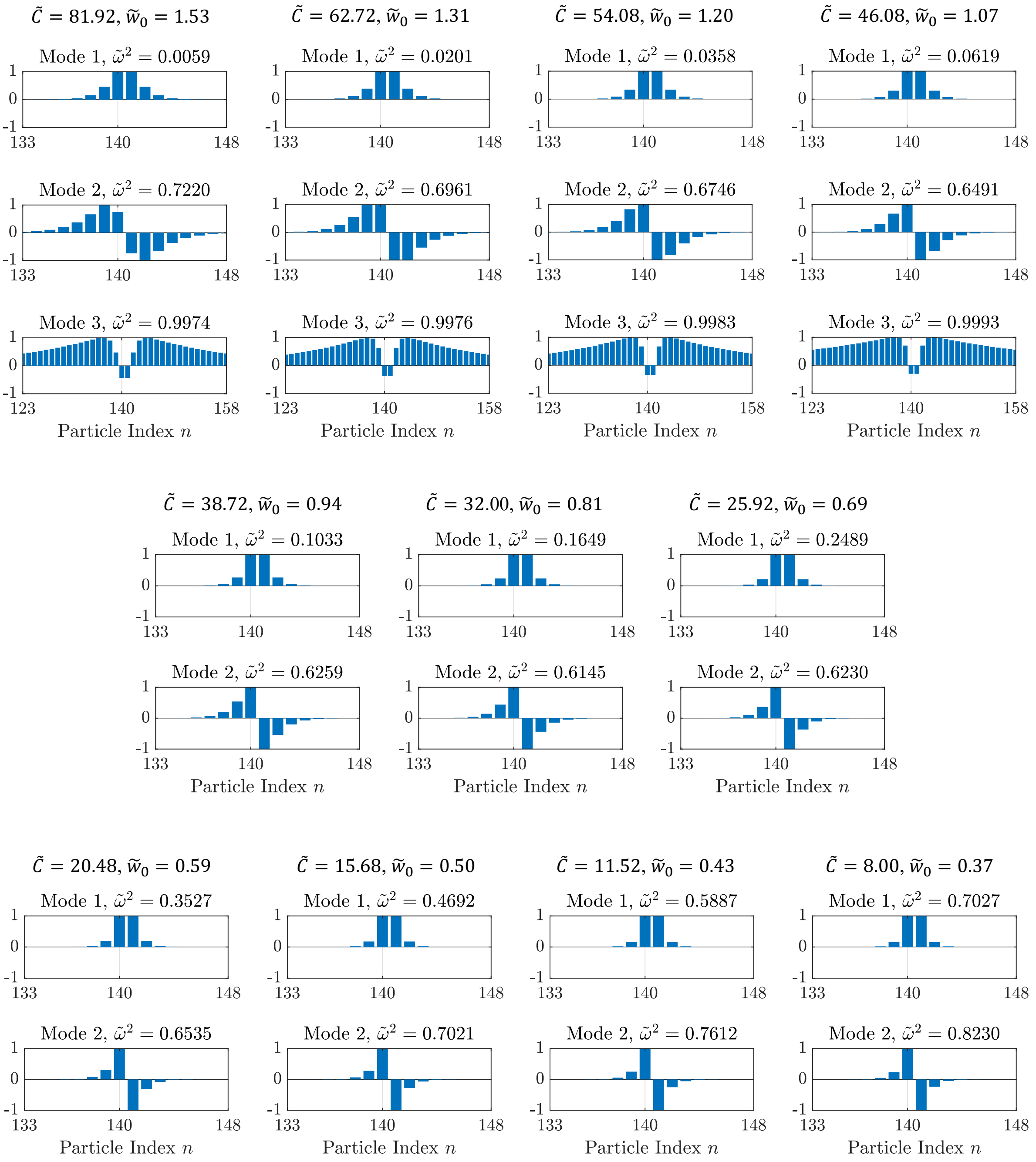

Supplementary Figure 11. Zoomed-in mode shapes of internal modes in the ϕ^4 chain with a highly discrete central intersite-centered kink.

7.3 Mode shapes of internal modes of highly discrete onsite-centered kinks

In Supplementary Fig. 12, we visualize the mode shapes of the internal modes of highly discrete KL chain's onsite-centered kink for $\tilde{r} = 1.5$ and selected values of d.

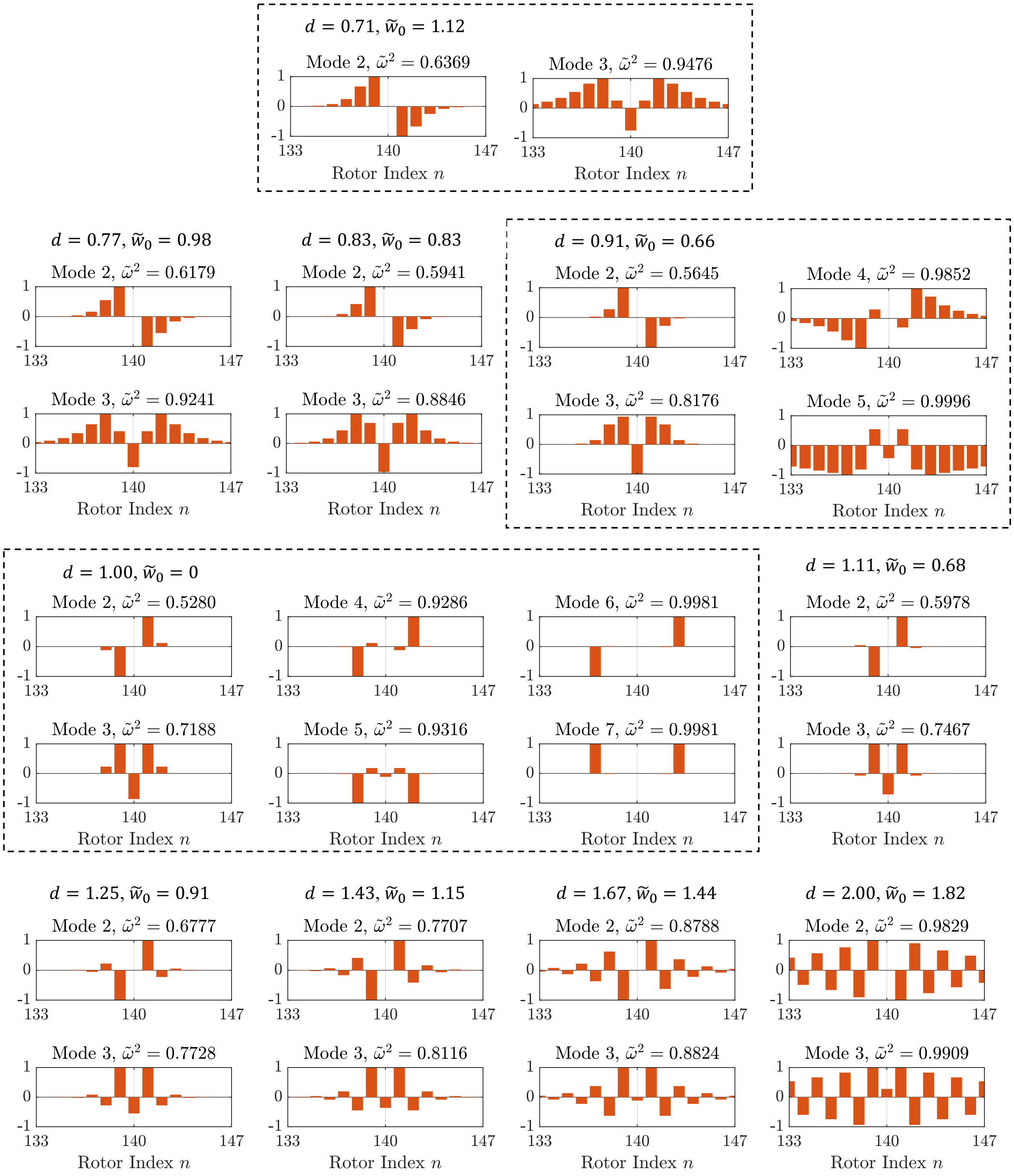

Supplementary Figure 12. Zoomed-in mode shapes of internal modes in the KL chain with a highly discrete central onsite-centered kink.

In Supplementary Fig. 13 we also visualize the mode shapes of the internal modes, including the unstable lowest frequency internal mode, of highly discrete onsite-centered ϕ^4 kinks, presenting a subset of the cases shown in Supplementary Fig. 7.

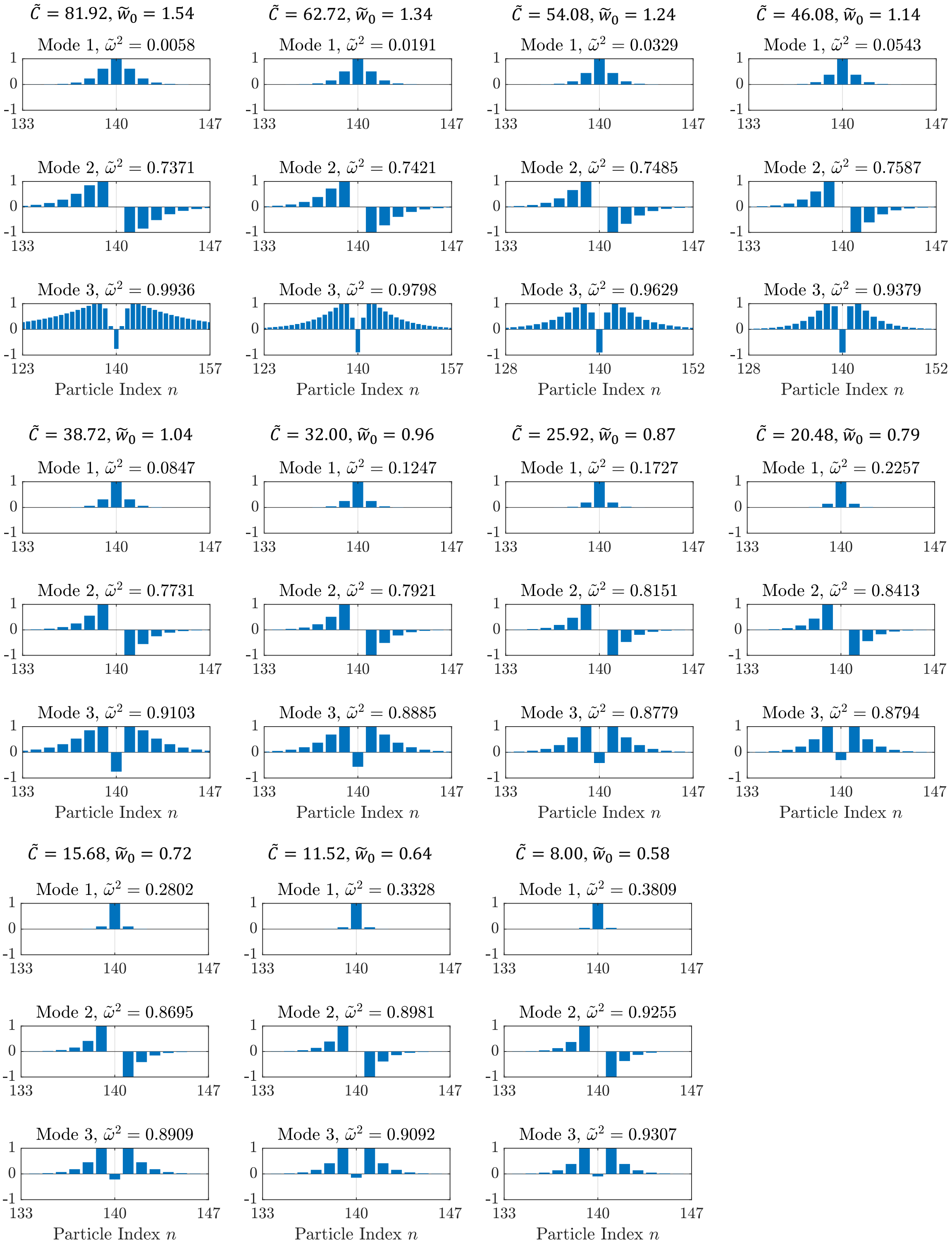

Supplementary Figure 13. Zoomed-in mode shapes of internal modes in the ϕ^4 model with a highly discrete central onsite-centered kink.

Supplementary Note 8. TRANSFER AND INVERSE TRANSFER FUNCTIONS OF THE KL CHAIN

Here we describe how to geometrically generate the KL chain configuration with a central kink. There are two constraints: (1) Springs remain unstretched; (2) Rotors lean towards alternating side of the chain's central line.

From the first constraint, we analytically solve $\bar{l}^2 = (a - r\sin\theta_n + r\sin\theta_{n+1})^2 + (r\cos\theta_n - r\cos\theta_{n+1})^2$, and obtain two *forward* transfer functions in the form of $\theta_{n+1} = f(\theta_n)$,

$$f_1(\theta_n) = \arctan\frac{Y_{f,+}}{X_{f,+}} \tag{34}$$

and

$$f_2(\theta_n) = \arctan\frac{Y_{f,-}}{X_{f,-}}, \tag{35}$$

where

$$X_{f,\pm} = \frac{r^3\left(a^2 + 2r^2 - 2ar\sin\theta_n - \bar{l}^2\right)\cos\theta_n \pm (r\tan\theta_n - a\sec\theta_n)\gamma_f}{r^3\left(a^2 + r^2 - 2ar\sin\theta_n\right)} \tag{36}$$

and

$$Y_{f,\pm} = \frac{r(-a + r\sin\theta_n)(a^2 + 2r^2 - 2ar\sin\theta_n - \bar{l}^2) \pm (-1)\gamma_f}{r^2\left(a^2 + r^2 - 2ar\sin\theta_n\right)} \tag{37}$$

with $\gamma_f = \sqrt{r^4\cos^2\theta_n\left(\left(2r\bar{l}\right)^2 - \left(\bar{l}^2 - a\left(a - 2r\sin\theta_n\right)\right)^2\right)}$. Each of the transfer functions $f_1(\theta_n)$ and $f_2(\theta_n)$ results a θ_{n+1} solution, which can be off by $\pm\pi$ due to the periodicity of the arctan function. With geometric parameters, by adjusting $\pm\pi$ shift and checking if $\bar{l}^2 - \left((a - r\sin\theta_n + r\sin\theta_{n+1})^2 + (r\cos\theta_n - r\cos\theta_{n+1})^2\right)$ is zero (we use 10^{-12} as a numerical threshold in MATLAB), we can find two θ_{n+1} solutions, $\theta_{n+1}^{(f_1)}$ and $\theta_{n+1}^{(f_2)}$, from $f_1(\theta_n)$ and $f_2(\theta_n)$ individually, that result in no spring stretching. If $\mod(\theta_{n+1}^{(f_1)} - \theta_n, 2\pi) < \mod(\theta_{n+1}^{(f_2)} - \theta_n, 2\pi)$, we call $\theta_{n+1}^{(f_1)}$ the counterclockwise (CCW) solution and $\theta_{n+1}^{(f_2)}$ the clockwise (CW) solution. Otherwise, we call $\theta_{n+1}^{(f_2)}$ the CCW solution and $\theta_{n+1}^{(f_1)}$ the CW solution.

Similarly, by analytically solving $\bar{l}^2 = (a - r\sin\theta_{n-1} + r\sin\theta_n)^2 + (r\cos\theta_{n-1} - r\cos\theta_n)^2$, we obtain two *backward* inverse transfer functions in the form of $\theta_{n-1} = g(\theta_n)$,

$$g_1(\theta_n) = \arctan\frac{Y_{g,+}}{X_{g,+}} \tag{38}$$

and

$$g_2(\theta_n) = \arctan\frac{Y_{g,-}}{X_{g,-}}, \tag{39}$$

where

$$X_{g,\pm} = \frac{r^3\left(a^2 + 2r^2 + 2ar\sin\theta_n - \bar{l}^2\right)\cos\theta_n \pm (r\tan\theta_n + a\sec\theta_n)\gamma_g}{r^3\left(a^2 + r^2 + 2ar\sin\theta_n\right)} \tag{40}$$

and

$$Y_{g,\pm} = \frac{r(a + r\sin\theta_n)(a^2 + 2r^2 + 2ar\sin\theta_n - \bar{l}^2) \pm (-1)\gamma_g}{r^2\left(a^2 + r^2 + 2ar\sin\theta_n\right)} \tag{41}$$

with $\gamma_g = \sqrt{r^4\cos^2\theta_n\left(\left(2r\bar{l}\right)^2 - \left(\bar{l}^2 - a\left(a + 2r\sin\theta_n\right)\right)^2\right)}$. With gemometric parameters, By adjusting $\pm\pi$ shift and checking if $\bar{l}^2 - \left((a - r\sin\theta_{n-1} + r\sin\theta_n)^2 + (r\cos\theta_{n-1} - r\cos\theta_n)^2\right)$ is zero , we can find $\theta_{n-1}^{(g_1)}$ and $\theta_{n-1}^{(g_2)}$, from $g_1(\theta_n)$ and

$g_2(\theta_n)$ individually, that result in no spring stretching. If $\mod(\theta_{n-1}^{(g_1)} - \theta_n, 2\pi) < \mod(\theta_{n-1}^{(g_2)} - \theta_n, 2\pi)$, we call $\theta_{n-1}^{(g_1)}$ the inverse-counterclockwise (ICCW) solution and $\theta_{n-1}^{(g_2)}$ the inverse-clockwise (ICW) solution. Otherwise, we call $\theta_{n-1}^{(g_2)}$ the ICCW solution and $\theta_{n-1}^{(g_1)}$ the ICW solution.

We use the second constraint to choose the proper solution at each site, except in Section XI. For the KL chain with N rotors with the kink placed somewhere in the chain, we generate the configuration of the chain starting from a rotor with a given angle, and using the transfer and inverse transfer functions to generate rotor angles on both sides. For generating the right side of the kink, we take alternating angular solutions in the pattern of CW, CCW, CW, CCW,... for each rotor as counting away from the center of the kink. For generating the left side of the kink, we take solutions in the pattern of ICCW, ICW, ICCW, ICW, ... for each rotor as counting away from the center of the kink. This process ensures that, with the kink in the chain, the rotors still lean towards alternating side of the central line, which is the configuration that can be evolved from a homogeneous state quasi-statically.

Supplementary Note 9. CONTINUOUS FAMILY OF INTERNAL MODES FOR KINK SOLUTIONS BETWEEN TWO SITES IN KL CHAINS

9.1 Additional computed results

To demonstrate that the KL chain supports an infinite number of static kink solutions due to the presence of a zero-energy mode, in Supplementary Fig. 14, we plot the internal modes as the onsite-centered kink ($N = 280$ and $\psi_{140} = 0$) shifts by up to a distance $a/2$ for $\tilde{r} = 1.5$ and three different cases of d (one in the F phase and the others in the WF phase). A continuous family of zero modes is observed alongside the evolution of internal modes for the KL chain, in contrast to the discrete ϕ^4 model, where only onsite- or intersite-centered steady kink solutions are found[3,5,7]. The internal-mode frequencies exhibit sharper variations during shifts for larger d.

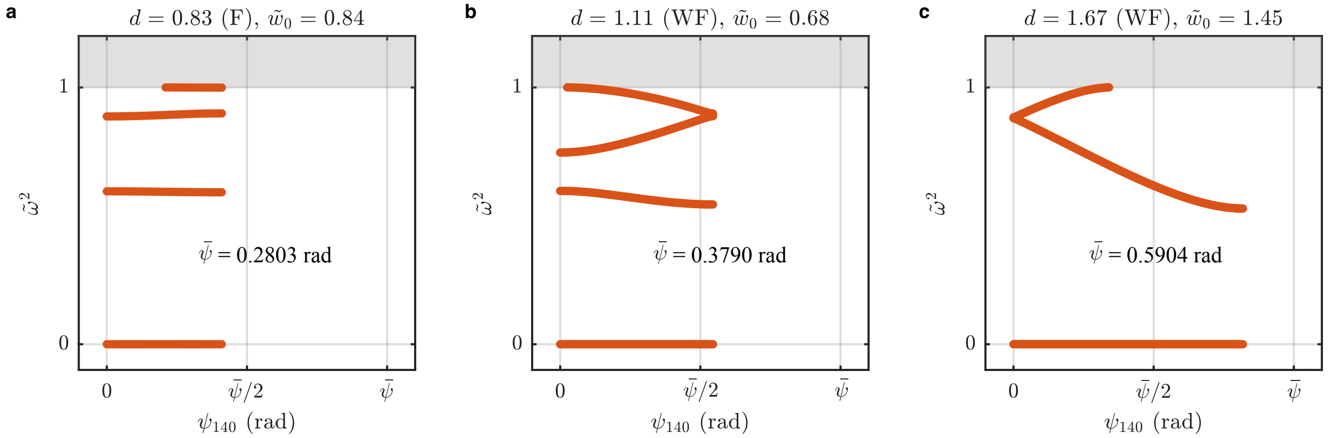

Supplementary Figure 14. Evolution of in-gap modes as a function of the kink's center rotor angle (ψ_{140}) in the KL chains with $N = 280$ rotors and $\tilde{r} = 1.5$ for $d = 0.83$, $d = 1.11$, and $d = 1.67$. Shaded area indicates the pass band range of the infinite homogeneous chain.

9.2 Computational method

Here we explain the details on generating eigenmodes for kink solutions during shifts in the KL chain. We consider the KL chain with $N = 280$ rotors and use transfer and inverse transfer functions (Supplementary Eqs. 34, 35, 38, and 39) to generate the chain from the kink's center rotor (140th rotor) forwards and backwards. By sweeping the kink's center rotor angle (ψ_{140}) from 0 upwards, we can generate the chain configuration with the kink center quasi-statically shifting between the onsite-centered and intersite-centered states. We calculate the eigenfrequencies by using the generated chain configuration and the dynamical matrix. We fit displacement variables with a hyperbolic tangent function to get the kink's center position, and the data are truncated to include only the range corresponding to the shift between onsite-centered and intersite-centered states. For the results shown in Fig. 2**d**, same process is applied.

To derive the dynamical matrix of the KL chain, we consider a finite chain with N rotors and introduce an infinitesimal angular perturbation, $\boldsymbol{\delta\theta} = (\delta\theta_1, \delta\theta_2, \ldots, \delta\theta_N)^T$, where counterclockwise displacements are taken as positive. This perturbation is applied to the kink configuration of the chain, denoted by $\boldsymbol{\theta}_{\text{kink}} = (\theta_{1,0}, \theta_{2,0}, \ldots, \theta_{N,0})^T$. Expanding Supplementary Eq. 14 using a Taylor series, we obtain the linearized equation of motion: $\mathbf{M}\partial_{tt}(\boldsymbol{\theta}_{\text{kink}} + \boldsymbol{\delta\theta}) \approx \mathbf{K}(\boldsymbol{\theta}_{\text{kink}})(\boldsymbol{\delta\theta})$, where $\mathbf{M}$ is the mass matrix and $\mathbf{K}$ is the stiffness matrix evaluated at the kink state $\boldsymbol{\theta}_{\text{kink}}$. Applying Bloch's theorem, we obtain the eigenvalue equation: $\mathbf{D}(\boldsymbol{\theta}_{\text{kink}})\boldsymbol{\delta\theta} = \omega^2\boldsymbol{\delta\theta}$, where $\mathbf{D}(\boldsymbol{\theta}_{\text{kink}}) = -\mathbf{M}^{-1}\mathbf{K}$ is the dynamical matrix associated with the kink state $\boldsymbol{\theta}_{\text{kink}}$. By specifying a chain configuration for a kink state $\boldsymbol{\theta}_{\text{kink}}$ and numerically solving its eigenproblem, we obtain the discrete modes of that kink state.

Supplementary Note 10. ADDITIONAL DETAILS OF TIME-DOMAIN SIMULATIONS

10.1 Setup

Except for the perturbative predictions presented in later sections (performed in Mathematica with optimal sampling rates), numerical simulations of kink dynamics for both the KL and discrete ϕ^4 chains are carried out using the *ode45* function in MATLAB, with 'RelTol' and 'AbsTol' set to 10^{-6}. Unless otherwise stated, the initial displacements and velocities are set to zero.

10.2 Tracking kink center position

To track the kink center position during the time domain simulation, we fit the displacement variables (ψ_n for KL chain's kinks and u_n for ϕ^4 kinks) at each timestamp with a hyperbolic tangent function of the form $f(n) = \alpha \tanh(\beta(n-\gamma))$, using the *fit* function in MATLAB, where the coefficient γ is what we use to characterize the center position of the kink. The same approach is applied to the experimental results.

10.3 Prediction of acoustic wave packet center

To predict the center of the acoustic wave packet in the time-domain simulations for both KL and discrete ϕ^4 chains, we first determine the excited wavenumber from the excitation frequency using the corresponding homogeneous inverse dispersion relation for each model (see Supplementary Eq. 30 and Supplementary Eq. 20). We then substituted this wavenumber into Supplementary Eq. 31 and Supplementary Eq. 21 to calculate the corresponding group velocity for each homogeneous chain model.

Supplementary Note 11. OSCILLATORY TRAJECTORY OF DISCRETE KL CHAIN'S KINK

To better examine whether the oscillatory trajectory of the KL chain's kink with experimental unit cell geometry ($\tilde{r} = 1.5$, $d_{\mathrm{exp}} = 2.0296$) is genuine rather than an artifact, Supplementary Fig. 15 shows an enlarged version of the inset from Fig. 3**i**, plotted in terms of normalized angular velocity.

Based on the velocity data, we observe that as the kink propagates, the rotor near the kink's center rotates at an almost constant speed, as indicated by the consistent (blue) colors. When the kink's center is primarily influenced by a single rotor, its movement slows down. However, as the kink transitions between two neighboring rotors, its speed increases. This variation in speed results in the oscillatory trajectory that we observe.

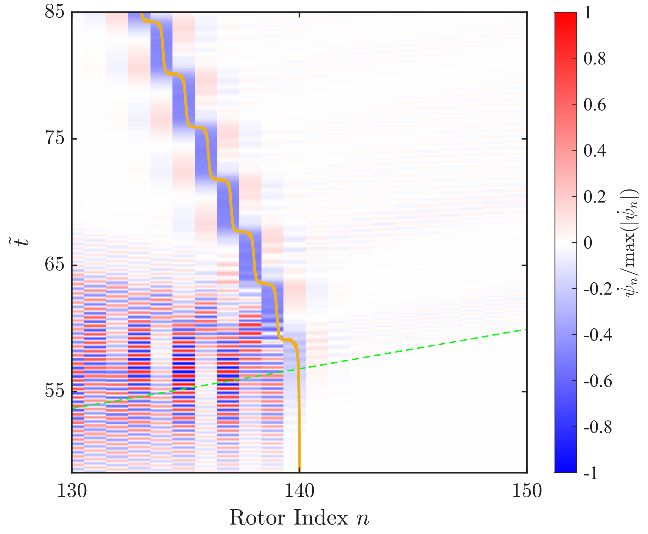

Supplementary Figure 15. Enlarged version of the inset from Fig. 3**i**, in terms of normalized angular velocity. Green dashed lines denote the predicted wave packet center position based on the group velocity at the excitation frequency in the infinite homogeneous chain and yellow solid lines indicate the fitted kink center position.

Supplementary Note 12. SIMULATIONS OF ACOUSTIC-WAVE–KINK INTERACTION IN ϕ^4 CHAINS

We show numerical simulations that demonstrate the dynamical behaviors of a single intersite-centered kink in the ϕ^4 model when interacting with acoustic wave packets, highlighting the differences between kinks in the near-continuum regime (Supplementary Fig. 16**a** and **b**) and the discrete regime far from the continuum limit (Supplementary Fig. 16**c** and **d**). While in both cases the kink shifts in the direction of the incoming wave packets initiated by the interaction, the near-continuum kink exhibits minimal acoustic wave scattering and only moves during the interaction (as reported in Ref.[8]). In contrast, the discrete kink partially reflects acoustic waves and continues to move, with radiation backward even after acoustic waves have moved away from it. This backward acoustic wave radiation by a moving lattice kink has been studied in discrete nonlinear systems[9]. We also see in the inset of Supplementary Fig. 16**d** that the discrete ϕ^4 kink has a somewhat oscillatory trajectory as it propagates. Eventually, the kink becomes pinned due to the PN barrier and high discreteness. It has also been shown that in discrete sG systems, the higher the discreteness, the faster the deceleration and pinning of the kink[10].

The kink is positioned between the 500th and 501st particles, with its discreteness adjusted through C. The simulation parameters are arbitrarily chosen: $a = 1$ m, $m = 1$ kg, $u_0 = 0.25$ m, $V_0 = 0.015625$ J, and $N = 1000$. For the kink in the near-continuum regime, we set $C = 80$ N m^{-1}, while for the kink in the discrete regime, $C = 0.9$ N m^{-1}. A Gaussian-modulated sinusoidal wave packet is introduced from the 450th particle as a driving force, described by $F(t) = F_0 e^{-(t-T/2)^2/(2\sigma_t^2)} \sin \omega_{\text{driven}} t$, where $T = 2\pi N_{\text{cycle}}/\omega_{\text{driven}}$, $t_0 = T/2$, $\sigma_t = 0.15T$, $N_{\text{cycle}} = 40$, and ω_{driven} equals to the median of eigenfrequencies. The amplitudes of the driving force for both cases are set to $\tilde{F}_0 \equiv F_0/(Ca) = 0.065$.

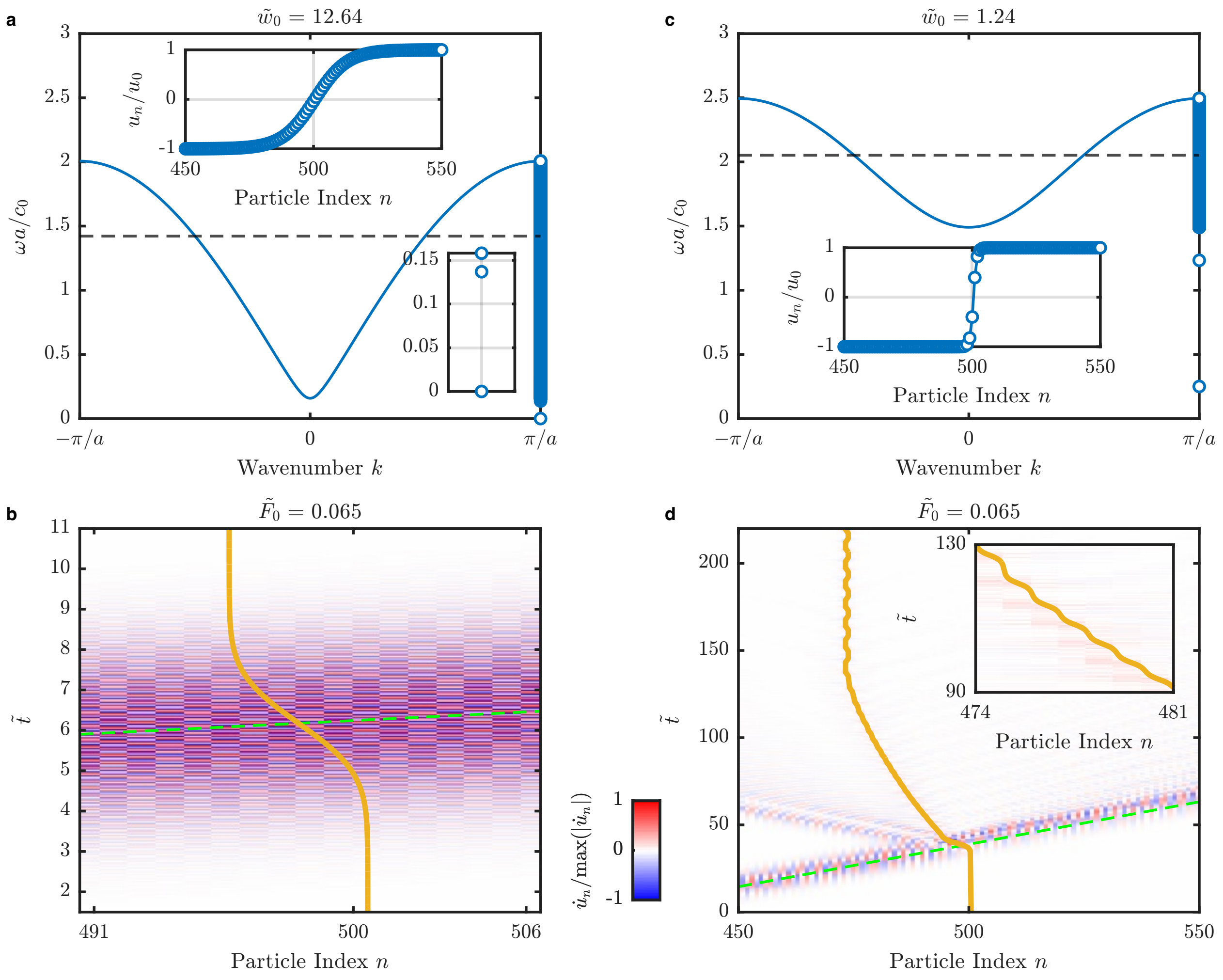

Supplementary Figure 16. Simulated kink dynamics in the ϕ^4 model triggered by acoustic-wave–kink interaction, for a kink near the continuum limit (**a** and **b**) and a discrete kink far from the continuum limit (**c** and **d**). **a**, **c** Dispersion relation of homogeneous chains with finite eigenmodes overlapped at $k = \pi/a$. The black dashed line denotes the excitation frequency. Insets show the zoomed-in initial kink equilibrium configuration, with an additional inset in **a** highlighting the zero mode and internal modes in the bandgap. **b**, **d** Zoomed-in spatiotemporal chain responses in terms of normalized velocity. Green dashed lines denote the predicted wave packet center position based on the group velocity at the excitation frequency in the infinite homogeneous chain and yellow solid lines indicate the fitted kink center position.

We also present an additional simulation of the same system in Supplementary Fig. 16**b**, but with a smaller excitation amplitude $\tilde{F}_0 = 0.03$. In this case, acoustic wave packets do not manage to trigger the movement of the kink, and most of acoustic waves are reflected back, with a small amount of transmission.

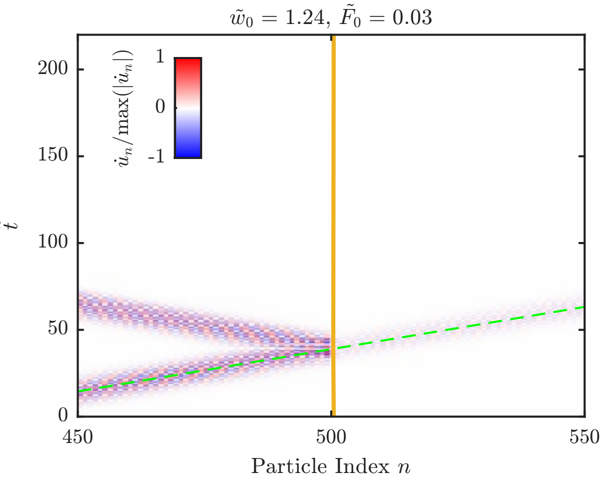

Supplementary Figure 17. Zoomed-in simulated spatiotemporal response in the discrete ϕ^4 model with lower excitation amplitude. The green dashed line denotes the predicted wave packet center position based on the group velocity at the excitation frequency in the infinite homogeneous chain and the yellow solid line indicates the fitted kink center position.

Supplementary Note 13. PERTURBATION CALCULATION FOR SHORT-TIME ACOUSTIC-WAVE–KINK INTERACTION

We introduce a first-order perturbation theory to predict the short-time dynamics of the KL chain kink due to its interaction with acoustic waves, specifically interested in whether the interaction results in the attraction or repulsion type dynamics shortly after the acoustic wave packet reaches the kink. We also perform numerical examination of this theory.

13.1 Microscopic model: mode coupling

Here, we show the relation between the two representations of the KL chain. One is the usual angular coordinates $\boldsymbol{\theta} = \left(\theta^1, ..., \theta^N\right)^T$, the other is the mode amplitudes' representation $\boldsymbol{\varphi} = \left(\varphi^0, ..., \varphi^{N-1}\right)^T$.

A configuration of the KL chain is represented by a vector $\boldsymbol{\theta}$ with N components (N is the number of rotors). The Lagrangian of the system is

$$L(\boldsymbol{\theta}) = \frac{mr^2}{2} \sum_{i=1}^{N} \left(\frac{d\theta^i}{dt}\right)^2 - V(\boldsymbol{\theta}), \tag{42}$$

where mr^2 is the rotors' moment of inertia. This Lagrangian has a static kink solution $\boldsymbol{\theta}_{\text{kink}}$ with zero potential energy $V(\boldsymbol{\theta}_{\text{kink}}) = 0$. We look for small deviations $\boldsymbol{\delta\theta}(t)$ from the static kink solution by expanding a general configuration around $\boldsymbol{\theta}_{\text{kink}}$:

$$\boldsymbol{\theta}(t) = \boldsymbol{\theta}_{\text{kink}} + \boldsymbol{\delta\theta}(t). \tag{43}$$

The Lagrangian for the field $\boldsymbol{\delta\theta}(t)$ is:

$$L(\boldsymbol{\delta\theta}) = \frac{mr^2}{2} \sum_{i=1}^{N} \left(\frac{d\delta\theta^i}{dt}\right)^2 - V(\boldsymbol{\theta}_{\text{kink}} + \boldsymbol{\delta\theta}). \tag{44}$$

The Euler-Lagrange equations for the field $\delta\theta^i$ are

$$mr^2 \frac{\mathrm{d}^2\delta\theta^i}{\mathrm{d}t^2} = -\frac{\partial V(\boldsymbol{\theta}_{\text{kink}} + \boldsymbol{\delta\theta})}{\partial \delta\theta^i}. \tag{45}$$

By expanding the potential around the static kink configuration $\boldsymbol{\theta}_{\text{kink}}$, we find the equations of motion of the field $\delta\theta^i$ in the background of the kink:

$$mr^2 \frac{\mathrm{d}^2\delta\theta^i}{\mathrm{d}t^2} = -\frac{\partial}{\partial\delta\theta^i}\left(\frac{1}{2}\frac{\partial^2 V(\boldsymbol{\theta}_{\text{kink}})}{\partial\delta\theta^j\partial\delta\theta^k}\delta\theta^j\delta\theta^k + \frac{1}{6}\frac{\partial^3 V(\boldsymbol{\theta}_{\text{kink}})}{\partial\delta\theta^j\partial\delta\theta^k\partial\delta\theta^l}\delta\theta^j\delta\theta^k\delta\theta^l + ...\right), \tag{46}$$

where we dropped the zeroth and first order terms because the static kink has zero potential energy and is an extremum of V. To simplify the notation, from now on we will introduce the contracted notation

$$V_{,ij} \equiv \frac{\partial^2 V(\boldsymbol{\theta}_{\text{kink}})}{\partial\delta\theta^i\partial\delta\theta^j} \quad , \quad V_{,ijk} \equiv \frac{\partial^3 V(\boldsymbol{\theta}_{\text{kink}})}{\partial\delta\theta^i\partial\delta\theta^j\partial\delta\theta^k}, \tag{47}$$

and so on. Note that derivatives commute. All these expressions are evaluated on the static kink solution $\boldsymbol{\theta}_{\text{kink}}$ (in other words, at $\boldsymbol{\delta\theta} = 0$) and do not depend on $\boldsymbol{\delta\theta}$. After taking the derivative $\partial_{\delta\theta^i}$ in Supplementary Eq. 46 and dropping the higher order terms, the equation of motion for the ith angle is (up to second order in $\boldsymbol{\delta\theta}$):

$$mr^2 \frac{\mathrm{d}^2\delta\theta^i}{\mathrm{d}t^2} = -V_{,ij}\delta\theta^j - \frac{1}{6}(V_{,ijk} + V_{,kij} + V_{,jki})\delta\theta^j\delta\theta^k = -V_{,ij}\delta\theta^j - \frac{1}{2}V_{,ijk}\delta\theta^j\delta\theta^k, \tag{48}$$

where we used the commutativity of partial derivatives. If we left the derivative $\partial_{\delta\theta^i}$ unevaluated in Supplementary Eq. 48, it would read:

$$mr^2 \frac{\mathrm{d}^2\delta\theta^i}{\mathrm{d}t^2} = -\frac{1}{2}V_{,jk}\frac{\partial(\delta\theta^j\delta\theta^k)}{\partial\delta\theta^i} - \frac{1}{6}V_{,jkl}\frac{\partial(\delta\theta^j\delta\theta^k\delta\theta^l)}{\partial\delta\theta^i}. \tag{49}$$

The term $V_{,jk}$ is the dynamical matrix of the KL chain in the static kink configuration:

$$V_{,jk} = \frac{\partial^2 V(\boldsymbol{\theta}_{\text{kink}})}{\partial\delta\theta^j \partial\delta\theta^k} \equiv D_{jk}. \tag{50}$$

This matrix has a set of N eigenvectors $\{\mathbf{e}_a\}$, $a = 0,...,N-1$ defined by the eigenvalue problem $V_{,ij}e_a^j = \omega_a^2 e_a^i$, where $\{\omega_a\}$ are the eigenfrequencies of the KL chain in the static kink configuration. Notice that the index $i = 1,...,N$ runs over the rotors while the index a spans the eigenspace of $V_{,ij}$. The set of eigenvectors (also called eigenmodes) is a complete basis so we expand the field $\boldsymbol{\delta\theta}$:

$$\boldsymbol{\delta\theta}(t) = \sum_{a=0}^{N-1} \varphi^a(t)\mathbf{e}_a. \tag{51}$$

The φ^a are the amplitudes of the configuration $\boldsymbol{\delta\theta}$ along the eigenvectors of the dynamical matrix. Notice that since Supplementary Eq. 51 is linear, by definition we have

$$\mathbf{e}_a = \frac{\partial\boldsymbol{\delta\theta}}{\partial\varphi^a}, \tag{52}$$

which will be used below to change variables in the derivatives of the potential. The interpretation of the eigenvectors depends on the properties of the energy spectrum. In the simplest case, there is one state at zero energy $\omega_0 = 0$, one isolated energy mode at frequency ω_1 and a continuum of states with narrowly spaced frequencies ω_p with $\omega_p > \omega_1$. Then, $\mathbf{e}_0$ is the zero-frequency (*i.e.*, zero-energy) translation mode, $\mathbf{e}_1$ is the lowest frequency internal mode at frequency ω_1, and the continuum of states are generally labeled by a continuous parameter p with frequencies $\omega_p > \omega_1$.

In more general situations, there can be more than one internal mode in the gap. In this case, the internal modes will have frequencies $\{\omega_1, \omega_2, ..., \omega_{N_{\text{int}}}\}$ where N_{int} is the number of internal modes. The pass band is the set of frequencies ω_p, where $\omega_{N_{\text{int}}}$ is strictly smaller than the lowest frequency.

Now we can perform a change of variables, from the angular variables $\boldsymbol{\delta\theta}$ to their amplitudes $\boldsymbol{\varphi}$ along the eigenmodes. We use Supplementary Eq. 51 in Supplementary Eq. 46 to find the evolution equations for the amplitudes $\varphi^a(t)$. A direct substitution of Supplementary Eq. 51 in Supplementary Eq. 48 gives

$$mr^2 \sum_a \frac{\mathrm{d}^2\varphi^a}{\mathrm{d}t^2} e_a^i = -\sum_a \omega_a^2 \varphi^a e_a^i - \frac{1}{2}\sum_{b,c} \varphi^b \varphi^c V_{,ijk} e_b^j e_c^k. \tag{53}$$

where repeated spatial indices j,k are implicitly summed. Next, we use orthonormality of the (real) eigenbasis:

$$\mathbf{e}_a \cdot \mathbf{e}_b = \sum_{i=1}^{N} e_a^i e_b^i = \delta_{ab} \tag{54}$$

to find the equation for the amplitude $\varphi^a(t)$. We multiply Supplementary Eq. 53 by e_s^i and we sum over i (*i.e.*, we project the equation onto $\mathbf{e}_s$) and we find the dynamical equation for the amplitudes:

$$mr^2 \frac{d^2\varphi^a}{dt^2} = -\omega_a^2 \varphi^a - \frac{1}{2}\sum_{b,c} \varphi^b \varphi^c V_{,ijk} e_a^i e_b^j e_c^k. \tag{55}$$

We can define a set of parameters that we may call coupling constants

$$\hat{g}_{abc} \equiv V_{,ijk} e_a^i e_b^j e_c^k, \tag{56}$$

which represent the mutual couplings between the amplitudes φ^a. By construction, the couplings are symmetric under permutations of the indices a,b,c. The dynamical equation for the amplitudes can be rewritten as:

$$mr^2 \frac{d^2\varphi^a}{dt^2} = -\omega_a^2 \varphi^a - \frac{1}{2}\sum_{b,c} \hat{g}_{abc} \varphi^b \varphi^c. \tag{57}$$

As was done above with Eq. 49, we can also write Supplementary Eq. 57 as the following equivalent expression:

$$mr^2\frac{d^2\varphi^a}{dt^2} = -\omega_a^2\varphi^a - \frac{1}{6}\sum_{b,c,d} V_{,jkl}e_b^j e_c^k e_d^l \frac{\partial(\varphi^b\varphi^c\varphi^d)}{\partial\varphi^a}. \tag{58}$$

In going from Supplementary Eq. 57 to Supplementary Eq. 58 we used the following identity:

$$\begin{aligned}
V_{,ijk}e_s^i e_b^j e_c^k \frac{\partial(\varphi^s\varphi^b\varphi^c)}{\partial\varphi^a} &= V_{,ijk}e_s^i e_b^j e_c^k(\delta_{sa}\varphi^b\varphi^c + \varphi^s\delta_{ab}\varphi^c + \varphi^s\varphi^b\delta_{ca}) \\
&= V_{,ijk}[e_a^i e_b^j e_c^k\varphi^b\varphi^c + e_s^i e_a^j e_c^k\varphi^s\varphi^c + e_s^i e_b^j e_a^k\varphi^s\varphi^b] \\
&= V_{,ijk}[e_a^i e_b^j e_c^k + e_a^j e_b^i e_c^k + e_a^k e_b^j e_c^i]\varphi^b\varphi^c \\
&= (V_{,ijk} + V_{,jik} + V_{,kji})e_a^i e_b^j e_c^k\varphi^b\varphi^c \\
&= 3V_{,ijk}e_a^i e_b^j e_c^k\varphi^b\varphi^c,
\end{aligned} \tag{59}$$

where in the fourth line we changed the names of the summed indices and in the last line we used the symmetry of the partial derivatives. Finally, we notice that the coupling constants depend on $V_{,ijk}$, so we can express them in the coordinates $\boldsymbol{\varphi}$. By using Eq. (47) and Supplementary Eq. 52, we find the following expression for the coupling constants:

$$\begin{aligned}
\hat{g}_{abc} = V_{,ijk}e_a^i e_b^j e_c^k = \frac{\partial^3 V(\boldsymbol{\theta}_{\text{kink}})}{\partial\delta\theta^i\partial\delta\theta^j\partial\delta\theta^k}e_a^i e_b^j e_c^k &= \frac{\partial^3 V(\boldsymbol{\theta}_{\text{kink}})}{\partial\delta\theta^i\partial\delta\theta^j\partial\delta\theta^k}\frac{\partial\delta\theta^i}{\partial\varphi^a}\frac{\partial\delta\theta^j}{\partial\varphi^b}\frac{\partial\delta\theta^k}{\partial\varphi^c} \\
&= \frac{\partial^3 V(\boldsymbol{\theta}_{\text{kink}})}{\partial\varphi^a\partial\varphi^b\partial\varphi^c} \\
&= \left.\frac{\partial^3 V(\boldsymbol{\theta}_{\text{kink}} + \sum_d \varphi^d\mathbf{e}_d)}{\partial\varphi^a\partial\varphi^b\partial\varphi^c}\right|_{\boldsymbol{\varphi}=0}.
\end{aligned} \tag{60}$$

This completes the change of variables. By construction, the coupling constants depend on the choice of the initial configuration of the kink, since both $V_{,ijk}$ and the eigenvectors $\mathbf{e}_a$ are computed on the initial kink configuration $\boldsymbol{\theta}_{\text{kink}}$. The latter depends parametrically on the geometry of the unit cell $(\tilde{r}, \tilde{\bar{l}})$. By varying the geometry of the unit cell, one can tune the couplings between the modes' amplitudes and modify the dynamics of the excitations.

We are interested in the evolution of the system along the initial zero-energy translation mode $\mathbf{e}_0$, whose eigenfrequency is $\omega_0 = 0$. Choosing $a = 0$ in Supplementary Eq. 58 and using the second line of Supplementary Eq. 60, we find the equation of motion of the zero mode:

$$mr^2\frac{d^2\varphi^0}{dt^2} = -\frac{1}{6}\frac{\partial^3 V(\boldsymbol{\theta}_{\text{kink}})}{\partial\varphi^a\partial\varphi^b\partial\varphi^c}\frac{\partial(\varphi^a\varphi^b\varphi^c)}{\partial\varphi^0}. \tag{61}$$

Equation 61 contains the interaction of the zero mode with all the higher energy states, that include all internal modes and the modes within the pass band. In the simplest situation where there is a single internal mode φ^1 with isolated eigenfrequency ω_1 and using the expression of the coupling constants $\hat{g}_{abc}$, we can write Supplementary Eq. 61 more explicitly by isolating the terms that couple the zero mode to the internal mode $\mathbf{e}_1$:

$$mr^2\frac{d^2\varphi^0}{dt^2} = -\frac{1}{2}\Big(\hat{g}_{000}(\varphi^0)^2 + 2\hat{g}_{001}\varphi^0\varphi^1 + \hat{g}_{011}(\varphi^1)^2 + \sum_{\substack{b,c\geq 1\\ b\cdot c\neq 1}}\hat{g}_{0bc}\varphi^b\varphi^c\Big). \tag{62}$$

Similarly, setting $a = 1$ in 57 we obtain the evolution along the internal mode $\mathbf{e}_1$

$$mr^2\frac{d^2\varphi^1}{dt^2} = -\omega_1^2\varphi_1 - \frac{1}{2}\Big(\hat{g}_{100}(\varphi^0)^2 + 2\hat{g}_{101}\varphi^0\varphi^1 + \hat{g}_{111}(\varphi^1)^2 + \sum_{\substack{b,c\geq 1\\ b\cdot c\neq 1}}\hat{g}_{1bc}\varphi^b\varphi^c\Big). \tag{63}$$

In these equations the coupling constants depend on the unit cell parameters so the coupling between the modes (and so the dynamical response of the kink) can be tuned by changing the rotor length and the spring rest length.

This is how the dynamics is manipulated by the topological phase of the chain. It is therefore crucial to compute how the coupling constants depend on $(\tilde{r},\tilde{\tilde{l}})$. If there are $N_{\mathrm{int}} > 1$ internal modes, there will be N_{int} equations analogous to 63, where we extract from the sum the interactions of the zero mode with itself, of the zero mode with the internal modes, and of the interactions between the internal modes.

13.2 Numerical examination

To validate the mode coupling theory introduced above, we compare both sides of Supplementary Eq. 61 numerically, which are computed from the same time-domain simulation. In each case, we inject a small-amplitude acoustic wave packet with homogeneous mid-band frequencies from the end of a finite KL chain with N rotors, where N is even. An initial static kink is centered at the $(N/2)$th site ($\psi_{N/2} = 0$). We first introduce the method of examination, followed by a presentation of the numerical results.

To compute $\frac{d^2\varphi^0}{dt^2}$, we begin by determining the eigenmode $\mathbf{e}_0$ of the initial reference state, *i.e.*, the eigenvector corresponding to the zero mode of the chain at $t = 0$. By solving the Hamiltonian equation of motion (the solution consists of $\{\delta\theta^1(t),...,\delta\theta^N(t),p^1(t),...,p^N(t)\}$), we can directly extract the conjugate momentum p^i of each rotor (conjugate to $\delta\theta^i$) and record them as a momentum vector $\mathbf{p} = (p^1,...,p^N)$. The short-time measurement begins at t_1 when the $(N/2)$th rotor has rotated by $|\delta\theta^{N/2}| = \min\{0.01, 0.01\bar{\psi}\}$ from 0, and ends at t_2, when the $(N/2)$th rotor has rotated by $|\delta\theta^{N/2}| = \min\{0.02, 0.05\bar{\psi}\}$ from 0 (such choice of t_1 and t_2 is made to guarantee the validity of perturbation theory around the static kink). The time interval between t_1 and t_2 is assumed to represent the acoustic-wave–kink interaction time. From Supplementary Eq. 51, we have the relation between $\mathbf{p}$ and $\frac{d\varphi^0}{dt}$ as

$$\frac{\mathbf{p}\cdot\mathbf{e}_0}{mr^2} = \frac{d\boldsymbol{\delta\theta}\cdot\mathbf{e}_0}{dt} = \frac{d\varphi^0}{dt}. \tag{64}$$

By assuming linearity and performing a fit on the $\frac{d\varphi^0}{dt}$ data obtained from Supplementary Eq. 64 over the interval $[t_1, t_2]$, we have $\frac{d^2\varphi^0}{dt^2}$.

To compute $-\frac{1}{6mr^2}\frac{\partial^3 V}{\partial\varphi^i\partial\varphi^j\partial\varphi^k}\frac{\partial(\varphi^i\varphi^j\varphi^k)}{\partial\varphi^0}$, first, for each time t ($t > 0$ with $t = 0$ denoting the start of excitation and simulation), we record $\delta\theta^i(t)$ and then project $\boldsymbol{\delta\theta}(t)$ onto the eigenvectors of $D(t = 0)$ to obtain the amplitudes φ^i numerically. If we use Supplementary Eq. 61 of the potential, we have the equation of motion of φ^0 in the following form

$$\frac{d^2\varphi^0}{dt^2} = -\frac{1}{6mr^2}\left(\frac{\partial^3 V(\boldsymbol{\theta}_{\mathrm{kink}})}{\partial\varphi^0\partial\varphi^0\partial\varphi^0}\frac{\partial(\varphi^0\varphi^0\varphi^0)}{\partial\varphi^0} + 3\sum_{k>0}\frac{\partial^3 V(\boldsymbol{\theta}_{\mathrm{kink}})}{\partial\varphi^0\partial\varphi^0\partial\varphi^k}\frac{\partial(\varphi^0\varphi^0\varphi^k)}{\partial\varphi^0} + 3\sum_{j,k>0}\frac{\partial^3 V(\boldsymbol{\theta}_{\mathrm{kink}})}{\partial\varphi^0\partial\varphi^j\partial\varphi^k}\frac{\partial(\varphi^0\varphi^j\varphi^k)}{\partial\varphi^0}\right). \tag{65}$$

On the other hand, one could write the potential energy in a third-order symbolic form as

$$V(\boldsymbol{\varphi}) = \frac{1}{2}\sum_{a=0}^{N-1}\omega_a^2(\varphi^a)^2 + g_{000}\varphi^0\varphi^0\varphi^0 + \sum_{k>0} g_{00k}\varphi^0\varphi^0\varphi^k + \sum_{j,k>0} g_{0jk}\varphi^0\varphi^j\varphi^k + \sum_{i,j,k>0} g_{ijk}\varphi^i\varphi^j\varphi^k, \tag{66}$$

where g_{000}, g_{00k}, g_{0jk}, and g_{ijk} are the coefficients of expansion. With the Euler-Lagrange equations for φ^0

$$mr^2\frac{d^2\varphi^0}{dt^2} = -\frac{\partial V(\boldsymbol{\varphi})}{\partial\varphi^0}, \tag{67}$$

we have

$$\begin{aligned}\frac{d^2\varphi^0}{dt^2} &= -\frac{1}{mr^2}\frac{\partial V(\boldsymbol{\varphi})}{\partial\varphi^0}\\ &= -\frac{1}{mr^2}\left(3g_{000}\varphi^0\varphi^0 + 2\sum_{k>0} g_{00k}\varphi^0\varphi^k + \sum_{j,k>0} g_{0jk}\varphi^j\varphi^k\right)\\ &= -\frac{1}{mr^2}\left(\frac{3}{\varphi^0}g_{000}\varphi^0\varphi^0\varphi^0 + \frac{2}{\varphi^0}\sum_{k>0} g_{00k}\varphi^0\varphi^0\varphi^k + \frac{1}{\varphi^0}\sum_{j,k>0} g_{0jk}\varphi^0\varphi^j\varphi^k\right).\end{aligned} \tag{68}$$

Note that Supplementary Eq. 65 and 68 are equivalent. By evaluating the right side of 68, one can validate Supplementary Eq. 65, as well as Supplementary Eq. 61. To evaluate the right-hand side of Supplementary Eq. 68, we define an auxiliary function $G(u,v)$ in the following way:

$$G(u,v) = V(u\varphi^0, v\varphi^1 ..., v\varphi^{N-1}). \tag{69}$$

We note that all φ^a are numerical coefficients, determined from the time-domain simulations. We Taylor expand $G(u,v)$

$$\begin{aligned} G(u,v) = &\left(\frac{1}{2}\sum_{a=1}^{N-1}\omega_a^2(\varphi^a)^2\right)v^2 + \left(g_{000}\varphi^0\varphi^0\varphi^0\right)u^3 + \left(\sum_{k>0} g_{00k}\varphi^0\varphi^0\varphi^k\right)u^2 v \\ &+ \left(\sum_{j,k>0} g_{0jk}\varphi^0\varphi^j\varphi^k\right)uv^2 + \left(\sum_{i,j,k>0} g_{0jk}\varphi^i\varphi^j\varphi^k\right)v^3. \end{aligned} \tag{70}$$

Once all modes $\varphi^0, \varphi^1, ..., \varphi^{N-1}$ are computed, we multiply φ^0 by u, φ^j ($j > 0$) by v and plug them into the potential V. The resulting expression Supplementary Eq. 70 will be a polynomial with numerical coefficients and powers of u,v. One can find $g_{000}\varphi^0\varphi^0\varphi^0$ from the coefficient of u^3, $\sum_{k>0} g_{00k}\varphi^0\varphi^0\varphi^k$ from the coefficient of $u^2 v$ and $\sum_{j,k>0} g_{0jk}\varphi^0\varphi^j\varphi^k$ from the coefficient of uv^2. These expressions can be used to calculate the right-hand side of Supplementary Eq. 68 and verify the third-order perturbation theory.

We numerically examined kink states in the middle of the KL chain for $\tilde{r} = 0.8$, 1.5, and 2.0, with $N = 100$, $a = 1$ m, $k_e = 100$ N m^{-1}, and $m = 1$ kg. These states are indicated by the orange lines and markers in Supplementary Fig. 18**a**, which shows the equivalent phase diagram to Fig. 2**a**, but includes all the F, WF, and S phases. For the initial static kink solution, we still use the transfer and inverse transfer functions (Supplementary Eqs. 34, 35, 38, and 39) to solve both forward and backward from a rotor positioned in the middle of the chain. However, the method for selecting the appropriate solutions differs in this section. Specifically, we use a reference state obtained from a dynamical simulation to determine which solutions to select. The process is as follows: First, we generate a homogeneous chain configuration, compute the zero mode of the chain, assign an initial conjugate momentum $\mathbf{p}$ to each rotor that is proportional to the amplitude of the normalized zero mode $\mathbf{v}_0$, and then simulate the chain's dynamics. In this simulation, the kink is generated from the soft end and propagates through the chain. Second, we record the chain configuration at the time t_0, when the rotor in the middle of the chain reaches the designated angle, along with the angles of all other rotors in the chain at this time. Third, we solve the chain configuration starting from the rotor in the middle of the chain using the transfer and inverse transfer functions, selecting the solutions that most closely match the rotor angles from the recorded chain configuration at time t_0. By repeating this process, we can generate a static chain configuration with a kink positioned in the middle. For the case indicated by circle and square markers in Supplementary Fig. 18**b**, ψ_{50} is set to 0 and 0.1 rad, respectively. For the cases indicated by the triangles and error bars in Supplementary Fig. 18**b**, which correspond to kink states positioned between two sites (the 50th and 51st sites), we record the time t_0 when $\psi_{50} = 0$ in the dynamical simulation, and a nearby time t_1 when $\psi_{51} = 0$. We then divide the time interval $[t_0, t_1]$ evenly into 10 sub-intervals, and generate the chain configurations based on ψ_{51} at times $t = t_0 + 0.1(t_1 - t_0)$, $t_0 + 0.2(t_1 - t_0)$, $\ldots$, t_1. Acoustic waves are injected into the chain through a torque applied to the first rotor, described by $\tau(t) = e^{-(t-t_0)^2/(2\sigma_t^2)}\cos\omega t$, where $\sigma_t = 12\pi/\omega$, $t_0 = 40\pi/\omega$, and ω represents the homogeneous mid-band frequency of the corresponding chain. The simulation runs for a duration of 2000 s. The sampling rate is optimally determined using adaptive methods via the *NDSolve* function in Mathematica.

Supplementary Fig. 18**b** summarizes the results of the numerical comparison between the simulated short-time kink acceleration $d^2\varphi^0/dt^2$ (blue) and the kink acceleration based on the third-order perturbation theory (*i.e.*, the right side of Supplementary Eq. 68, red) while acoustic wave packets are injected from one rigid end of the chain. A good agreement is observed between these two terms. The results reveal that acoustic wave packets can either attract or repel kinks in the KL chain, as indicated by the negative and positive acceleration values, respectively. (Although kink attraction via acoustic wave interaction is the more commonly observed phenomenon[8,11,12], computational studies have also demonstrated cases of kink repulsion in discrete ϕ^4[13] and ϕ^6[12] chains.) Strong repulsion is seen particularly in the WF phase for $\tilde{r} = 0.8$. For $\tilde{r} = 1.5$ and 2.0, we observe large negative accelerations across the WF phase regime, except near the boundaries with the F phase. In the F phase, large negative accelerations are only observed close to the F-WF phase boundary. Although we also observe negative accelerations in the S phase for $\tilde{r} = 1.5$ and 2.0, they are small compared to those in the F and WF phases. Notably, for $\tilde{r} = 1.5$, the negative acceleration increases as d approaches its upper limit.

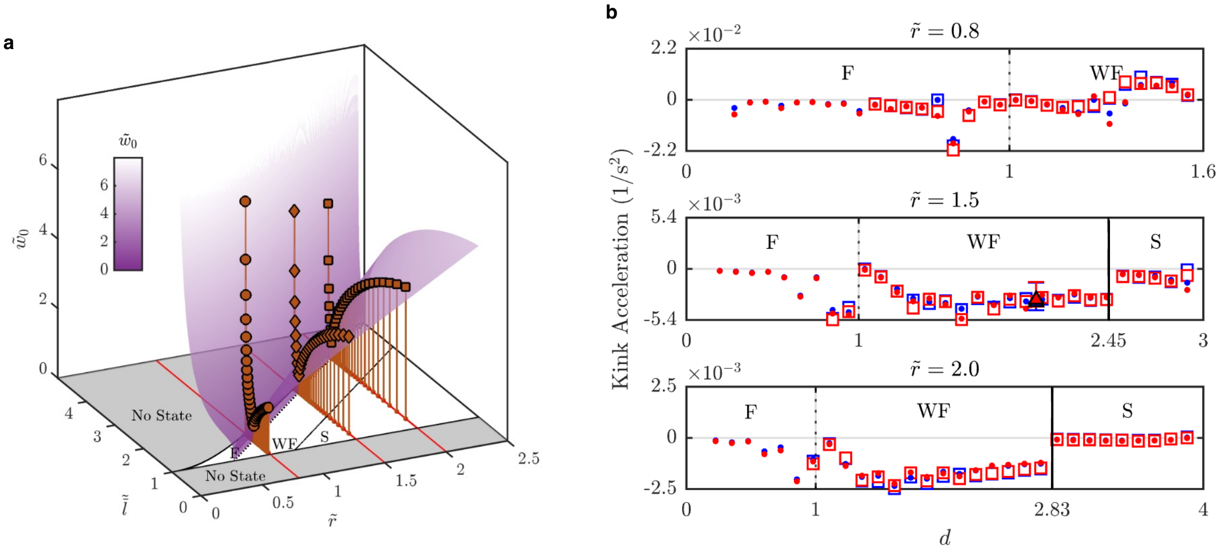

Supplementary Figure 18. Short-time acoustic-wave-induced kink accelerations. In the simulation, the wavepacket is injected from the left end of the KL chain, so negative acceleration indicates initial attraction towards the wave packet. **a** Phase diagram of zero-energy kink states in the KL chain, with kink widths shown for all three phases. Red lines indicate $\tilde{r} = 0.8$, 1.5, and 2.0, and orange markers correspond to the examined states. **b** The circle and square markers represent kink states centered at the 50th site ($\psi_{50} = 0$) and slightly off-center ($\psi_{50} = 0.1$ rad), respectively. Positive [Negative] values indicate the kink moves away [towards] the source of the acoustic waves, *i.e.*, repulsion [attraction]. Triangle markers in the middle panel represent the mean accelerations for the kink configuration with experimental unit cell geometry as it moves between the 50th and 51st sites, with error bars indicating the maximum and minimum values.

Supplementary Note 14. NUMERICAL INVESTIGATION OF DIFFERENT ACOUSTIC-WAVE–KINK INTERACTION REGIMES FOR THE ZERO-ENERGY KINKS IN THE KL CHAIN

We numerically investigated the types of acoustic-wave–kink interactions in KL chains with different unit cell geometries using short-time dynamic simulations. As in the previous section, the parameters were set as $a = 1$ m, $k_e = 100$ N m^{-1}, $m = 1$ kg, and $N = 100$, with the kink centered at rotor $N/2 = 50$ ($\psi_{50} = 0$). Acoustic waves were injected through the same torque applied to the first rotor as described in the previous section. Each simulation was run for a total duration of $t_0 + Na/v_g$, where v_g is the group velocity of acoustic waves in the corresponding homogeneous chain. The simulations were carried out in MATLAB using the *ode45* solver, with 'RelTol' and 'AbsTol' both set to 10^{-6}. By linearly fitting the angular velocity data of rotor 50 ($\dot{\psi}_{50}$) between t_1 and t_2 (defined in the previous section), we obtained the corresponding kink accelerations, which are overlaid on the geometric phase diagram, as shown in Supplementary Fig. 19.

For most unit cell geometries across all three phases, the acoustic-wave–kink interaction exhibits an attraction-type behavior (negative acceleration), which tends to become stronger as $\tilde{r}$ and $\tilde{l}$ decrease. Along the lower boundary of the diagram, however, the interaction transitions to a repulsive type (positive acceleration) and gradually weakens toward the WF-S phase boundary. The transition between the two interaction regimes does not appear smooth, and the boundary separating them is clearly distinguishable. A weakly repulsive case is also observed in the WF phase, away from the boundary.

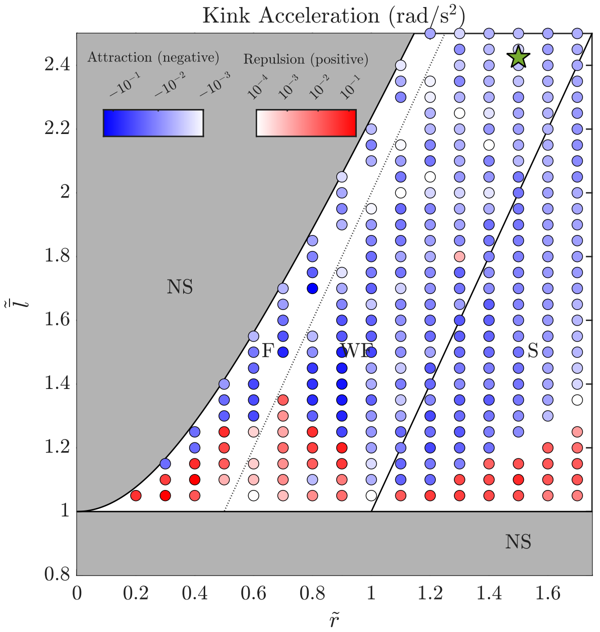

Supplementary Figure 19. Acoustic-wave–kink interaction types in KL chains with different unit cell geometries from short-time dynamic simulations. Red and blue colors indicate fitted kink acceleration (negative: attraction, positive: repulsion). Green star indicates the experimental kink state.

Supplementary Note 15. EFFECTIVE SPRING

One challenge in constructing the KL chain is obtaining physical springs that can linearly stretch and compress along the normal direction within the desired strain range, as typical coil springs exhibit linear behavior under tension but are prone to buckling under compression. Additionally, tuning the spring stiffness while maintaining the rest length is also challenging. To address these issues, we use a pre-buckled beam-based structure as our spring, as shown in Supplementary Fig. 20**a**, allowing us to select the effective normal stiffness (Supplementary Fig. 20**b**) based on its geometry.

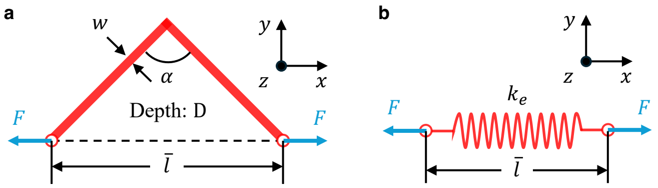

Supplementary Figure 20. Schematics of pre-buckled beam-based structure **a** and its effective spring model **b**. Circular ends represent reaction-moment-free boundary conditions. F indicates normal force.

15.1 Beam Theory

Here we derive the effective normal stiffness of the beam-based pre-buckled spring as shown in Supplementary Fig. 20**a**, using beam theory and energy methods.

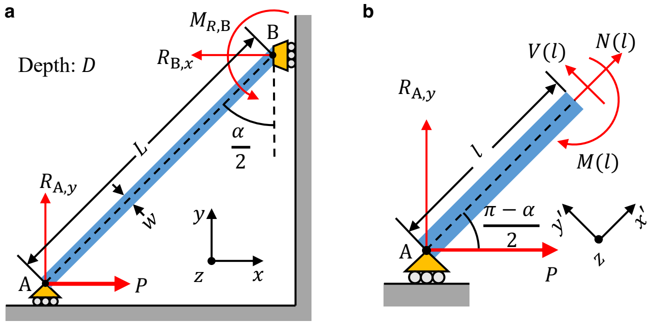

Supplementary Figure 21. **a** Free-body diagram of a long, thin beam model (in blue, with a rectangular cross section shape) of length L, width w, depth D (in the z direction), and angle $\alpha/2$ relative to the vertical line, with a pin/roller boundary condition applied at the end A in the x direction and a roller boundary condition at the end B in the y direction. A horizontal load P is applied at the point A. **b** Cut diagrams of both sides from the cut point. Axes x' and y' indicate the axial and shear directions of the beam, and l represents the distance from the point A along x' direction.

Consider the long, thin ($w/L \ll 1$) beam model in Supplementary Fig. 21**a** as a half of the pre-buckled spring. The equilibrium equations of static equilibrium can be written as

$$\sum F_x = P - R_{B,x} = 0, \tag{71}$$

$$\sum F_y = R_{A,y} = 0, \tag{72}$$

$$\sum M_A = M_{R,B} + R_{B,x} L \cos\left(\frac{\alpha}{2}\right) = 0, \tag{73}$$

where the geometric and force and moment variables are defined as in Supplementary Fig. 21. From Supplementary Eq. 71, we find

$$R_{B,x} = P, \tag{74}$$

and substituting Supplementary Eq. 74 in Supplementary Eq. 73, we find

$$M_{R,\mathrm{B}} = -PL\cos(\frac{\alpha}{2}). \tag{75}$$

From this point, we will use the Castigliano's theorem[14]. We first apply a cut as shown in Supplementary Fig. 21**b**. Based on Supplementary Fig. 21**b**, we write the equations of translational static equilibrium in the x' and y' directions as

$$\sum F_{x'} = P\sin(\frac{\alpha}{2}) + N(l) = 0 \tag{76}$$

and

$$\sum F_{y'} = -P\cos(\frac{\alpha}{2}) + V(l) = 0, \tag{77}$$

where $N(l)$ and $V(l)$ are the normal and shear forces at the cut point, at a distance of l from the point A along the beam. From Supplementary Eqs. 76 and 77, we find

$$N(l) = -P\sin(\frac{\alpha}{2}) \tag{78}$$

and

$$V(l) = P\cos(\frac{\alpha}{2}). \tag{79}$$

We write the static equation of moment equilibrium at the cutting point as

$$\sum M_{x'=l} = -M(l) + Pl\cos(\frac{\alpha}{2}) - R_{\mathrm{A},y}l\sin(\frac{\alpha}{2}) = 0. \tag{80}$$

Substituting Supplementary Eq. 72 in Supplementary Eq. 80, we find

$$M(l) = Pl\cos(\frac{\alpha}{2}). \tag{81}$$

Since the strain energy stored in the beam due to the action of axial force (U_a), bending moment (U_b), direct shear force (U_s), and transverse shear force (U_ts) are given by

$$U_\mathrm{a} = \int_0^L \frac{N(l)^2}{2AE}dl, \tag{82}$$

$$U_\mathrm{b} = \int_0^L \frac{M(l)^2}{2EI_A}dl, \tag{83}$$

$$U_\mathrm{s} = \int_0^L \frac{V(l)^2}{2AG}dl, \tag{84}$$

and $U_\mathrm{ts} = c_\mathrm{ts}U_\mathrm{s}$, where A and I_A are the cross section area and the area moment of inertia of the beam, respectively, E and G are the elastic and shear modulus of the material, respectively, and c_ts is the form factor, which is dependent on the shape of the cross section. The total strain energy reads $U = U_\mathrm{a} + U_\mathrm{b} + U_\mathrm{s} + U_\mathrm{ts}$.

Since we apply a horizontal load P at the point A, as shown in Supplementary Fig. 21, the effective stiffness in the x direction, $k_{\mathrm{eff},x}$, can be defined as P/δ_x, where δ_x is the total deflection in the x direction, which is the sum of axial (δ_a), bending (δ_b), direct shear (δ_s), and transverse shear deflections (δ_ts). To find out each deflection, we use Castigliano's second theorem for each strain energy, leading to

$$\delta_\mathrm{a} = \frac{\partial U_\mathrm{a}}{\partial P} = \frac{LP\sin^2(\alpha/2)}{AE}, \tag{85}$$

$$\delta_b = \frac{\partial U_b}{\partial P} = \frac{L^3 P \cos^2(\alpha/2)}{3EI_A}, \tag{86}$$

$$\delta_s = \frac{\partial U_s}{\partial P} = \frac{LP\cos^2(\alpha/2)}{AG}, \tag{87}$$

and $\delta_{ts} = c_{ts}\delta_s$. By substituting $A = Dw$, $I_A = Dw^3/12$, and $G = E/(2(1+\nu))$, where ν is the Poisson's ratio, into Supplementary Eqs. 85, 86, and 87, we obtain

$$\delta_a = \left(\frac{P\sin^2(\alpha/2)}{ED}\right)\left(\frac{L}{w}\right), \tag{88}$$

$$\delta_b = \left(\frac{4P\cos^2(\alpha/2)}{ED}\right)\left(\frac{L}{w}\right)^3, \tag{89}$$

$$\delta_s = \left(\frac{2P(1+\nu)\cos^2(\alpha/2)}{ED}\right)\left(\frac{L}{w}\right). \tag{90}$$

Since we consider $w/L \ll 1$, we then have

$$\frac{\delta_s}{\delta_b} = \frac{1}{2}(1+\nu)\left(\frac{w}{L}\right)^2 \to 0, \tag{91}$$

and $\delta_{ts}/\delta_b \to 0$, leading us to ignore the direct and transverse shear deflections in the total deflection. The ratio between axial and bending deflections reads

$$\frac{\delta_a}{\delta_b} = \frac{1}{4}\left(\frac{w}{L}\right)^2 \tan^2(\frac{\alpha}{2}), \tag{92}$$

which depends the choice of α besides w/L, leading us to consider keeping both axial and bending deflections in the total deflection

$$\delta_x = \delta_a + \delta_b. \tag{93}$$

By using Supplementary Eqs. 88, 89, and 93, the effective stiffness in the x direction can then be written as

$$k_{\text{eff},x} = \frac{P}{\delta_x} = \frac{ED}{4\gamma^3(1+\cos\alpha)+\gamma(1-\cos\alpha)}, \tag{94}$$

where $\gamma = L/w \gg 1$. Therefore, the effective normal stiffness k_e of the pre-buckled beam-based structure, as shown in Supplementary Fig. 20**b**, can be written as

$$k_e = \frac{1}{2}k_{\text{eff},x} = \frac{ED}{4\gamma^3(1+\cos\alpha)+\gamma(1-\cos\alpha)}, \tag{95}$$

where γ needs to satisfy $\gamma = \bar{l}\csc(\alpha/2)/(2w) \gg 1$.

15.2 Physical polycarbonate spring

The dimensions of physical spring are shown in Supplementary Fig. 22**a**. We chose parameters $\alpha = 60°$, $w = 2.6$ mm, $D = 5.95$ mm, and $\bar{l} = 48.5$ mm. The material was chosen to be polycarbonate due to its excellent ductility. From Supplementary Eq. 95, we predicted the effective normal stiffness as 401.6 N m^{-1}, by using a Young's modulus of $E = 2.1\times10^9$ N m^{-2} (similar to polycarbonate) and $\bar{l} = 48.5 - 2r_p\sin(\alpha/2) = 45.0$ mm ($r_p = 3.5$ mm is the inner radius of the pocket), indicated by the green dashed line, in order to correct the extra length created by the hollow cavities (pockets) at both ends. The springs were fabricated from 5.95 mm thick polycarbonate sheets via a desktop

computer-numerical-control (CNC) mill (Carbide 3D Nomad 3) with a bit size of 1/8 in (3.175 mm). A fabricated spring is shown in Supplementary Fig. 22**b**.

To get the effective normal stiffness of the polycarbonate spring, we used a universal testing machine (MTS Exceed Model E34) to measure the force versus displacement relation along the normal direction, as shown in Supplementary Fig. 22**c**. Small-sized ball bearings were press fit and glued into the pockets at the ends of the spring. Centers of ball bearings were aligned vertically and pinned to two laser cut acrylic plates (5.73 mm thick), which were clamped by the testing machine. A tension test was performed first by prescribing the displacement starting from the rest length of the polycarbonate spring ($\bar{l} = 48.5$ mm) at a displacement rate of 0.05 mm/s with a sample rate of 50 Hz. Once the tension test completed, the testing machine returned the spring to its unstretched length. A compression test was then performed at the same displacement rate with a sample rate of 10 Hz. The tension and compression test results are plotted together in Supplementary Fig. 22**d**, where the linear fitting slope indicates the effective normal stiffness is 384.3 N m^{-1} within ±10% strain range, close to theoretical prediction.

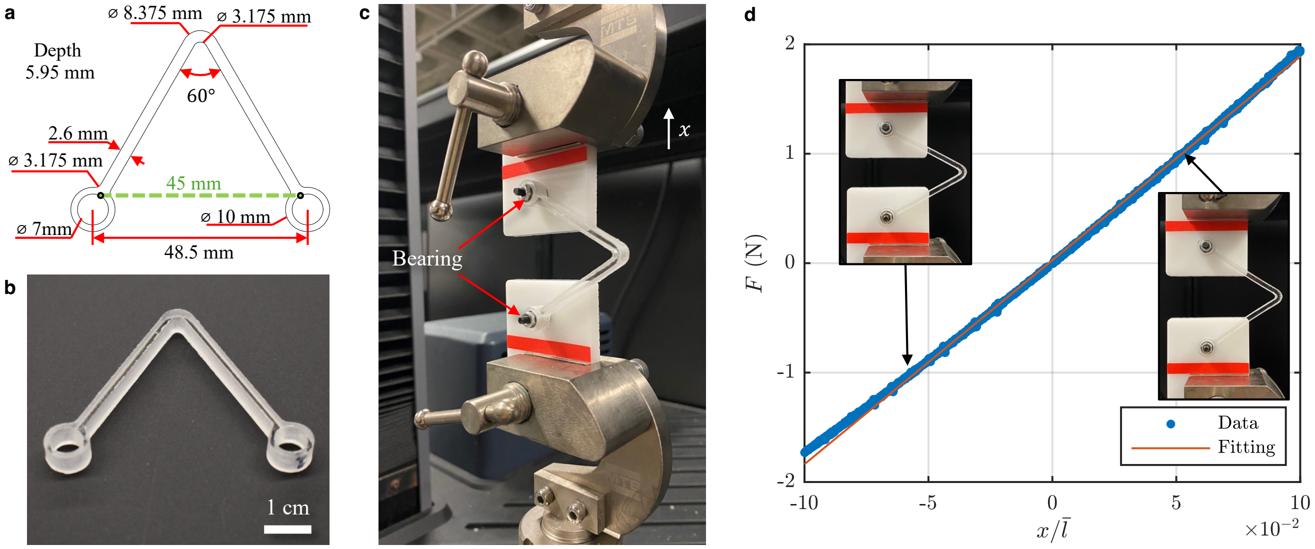

Supplementary Figure 22. Polycarbonate spring characterization results. **a** Dimensions of the physical spring. Fillets are added due to the limit of fabrication. Green dashed line indicates the length used to estimate theoretical normal stiffness. **b** A CNC-machined polycarbonate spring. **c** Experimental setup for measuring the force versus displacement relation of the pre-buckled polycarbonate spring in the normal direction. **d** Measured force versus strain results. Inset photos show the spring under compression and tension.

We also measured the force versus displacement relation of a steel coil spring with a rest length of 36 mm to demonstrate the buckling problem. The setup is shown in Supplementary Fig. 23**a**. Small-sized ball bearings were press fit and glued into the pockets at the ends of the spring. Centers of ball bearings were aligned vertically and pinned to two polycarbonate pieces that were glued to the test machine. Data was collected by pre-stretching the steel coil spring up to about 8% strain then compressing it, at a displacement rate of 0.05 mm/s with a sample rate of 10 Hz, till passing −10% strain. In Supplementary Fig. 23**b**, in spite of surprisingly well linear behavior under tension, the coil spring suffers from buckling starting from around 2.5% strain under compression. Fitted stiffness in the non-buckling regime (non-shaded area) is about 338 N m^{-1}.

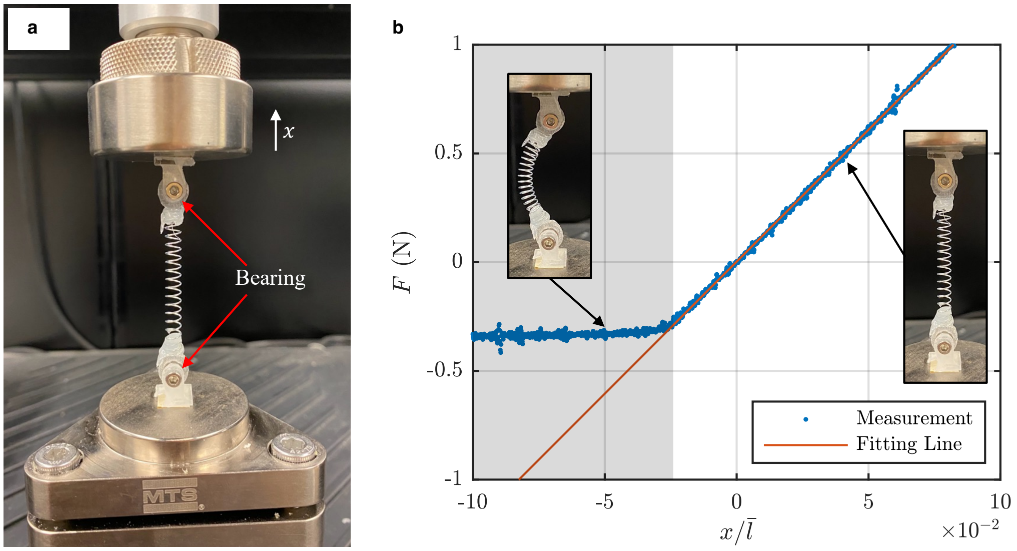

Supplementary Figure 23. Steel coil spring characterization results. **a** Experimental setup for measuring the force versus displacement relation of the steel coil spring in the normal direction. **b** Measured force versus strain results. Linear fitting line is based on data in the non-shaded area. inset photos depict the buckling and stretching states of the spring under compression and tension, respectively.

Supplementary Note 16. ROTOR

The dimensions of the rotor discs are shown in Supplementary Fig. 24**a**. The rotor discs were fabricated from acrylic sheets (2.8 mm thick) using a desktop laser cutter (Glowforge 3D laser printer). The top disc is opaque white, while the bottom disc is transparent. The flange components bolted to the center of the disc was fabricated from polycarbonate sheets (12.7 mm thick) using a desktop CNC mill (Carbide 3D Nomad 3). The weight of the disc was evenly distributed using nuts and bolts, as shown in Supplementary Fig. 24**b**. The top and bottom discs were assembled with the flange components, which are connected through the inner ring of the central steel bearing. These flange components were aligned via a male-to-female connector design, allowing the top and bottom parts of the rotor to rotate together. A pole was installed vertically to the disc at a radius of 30 mm with a small-sized steel bearing (7 mm outer diameter) installed at its end. The horizontal rung was laser cut from acrylic sheets.

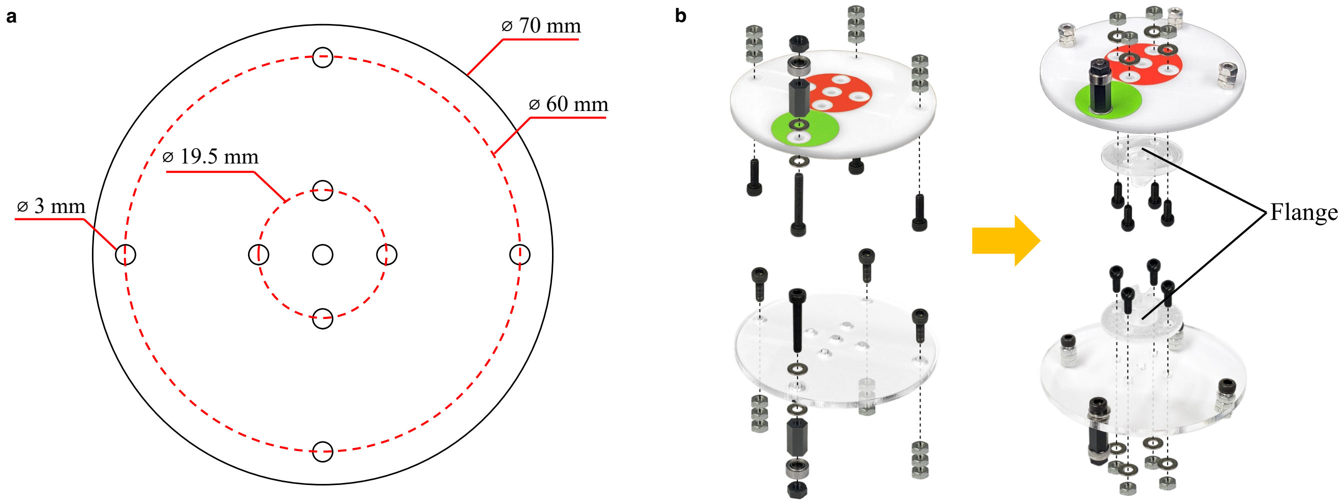

Supplementary Figure 24. **a** Dimensions of the rotor discs. **b** Top and bottom discs with even distributed mass and flanges assembled.

Supplementary Note 17. DETAILS ON EXCITATION FUNCTIONS

17.1 Kink control with acoustic wave packets

For the cases of kink control where the kink is positioned in the middle of the chain, the experimental excitation voltage signal to shaker 1 (via amplifier) follows a Gaussian-modulated sinusoidal function

$$V(t) = V_0 e^{-\frac{(t-T/2)^2}{2\sigma_t^2}} \sin 2\pi f t \quad 0 \le t \le T, \tag{96}$$

where V_0 is the amplitude of the excitation signal, $T = N_{\text{cycle}}/f$, $\sigma_t = 0.15T$, $N_{\text{cycle}} = 40$, and $f = 15.65$ Hz. The gain of shaker 1 was set to 10 dB.

In the corresponding simulations, the driving torque follows the same format of Supplementary Eq. 96, expressed as

$$\tau(t) = \tau_0 e^{-\frac{(t-T/2)^2}{2\sigma_t^2}} \sin 2\pi f t \quad 0 \le t \le T. \tag{97}$$

17.2 Kink generation with acoustic wave packets

For the case of kink generation in a homogeneous chain, the experimental excitation voltage signal to shaker 1 (via amplifier) follows

$$V(t) = \begin{cases} V_0 e^{-\frac{(t-T/4)^2}{2\sigma_t^2}} \sin 2\pi f t, & 0 \le t \le \frac{T}{4} \\ V_0 \sin 2\pi f t, & \frac{T}{4} < t \le \frac{3T}{4} \\ V_0 e^{-\frac{(t-3T/4)^2}{2\sigma_t^2}} \sin 2\pi f t, & \frac{3T}{4} < t \le T \end{cases} \tag{98}$$

where $T = N_{\text{cycle}}/f$, $N_{\text{cycle}} = 110$, $\sigma_t = 0.075T$, and $f = 12.7$ Hz. The gain of shaker 1 was set to 10 dB.

In the corresponding simulations, the driving torque follows the same format of Supplementary Eq. 98, expressed as

$$\tau(t) = \begin{cases} \tau_0 e^{-\frac{(t-T/4)^2}{2\sigma_t^2}} \sin 2\pi f t, & 0 \le t \le \frac{T}{4} \\ \tau_0 \sin 2\pi f t, & \frac{T}{4} < t \le \frac{3T}{4} \\ \tau_0 e^{-\frac{(t-3T/4)^2}{2\sigma_t^2}} \sin 2\pi f t, & \frac{3T}{4} < t \le T. \end{cases} \tag{99}$$

The normalized excitation function is plotted in Supplementary Fig. 25.

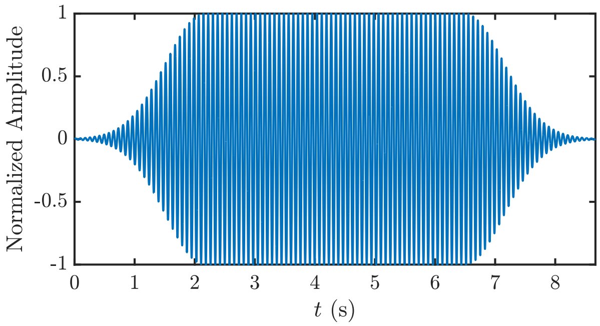

Supplementary Figure 25. Normalized excitation function used in the kink generation case.

Supplementary Note 18. EXPERIMENTAL SETUP AND DATA ACQUISITION

Shaker 1, an electrodynamic exciter (The Modal Shop, Smartshaker K2007E01), performed linear actuation and was driven by a signal generator (Tektronix AFG3022C) controlled via MATLAB. The signal generator was triggered by a square pulse from a second signal generator. In the kink control cases, the same trigger was also sent to a flash, which appeared in all videos for synchronization. In the kink generation case, the trigger was additionally sent to Shaker 2 (same model as Shaker 1), which produced a high-frequency reference audio signal (5000 Hz) used for video synchronization.

For data collection, each iPhone captured the response of 6 rotors, with the tilt angle adjusted to prevent overlap of the white top discs in the video. Recording was manually started on each iPhone before excitation began and manually stopped afterward. We extracted and processed data from the synchronized slow-motion videos using MATLAB. To synchronize the videos in the kink control cases, each video frame was summed in grayscale, and the resulting temporal intensity peak, corresponding to the flash, was identified. The excitation began 10 s after the flash trigger. For the kink generation case, we extracted the audio tracks from all three videos and set $t = 0$ at the start of the audio reference signal (provided by Shaker 2), which coincided with the shaker's start.

After synchronization, we applied projective transformations to correct the tilt angle of each rotor in every frame. To achieve this, we cropped the image to isolate each rotor along with its corresponding blue markers on both sides. The corners of the blue markers (left corners for the left marker and right corners for the right marker), indicated by points A, B, C, and D in the Before column of Supplementary Fig. 26, were used as the control points for the transformation. The length-to-width ratio of the chosen area is 163 mm/40 mm. Using this ratio and MATLAB functions *cp2tform*, *imtransform*, and *tformfwd*, we performed projective transformations from the Before to the After column in Supplementary Fig. 26. After the transformation, the rotors in their corresponding images appears as though viewed from directly above.

To track the rotor angles after the projective transformation, the red (R), green (G), and blue (B) channels of the frame were separated to create a monochrome image using the *imsplit* function in MATLAB. The red intensity was then enhanced using $\mathbf{I}_{\text{R, extra}} = \max(\mathbf{I}_{\text{R}} - \max(\mathbf{I}_{\text{G}}, \mathbf{I}_{\text{B}}), 0)$, and similarly, the green intensity was enhanced using $\mathbf{I}_{\text{G, extra}}$ using $\mathbf{I}_{\text{G, extra}} = \max(\mathbf{I}_{\text{G}} - \max(\mathbf{I}_{\text{R}}, \mathbf{I}_{\text{B}}), 0)$, where $\mathbf{I}_{\text{R}}$, $\mathbf{I}_{\text{G}}$, and $\mathbf{I}_{\text{B}}$ are the matrices corresponding to the red, green, and blue channels after splitting. The resulting $\mathbf{I}_{\text{R, extra}}$ and $\mathbf{I}_{\text{G, extra}}$ images were converted into binary images using thresholds, ensuring that only the markers appeared as white. The centers of the red and green markers were determined by averaging the coordinates of the white regions in each channel. To correct for minor frame rate differences between the iPhones (*e.g.*, 239.49 fps vs. 240.37 fps), the position tracking results were then interpolated to a uniform 240 fps. A 5-frame moving average was applied using MATLAB's *smoothdata* function to reduce noise, and rotor angles were calculated from the smoothed positions. For characterizing the rotor's onsite damping, circular markers were tracked using MATLAB's *imfindcircles* function, as shown in Supplementary Fig. 28**a**.

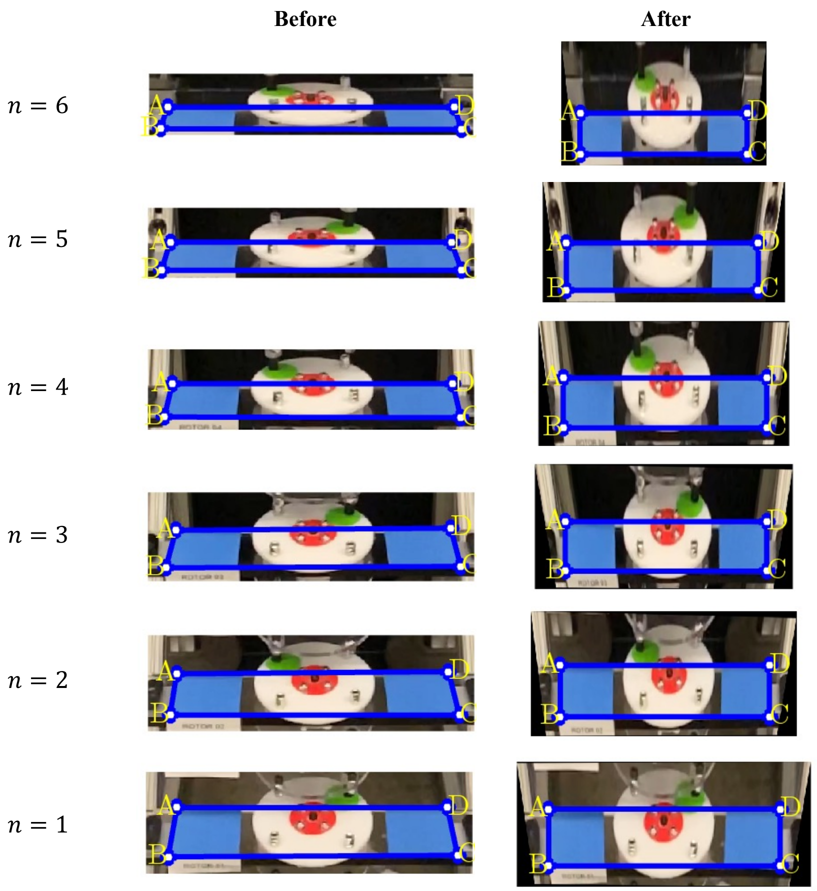

Supplementary Figure 26. Cropped rotor images from a single video frame for rotors 1–6, showing the states before and after projective transformation.

Supplementary Note 19. SIMULATION OF A LONGER CHAIN WITH EXPERIMENTAL UNIT CELL PARAMETERS AND NO DAMPING

To show that the stop of the kink's movement observed in the experiment is due to dissipation, we examine the kink dynamics triggered by acoustic waves in a longer KL chain with the same unit cell parameters as the experiment, consisting of $N = 400$ rotors, and without onsite damping. The kink is initially placed on the 200th site ($\psi_{200} = 0$). The first rotor is applied with a Gaussian-modulated sinusoidal torque, following $\tau(t) = \tau_0 e^{-(t-T/2)^2/(2\sigma_t^2)} \sin 2\pi f t$, where $\tau_0 = 4$ mN m, $T = N_{\mathrm{cycle}}/f$, $N_{\mathrm{cycle}} = 40$, $f = 15.65$ Hz, and $\sigma_t = 0.15T$. In Supplementary Fig. 27, we observe that the kink continues to move at a constant velocity even after the waves have moved away from it.

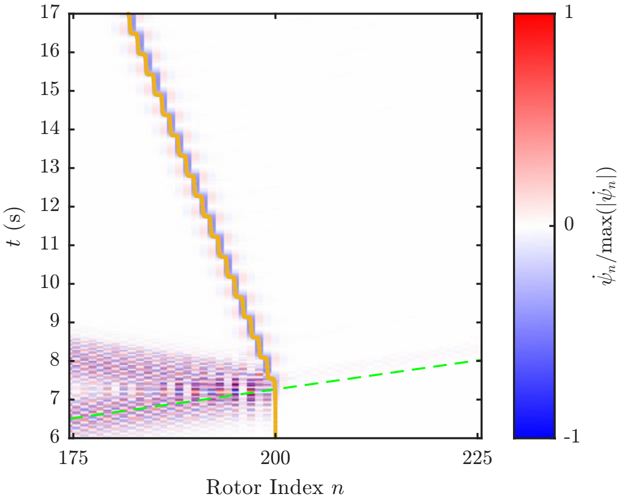

Supplementary Figure 27. Zoomed-in simulated spatiotemporal response in terms of normalized angular velocity of the KL chain, consisting of 400 rotors, without onsite damping. Green dashed line denotes the predicted wave packet center position based on the group velocity at the excitation frequency in the infinite homogeneous chain and the yellow solid line indicates the fitted kink center position.

Supplementary Note 20. ONSITE DAMPING CHARACTERIZATION

The onsite viscous damping coefficient c was calculated from the Q factor of a single rotor with the polycarbonate spring, which was experimentally determined by measuring the power spectrum from a free oscillation test and numerically fitting a Lorentzian function to it. We characterized the onsite damping using the top rotor in the chain. During the test, the top rotor was displaced from its equilibrium position by a certain angle and then released, while the neighboring rotor was held steady manually. A slow-motion video was recorded from above at 240 fps using an iPhone. Supplementary Fig. 28**a** shows an example of the tracking process. Supplementary Fig. 28**b** shows the result of tracking the angle changes $\Delta\theta$. The normalized power spectrum was obtained by squaring the fast Fourier transform (FFT) of the time-domain data (non-shaded area in Supplementary Fig. 28**b**) using the *fft* command in MATLAB. The $|\mathrm{FFT}(\Delta\theta)|^2$ data (Supplementary Fig. 28**c**) was fit to a Lorentzian function of the form

$$L(f) = \frac{A}{\pi} \frac{\Gamma/2}{(f-f_0)^2 + (\Gamma/2)^2}, \tag{100}$$

where A is an amplitude parameter, f_0 is the center frequency, and Γ is the full width at half max. The fitting was also done using the *fit* command in MATLAB, which gives $\Gamma = 0.6838$ Hz and $f_0 = 14.2$ Hz. The Q factor was found by taking f_0/Γ. The resultant Q factor was 20.8 giving a damping ratio $\zeta = 1/(2Q) = 0.024$. The damping coefficient was achieved by using $c_{\mathrm{exp}} = c_c\zeta$, where $c_c = 2I_{\mathrm{rotor}}(2\pi f_0)$ is the critical damping coefficient, leading to $c_{\mathrm{exp}} = 1.274 \times 10^{-4}$ N m s rad^{-1}.

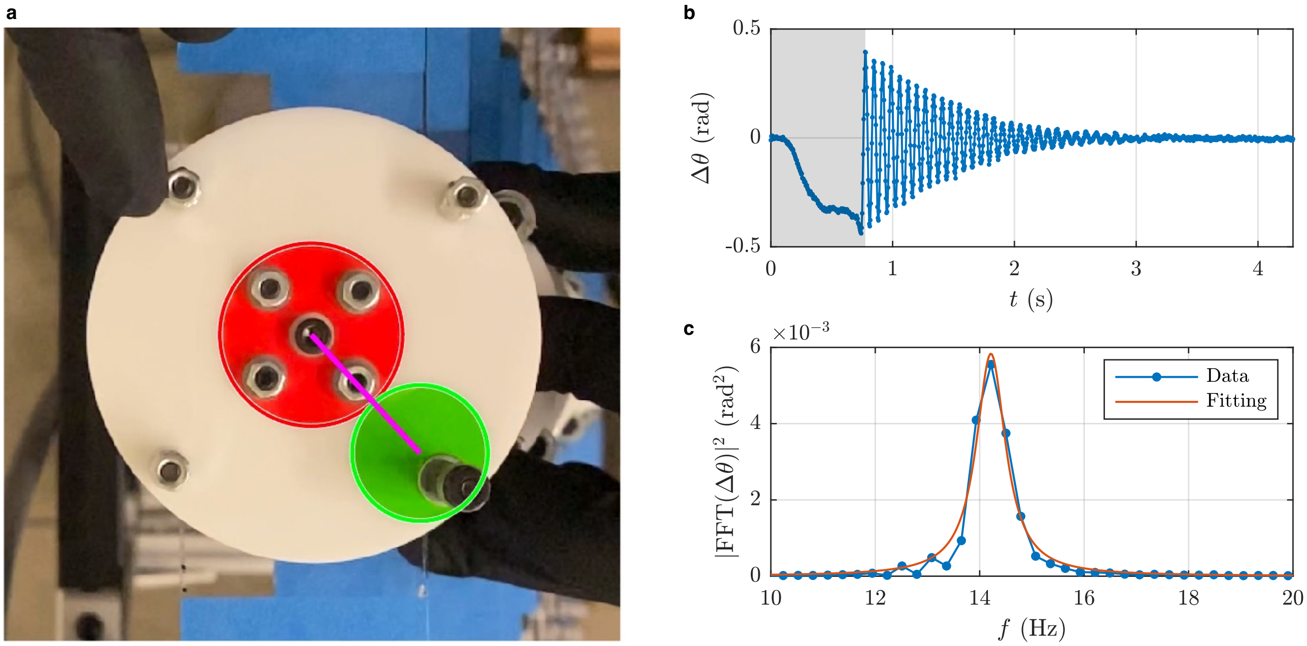

Supplementary Figure 28. Results of onsite damping characterization. **a** A cropped rotor image from a video frame for tracking rotor angle. Red and green circles indicate tracked markers and magenta solid line shows the rotor's direction. **b** Oscillation of the top rotor relative to its equilibrium position. **c** Zoomed-in power spectrum with Lorentzian function fitting.

Supplementary Note 21. MODE DECOMPOSITION FOR SHORT-TIME ACOUSTIC-WAVE–KINK INTERACTION IN KINK CONTROL

To study the short-time energy distribution across eigenmodes during the acoustic-wave–kink interaction, we projected the experimental and simulated displacement results of the kink control case shown in Fig. 5**g** and **h** onto the eigenvector basis of the initial chain configuration (with a kink onsite-centered at the middle of the chain), obtained using the Newton–Raphson method[4]. The results are shown in Supplementary Fig. 29, with time windows shifted between experimental and simulated plots for visual alignment. Mode 1 corresponds to the zero mode, while Mode 2 (10.41 Hz) and Mode 3 (10.48 Hz) correspond to the internal modes (indicated by the red box). The excitation frequency (15.65 Hz) is between the frequencies of Mode 9 (15.08 Hz) and Mode 10 (16.21 Hz).

We observe that, in both experiment and its corresponding simulation, the energy initially distributes into the zero mode and then rapidly transfers to the internal modes and the other modes within the pass band. We note that the time window is slightly different between the experiment and simulation cases (Fig. S40**a** and **b**). This shifted window shown was chosen to center the time when the modal weight of the first mode (the zero mode) begins increasing in amplitude. We suggest that this difference in zero mode excitation time between experiment and simulation may be due to differences in group velocity between reality and our model. In our results, we note that the excitation and the interaction predominantly involve odd-index modes rather than even-index ones within the pass band.

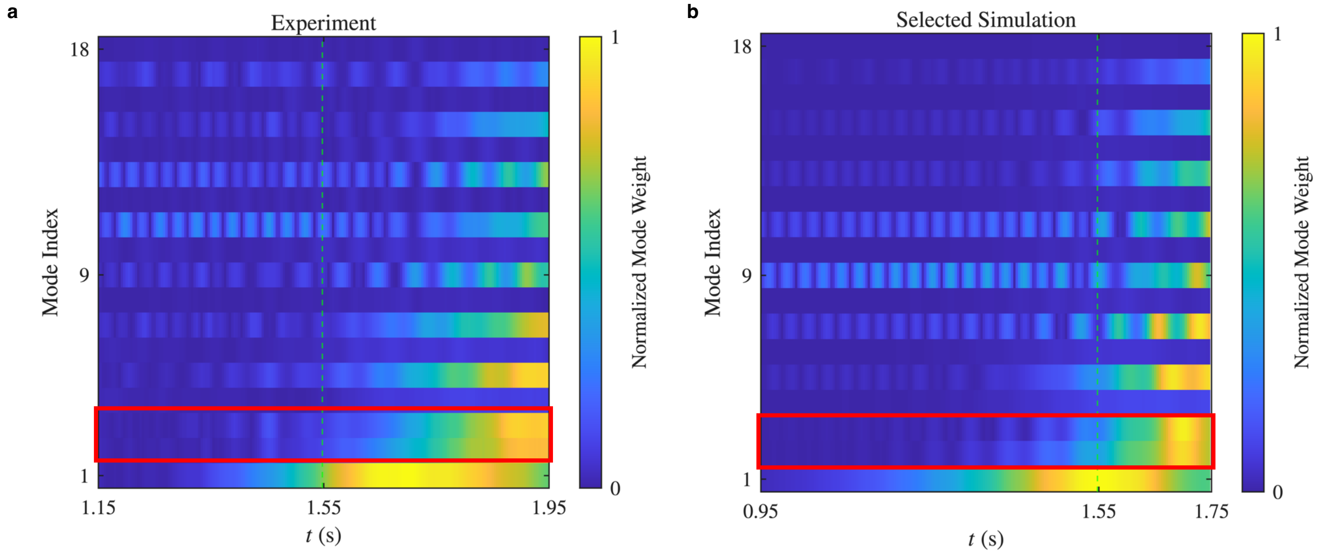

Supplementary Figure 29. Mode decomposition results for short-time acoustic-wave–kink interaction in the kink control case shown in Fig. 5**g** and **h**. Mode frequencies increase with mode index. Green dashed lines mark the predicted arrival time of the wave packet center at the kink center position and red boxes indicate the internal modes.

Supplementary Note 22. ADDITIONAL EXPERIMENTAL RESULTS AND COMPARISON WITH SIMULATIONS

22.1 Kink control with acoustic wave packets: Tuning wave packet amplitude

To demonstrate kink control by varying wave packet amplitude, Supplementary Figs. 30, 32, 34, and 36 show, for different excitation amplitudes, our experimental KL chain's measured spatiotemporal response in terms of angular position, normalized angular velocity, spring strain, and total energy per rotor, respectively. These spatiotemporal responses correspond to the cases shown in Fig. 5**i**. A few simulated example responses with different excitation amplitudes are provided in Supplementary Figs. 31, 33, 35, and 37 for comparison (corresponding to Fig. 5**j**). As can be seen Fig. 5**i** and **j**, in both experiment and simulation, we see that, in general, stronger excitations increase both the distance traveled and the velocity of the kink.

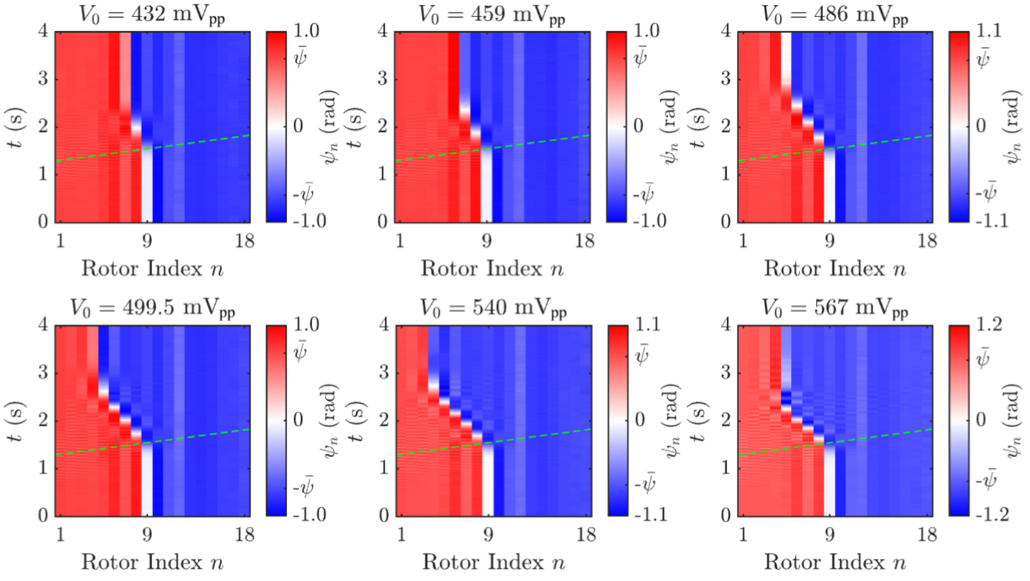

Supplementary Figure 30. Experimentally measured spatiotemporal chain responses (corresponding to Fig. 5**i**) in terms of angular position for kink control cases at various excitation amplitudes, with green dashed line denoting the predicted wave packet center position based on the group velocity at the excitation frequency in the infinite homogeneous chain.

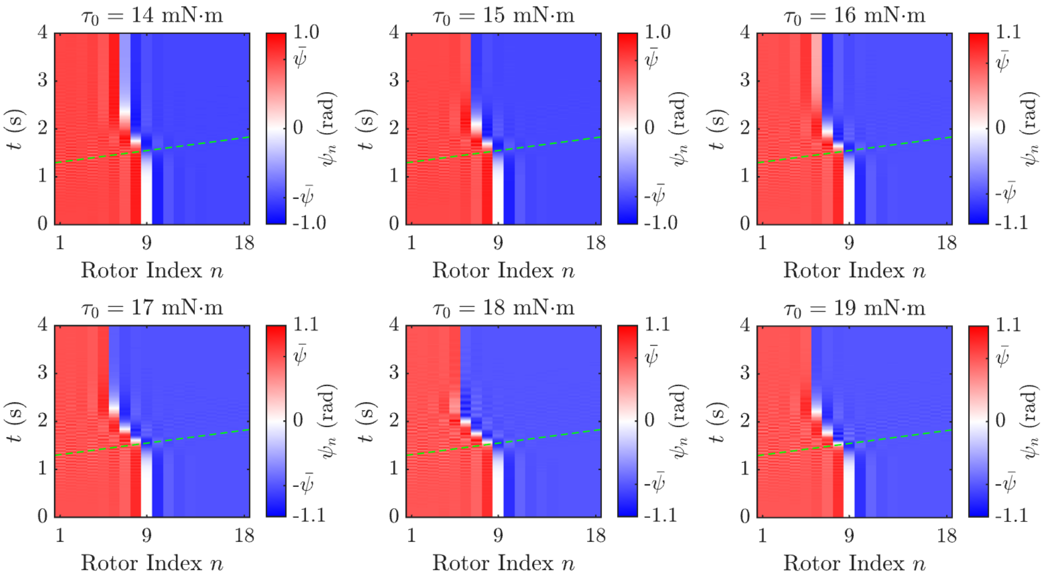

Supplementary Figure 31. Six simulated spatiotemporal chain responses (at 1 mN m intervals selected from Fig. 6**j**) in terms of angular position for kink control cases at various excitation amplitudes, with green dashed line denoting the predicted wave packet center position based on the group velocity at the excitation frequency in the infinite homogeneous chain.

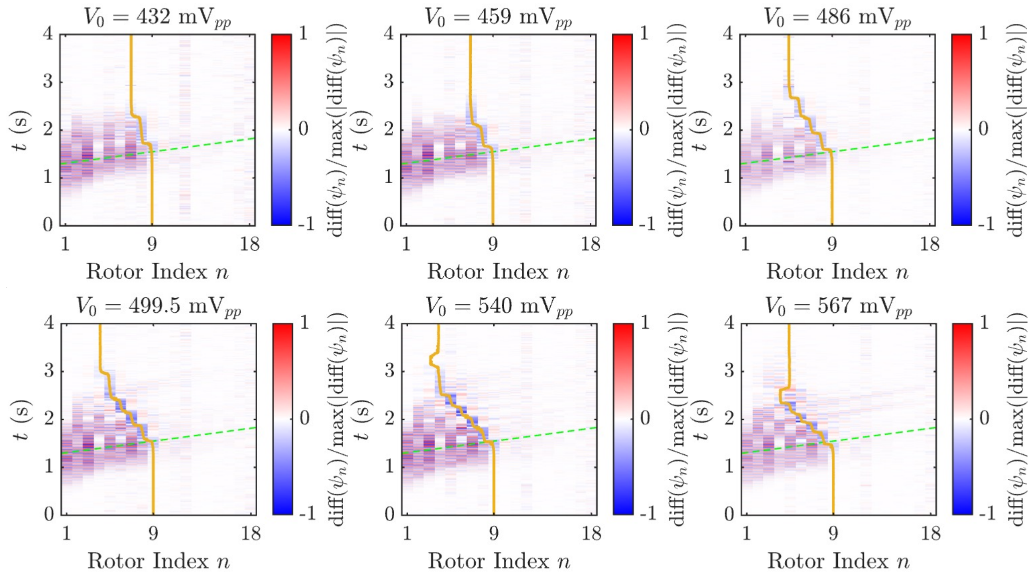

Supplementary Figure 32. Experimentally measured spatiotemporal chain responses (corresponding to Fig. 5**i**) in terms of normalized angular velocity for kink control cases at various excitation amplitudes, with green dashed line denoting the predicted wave packet center position based on the group velocity at the excitation frequency in the infinite homogeneous chain and yellow solid line indicating the fitted position of the kink center.

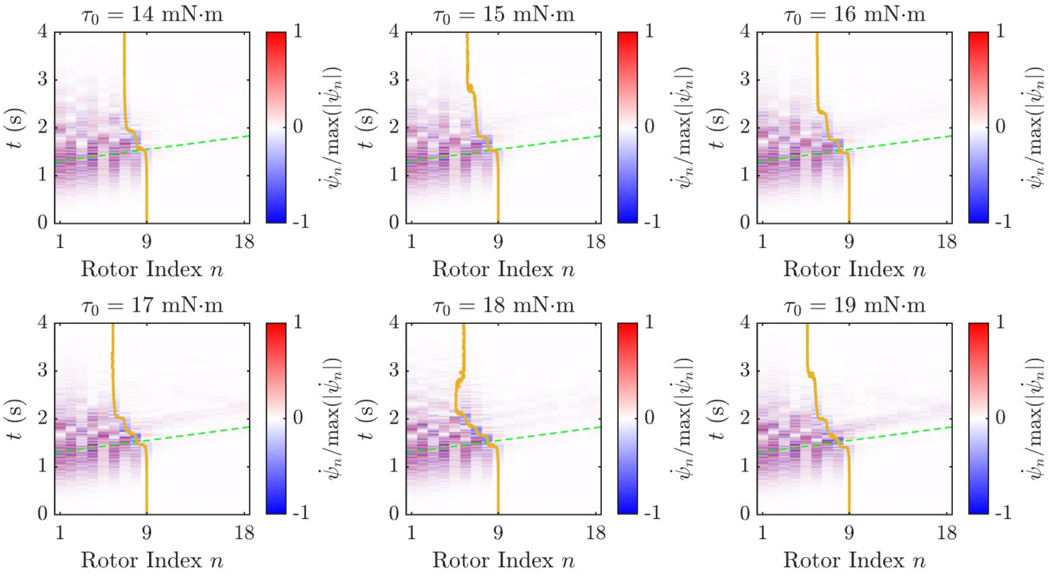

Supplementary Figure 33. Six simulated spatiotemporal chain responses (at 1 mN m intervals selected from Fig. 6**j**) in terms of normalized angular velocity for kink control cases at various excitation amplitudes, with green dashed line denoting the predicted wave packet center position based on the group velocity at the excitation frequency in the infinite homogeneous chain and yellow solid line indicating the fitted position of the kink center.

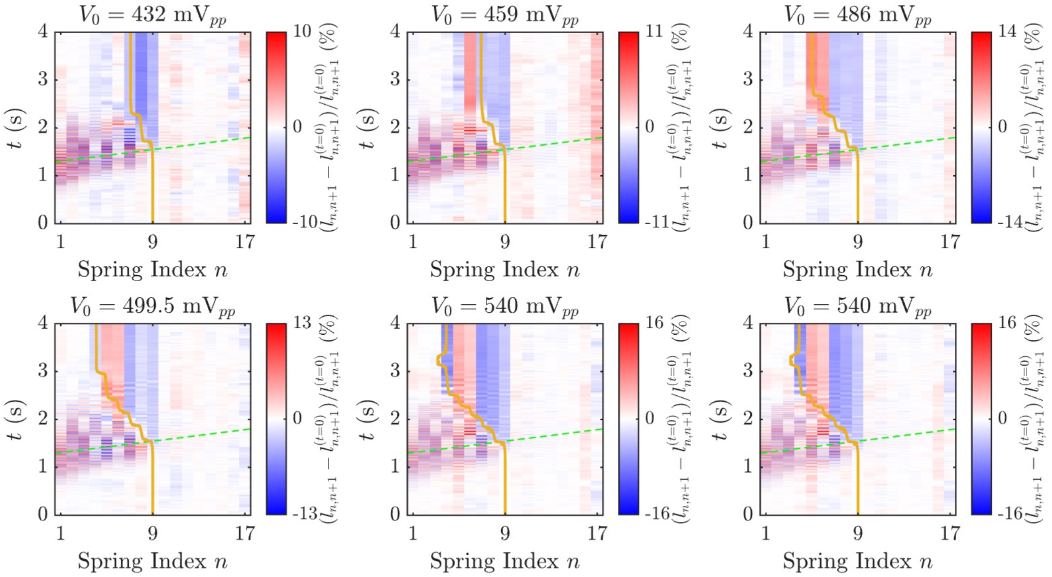

Supplementary Figure 34. Experimentally measured spatiotemporal chain responses (corresponding to Fig. 5**i**) in terms of spring strain for kink control cases at various excitation amplitudes, with green dashed line denoting the predicted wave packet center position based on the group velocity at the excitation frequency in the infinite homogeneous chain and yellow solid line indicating the fitted position of the kink center. $l^{(t=0)}_{n,n+1}$ denotes the length of the spring connecting rotors n and $n+1$ at $t = 0$, calculated from the tracked rotor angles in the recorded frame.

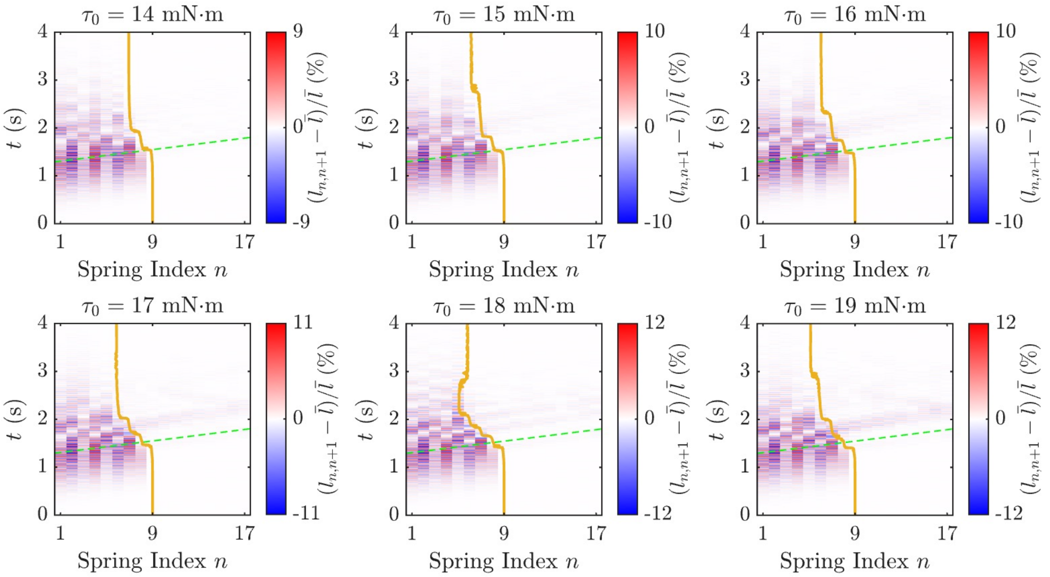

Supplementary Figure 35. Six simulated spatiotemporal chain responses (at 1 mN m intervals selected from Fig. 6**j**) in terms of spring strain for kink control cases at various excitation amplitudes, with green dashed line denoting the predicted wave packet center position based on the group velocity at the excitation frequency in the infinite homogeneous chain and yellow solid line indicating the fitted position of the kink center.

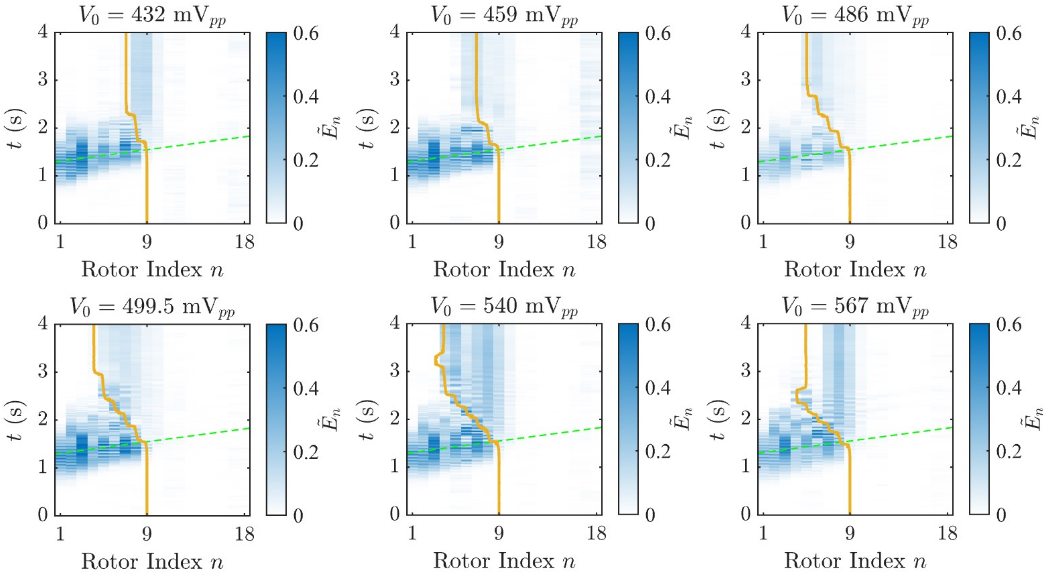

Supplementary Figure 36. Experimentally measured spatiotemporal chain responses (corresponding to Fig. 5**i**) in terms of normalized total energy at each site for kink control cases at various excitation amplitudes, with green dashed line denoting the predicted wave packet center position based on the group velocity at the excitation frequency in the infinite homogeneous chain and yellow solid line indicating the fitted position of the kink center. Color bar is truncated for visualization purposes.

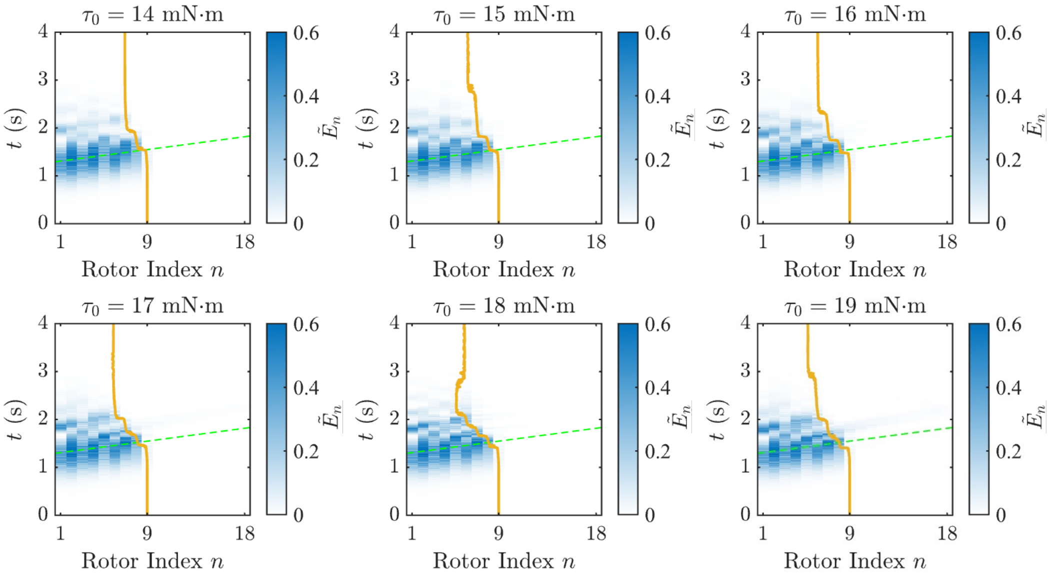

Supplementary Figure 37. Six simulated spatiotemporal chain responses (at 1 mN m intervals selected from Fig. 6**j**) in terms of normalized total energy at each site for kink control cases at various excitation amplitudes, with green dashed line denoting the predicted wave packet center position based on the group velocity at the excitation frequency in the infinite homogeneous chain and yellow solid line indicating the fitted position of the kink center. Color bar is truncated for visualization purposes.

22.2 Kink generation with acoustic wave packets

Supplementary Figs. 38 and 39 show the experimental and simulated spatiotemporal chain responses in terms of angular position, angular velocity, and spring strain, respectively, for the kink generation case as shown in Fig. 6.

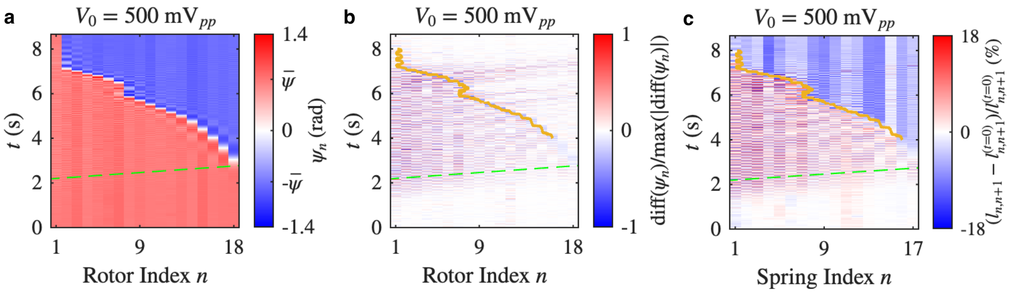

Supplementary Figure 38. Experimentally measured spatiotemporal chain responses in terms of **a** angular position, **b** normalized angular velocity, and **c** spring strain for the kink generation case, with green dashed lines denoting the group velocity at the excitation frequency in the infinite homogeneous chain (with start time corresponding to the end of the ramp) and yellow solid lines indicating the kink's fitted center position from 4 s to 8 s. $l^{(t=0)}_{n,n+1}$ denotes the length of the spring connecting rotors n and $n+1$ at $t = 0$, calculated from the tracked rotor angles in the recorded frame.

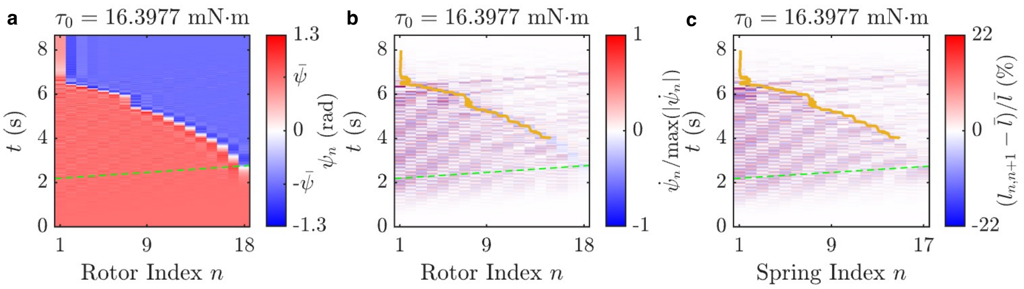

Supplementary Figure 39. Simulated spatiotemporal chain responses in terms of **a** angular position, **b** normalized angular velocity, and **c** spring strain for the kink generation case, with green dashed lines denoting the group velocity at the excitation frequency in the infinite homogeneous chain (with start time corresponding to the end of the ramp) and yellow solid lines indicating the kink's fitted center position from 4 s to 8 s.

Supplementary Note 23. ADDITIONAL SIMULATIONS OF KINK CONTROL IN THE KL CHAIN WITH EXPERIMENTAL PARAMETERS AND ONSITE DAMPING

23.1 Simulations of varying excitation amplitude

We performed numerical studies on the experimental ($N = 18$) and extended ($N = 280$) KL chains, both incorporating onsite damping (c_{exp}), to examine the effect of varying excitation amplitude. The kink was placed at the chain center ($\psi_{N/2} = 0$) and the first rotor was driven according to Supplementary Eq. 97.

Supplementary Fig. 40 presents the simulated spatiotemporal response of the 18-rotor chain under varying driving torque amplitudes, including additional cases with finer amplitude steps near that shown in Fig. 5**h**. Together with Supplementary Fig. 37, the results show that increasing the excitation amplitude generally enhances the kink's propagation distance, velocity, and associated energy radiation. Beyond a certain threshold (*e.g.*, the last panel in Supplementary Fig. 40), however, the propagation distance decreases, and the kink motion can become less uniform or even reverses direction, as seen for $\tau_0 = 18$ mN m in Supplementary Fig. 37. These counterintuitive behaviors are attributed to boundary effects, where the kink interacts with waves reflected from the chain ends.

To minimize boundary effects, we simulated a 280-rotor KL chain using the same unit-cell parameters and damping values as before. As shown in Supplementary Fig. 41, the kink propagates a shorter distance at the same excitation amplitude compared with the 18-rotor chain, confirming that boundary reflections contribute to the extended propagation observed previously. Similar to the shorter chain, increasing the excitation amplitude enhances kink motion and velocity up to a threshold, which shifts to a higher amplitude in the longer system. At sufficiently high amplitudes, chaotic responses and even backward propagation emerge. Overall, these results demonstrate that kink dynamics can be effectively tuned through the excitation amplitude.

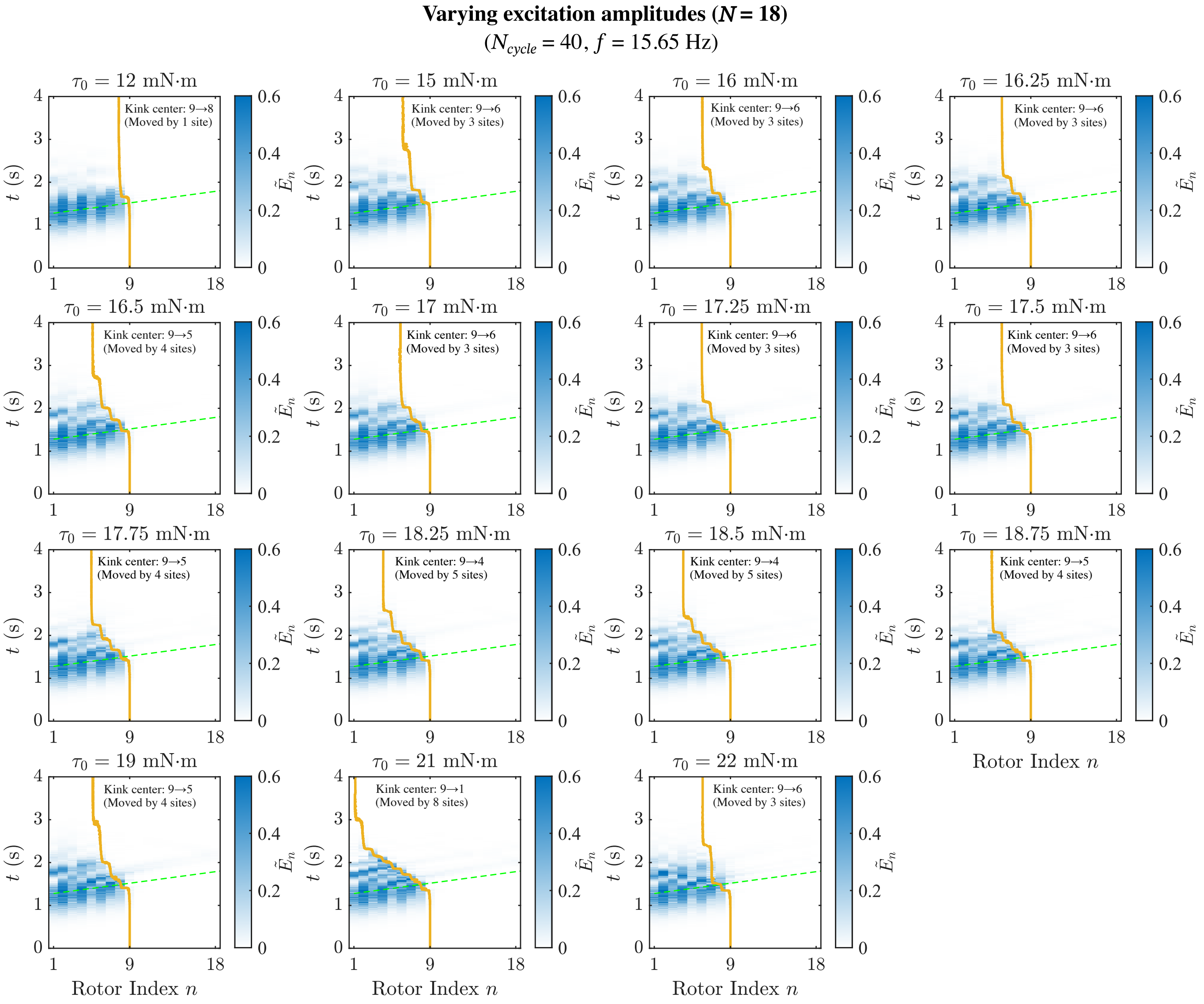

Supplementary Figure 40. Simulated spatiotemporal responses in terms of normalized total energy at each site of the 18-rotor KL chain with onsite damping under varying driving torque amplitudes. Green dashed lines denote the predicted wave packet center position based on the group velocity at the excitation frequency in the infinite homogeneous chain and yellow solid lines indicate the fitted position of the kink center. Color bars are truncated for visualization purposes.

Varying excitation amplitudes ($N = 280$)

($N_{cycle} = 40$, $f = 15.65$ Hz)

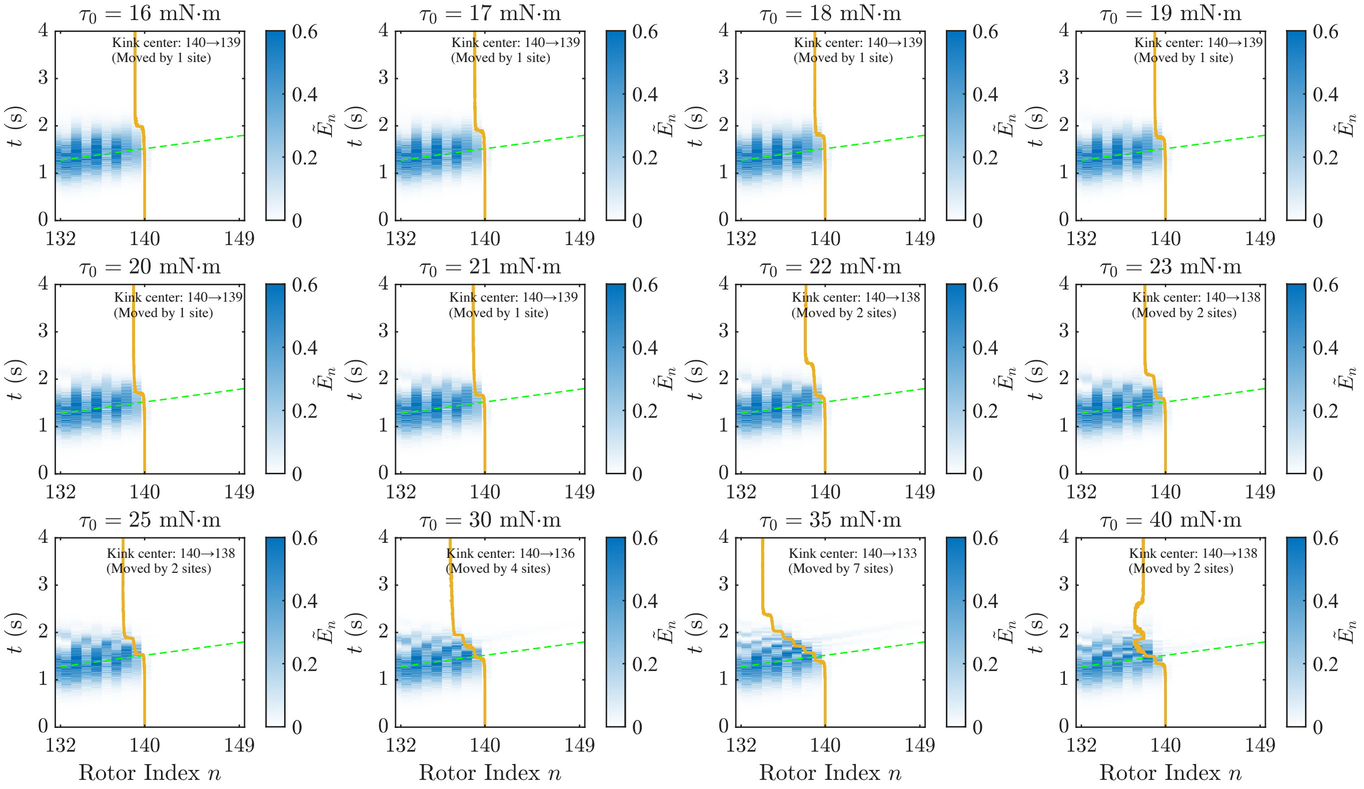

Supplementary Figure 41. Simulated spatiotemporal responses in terms of normalized total energy at each site of the 280-rotor KL chain with onsite damping under varying driving torque amplitudes. Green dashed lines denote the predicted wave packet center position based on the group velocity at the excitation frequency in the infinite homogeneous chain and yellow solid lines indicate the fitted position of the kink center. Color bars are truncated for visualization purposes.

23.2 Simulations of varying excitation frequencies

We examined the effect of varying excitation frequency (within the pass band) in the 18- and 280-rotor KL chains, both with the same onsite damping, excitation setup, and kink placement as before.

Supplementary Fig. 42 shows the simulated spatiotemporal response of the 18-rotor chain under different driving torque frequencies. The results indicate that varying the excitation frequency can cause substantial changes in the kink dynamics and the associated energy radiation. These variations are again attributed to boundary effects arising from the short chain length. Supplementary Fig. 43 shows that once boundary effects become negligible, the kink propagates a shorter distance, consistent with the previous amplitude-dependent cases. Moreover, varying the excitation frequency results in distinct propagation behaviors, confirming that kink motion can be controlled by tuning the excitation frequency.

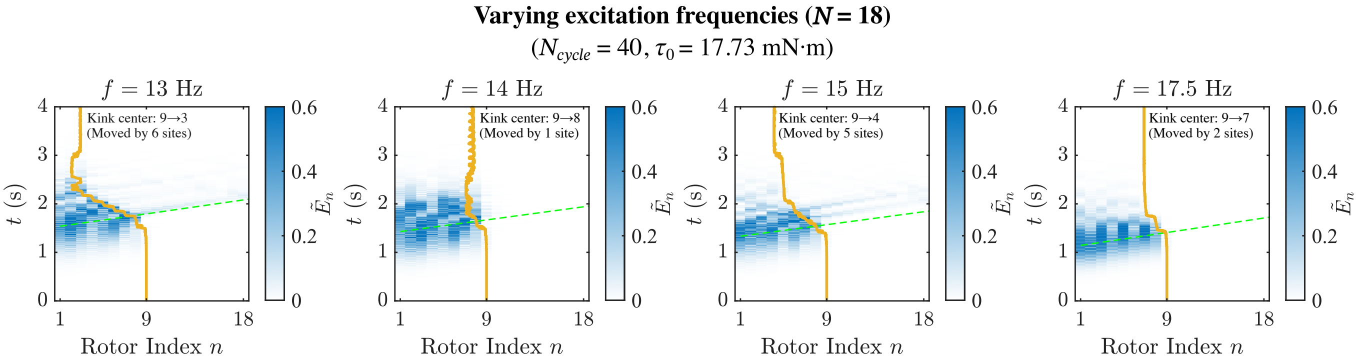

Supplementary Figure 42. Simulated spatiotemporal responses in terms of normalized total energy at each site of the 18-rotor KL chain with onsite damping under varying driving torque frequencies. Green dashed lines denote the predicted wave packet center position based on the group velocity at the excitation frequency in the infinite homogeneous chain and yellow solid lines indicate the fitted position of the kink center. Color bars are truncated for visualization purposes.

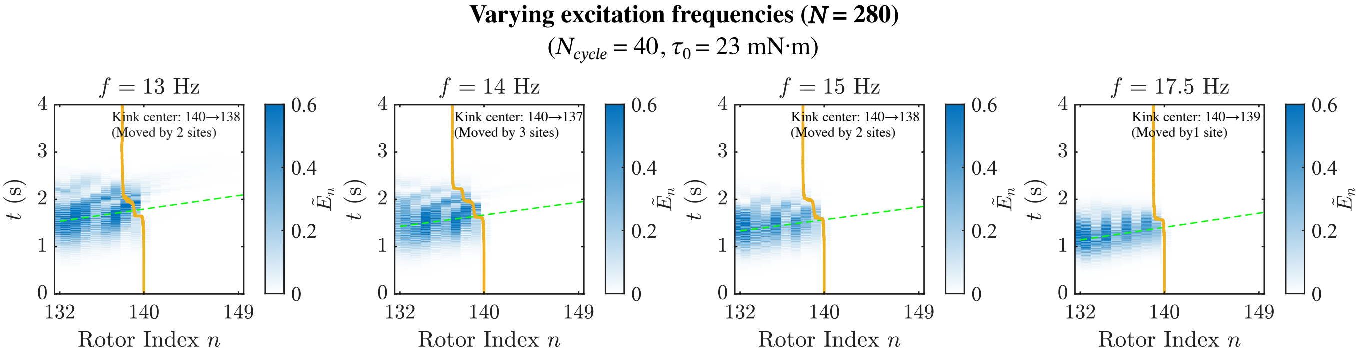

Supplementary Figure 43. Simulated spatiotemporal responses in terms of normalized total energy at each site of the 280-rotor KL chain with onsite damping under varying driving torque frequencies. Green dashed lines denote the predicted wave packet center position based on the group velocity at the excitation frequency in the infinite homogeneous chain and yellow solid lines indicate the fitted position of the kink center. Color bars are truncated for visualization purposes.

23.3 Simulations of varying excitation width

We examined the effect of varying excitation width (*i.e.*, time duration) in the 18- and 280-rotor KL chains, both with the same onsite damping, excitation setup, and kink placement as before.

Supplementary Figs. 44 and 45 show the simulated spatiotemporal response of the 18- and 280-rotor chain under different driving torque widths ($\sigma_t = 0.15 N_{\text{cycle}}/f$; the shifted starting time of the predicted wave packet center position is due to $t_0 = N_{\text{cycle}}/(2f)$), respectively. We observed that increasing the excitation width generally enhances the kink's propagation distance and velocity, as well as the associated energy radiation. These results confirm that kink motion can be effectively controlled by tuning the excitation width.

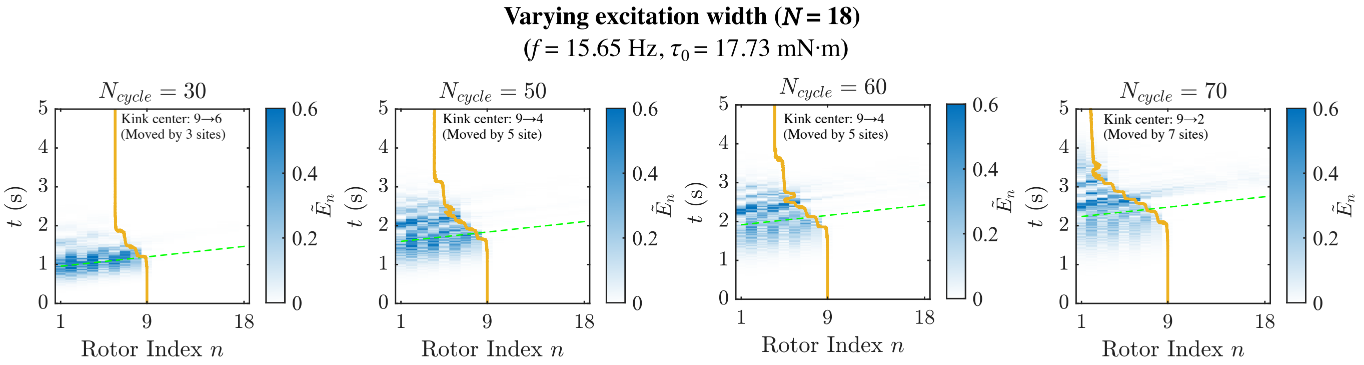

Supplementary Figure 44. Simulated spatiotemporal responses in terms of normalized total energy at each site of the 18-rotor KL chain with onsite damping under varying driving torque widths. Green dashed lines denote the predicted wave packet center position based on the group velocity at the excitation frequency in the infinite homogeneous chain and yellow solid lines indicate the fitted position of the kink center. Color bars are truncated for visualization purposes.

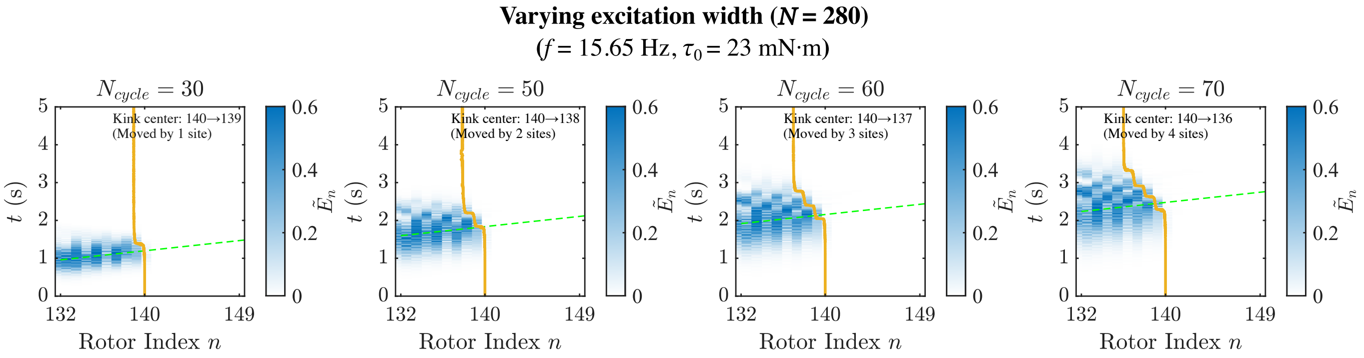

Supplementary Figure 45. Simulated spatiotemporal responses in terms of normalized total energy at each site of the 280-rotor KL chain with onsite damping under varying driving torque widths. Green dashed lines denote the predicted wave packet center position based on the group velocity at the excitation frequency in the infinite homogeneous chain and yellow solid lines indicate the fitted position of the kink center. Color bars are truncated for visualization purposes.

23.4 Simulations of varying initial kink center positions

We examined the effect of varying the initial kink center position in the 18- and 280-rotor KL chains, both with the same onsite damping and excitation setup as before.

In the 18-rotor chain (Supplementary Fig. 46), the kink's mobility decreases as it is positioned farther from the excitation source, which can be attributed to reduced boundary influence and acoustic wave dissipation over longer propagation distances. In the 280-rotor chain (Supplementary Fig. 47), where boundary effects are negligible, the kink's mobility still decreases with distance from the excitation source, isolating the role of damping in the system. Furthermore, Supplementary Fig. 48 shows that when boundary effects are absent and acoustic wave dissipation remains comparable (*i.e.*, the kink is placed the same distance from the excitation source), the kink and acoustic wave dynamics change only minimally. Overall, these results confirm that tuning the initial kink position influences propagation only when boundary effects are present.

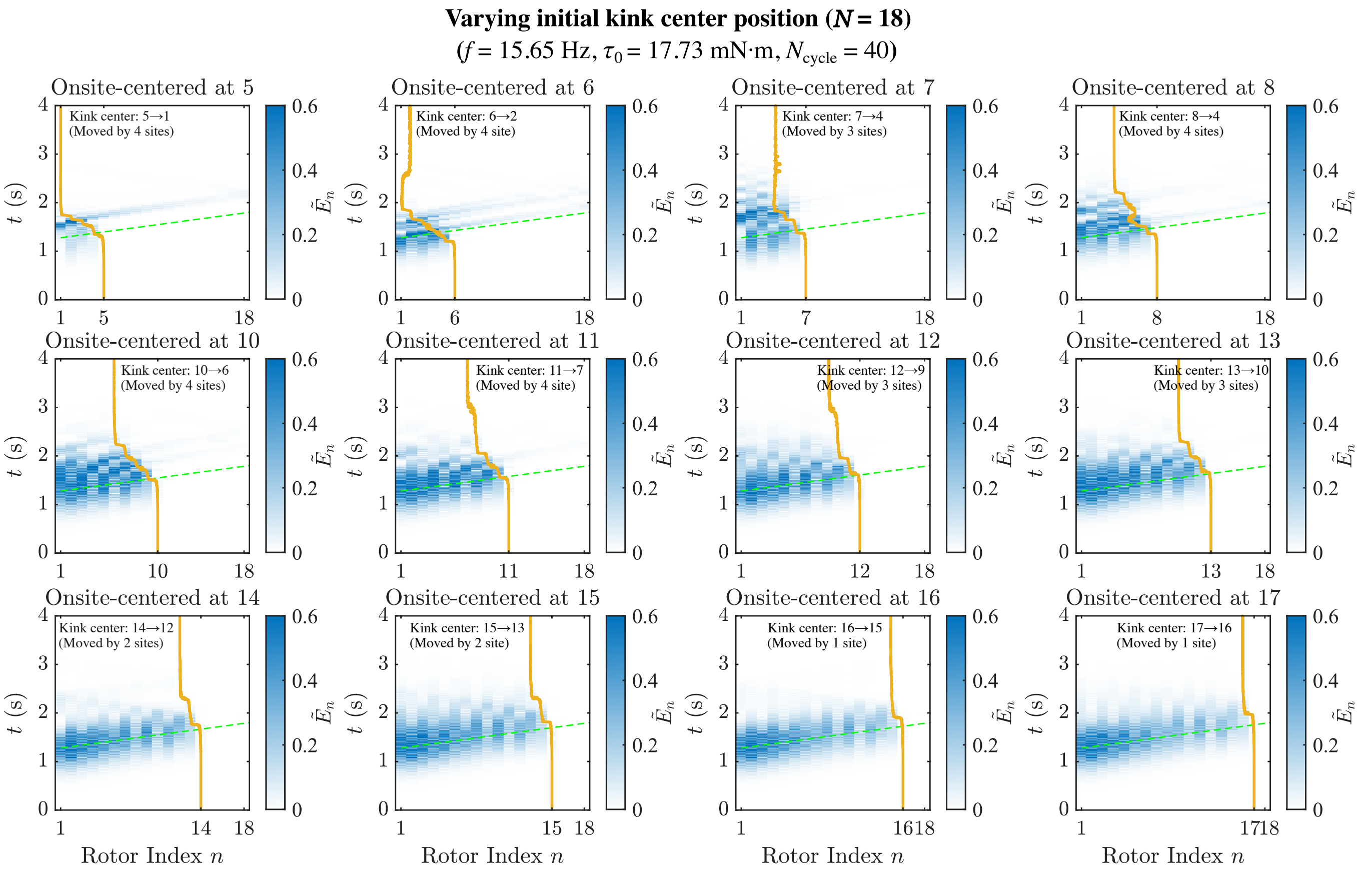

Supplementary Figure 46. Simulated spatiotemporal responses in terms of normalized total energy at each site of the 18-rotor KL chain with onsite damping and different initial kink center positions. The excitation is applied at rotor 1. Green dashed lines denote the predicted wave packet center position based on the group velocity at the excitation frequency in the infinite homogeneous chain and yellow solid lines indicate the fitted position of the kink center. Color bars are truncated for visualization purposes.

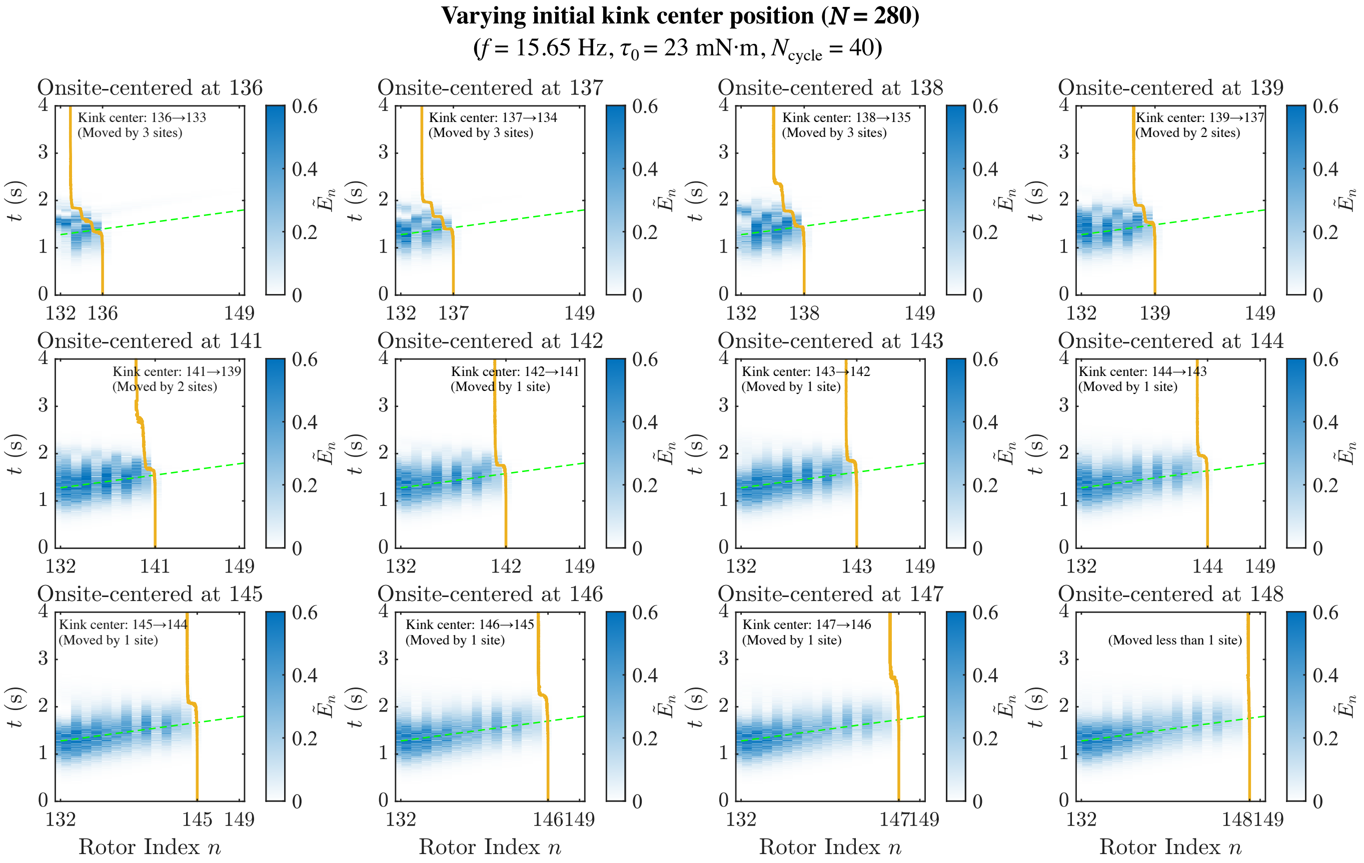

Supplementary Figure 47. Simulated spatiotemporal responses in terms of normalized total energy at each site of the 280-rotor KL chain with onsite damping and different initial kink center positions. The excitation is applied at rotor 132. Green dashed lines denote the predicted wave packet center position based on the group velocity at the excitation frequency in the infinite homogeneous chain and yellow solid lines indicate the fitted position of the kink center. Color bars are truncated for visualization purposes.

Varying initial kink center position ($N = 280$)

($f = 15.65$ Hz, $\tau_0 = 23$ mN·m, $N_{cycle} = 40$)

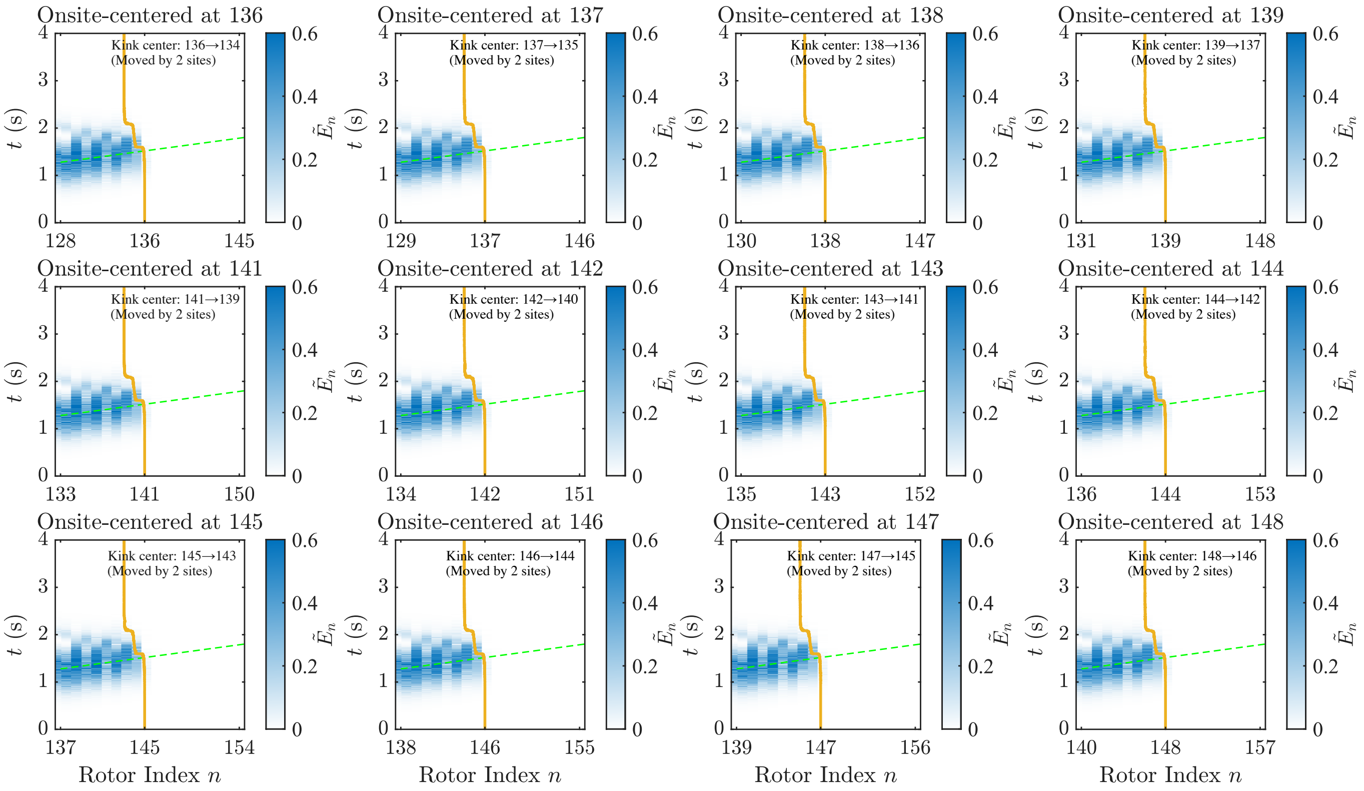

Supplementary Figure 48. Simulated spatiotemporal responses in terms of normalized total energy at each site of the 280-rotor KL chain with onsite damping and different initial kink center positions. The excitation is applied eight rotors to the left of the kink center. Green dashed lines denote the predicted wave packet center position based on the group velocity at the excitation frequency in the infinite homogeneous chain and yellow solid lines indicate the fitted position of the kink center. Color bars are truncated for visualization purposes.

23.5 Simulations of varying onsite damping

We examined the effect of varying the onsite damping in the 18- and 280-rotor KL chains, both with the same excitation setup and kink placement as before.

In the 18-rotor chain (Supplementary Fig. 49), reducing damping can either enhance or weaken kink mobility, a counterintuitive behavior that we again attribute to boundary effects arising from the short chain length. In the 280-rotor chain (Supplementary Fig. 50), where boundary effects are negligible, reducing damping enhances kink mobility. This demonstrates that kink propagation can be effectively controlled by tuning the intrinsic damping of the chain.

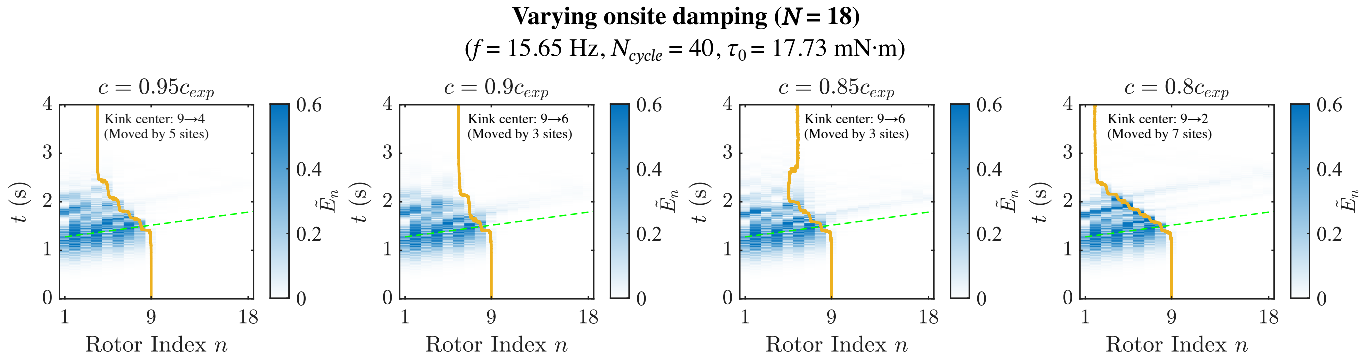

Supplementary Figure 49. Simulated spatiotemporal responses in terms of normalized total energy at each site of the 18-rotor KL chain with different onsite damping values. Green dashed lines denote the predicted wave packet center position based on the group velocity at the excitation frequency in the infinite homogeneous chain and yellow solid lines indicate the fitted position of the kink center. Color bars are truncated for visualization purposes.

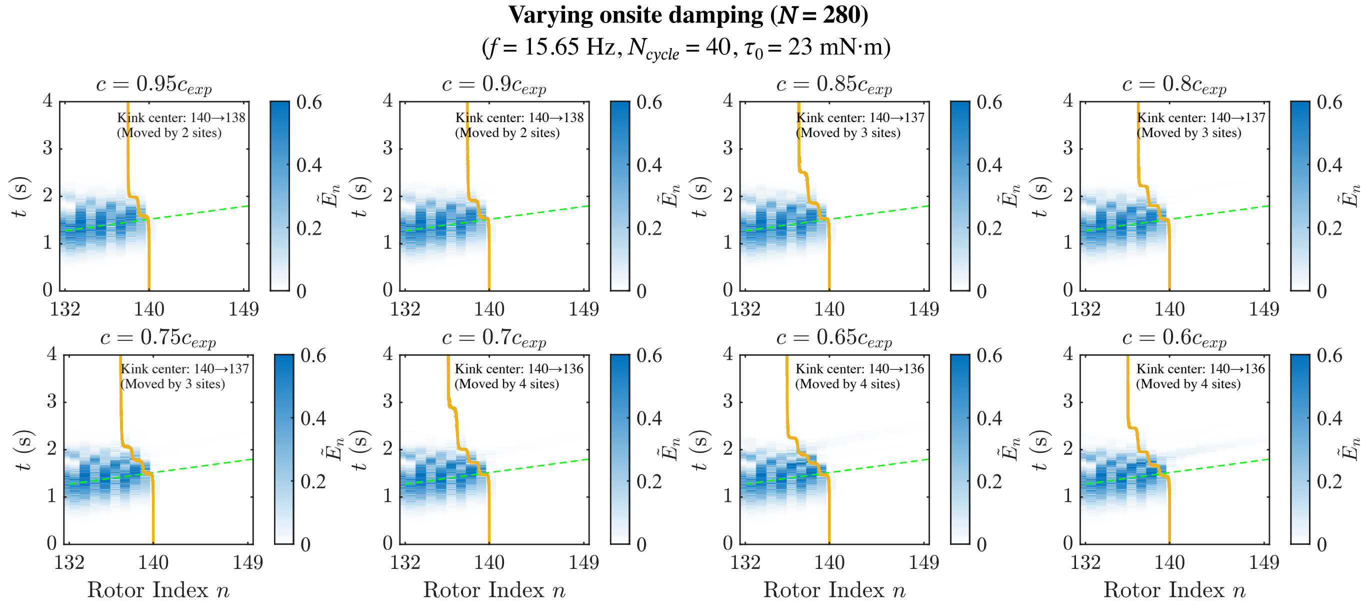

Supplementary Figure 50. Simulated spatiotemporal responses in terms of normalized total energy at each site of the 280-rotor KL chain with different onsite damping values. Green dashed lines denote the predicted wave packet center position based on the group velocity at the excitation frequency in the infinite homogeneous chain and yellow solid lines indicate the fitted position of the kink center. Color bars are truncated for visualization purposes.

23.6 Simulations of varying chain lengths

We examined the effect of varying the chain length of the KL chain. The excitation was applied eight rotors to the left of the kink center. Supplementary Fig. 51 shows that increasing the chain length reduces boundary effects, as evidenced by the decrease in kink mobility.

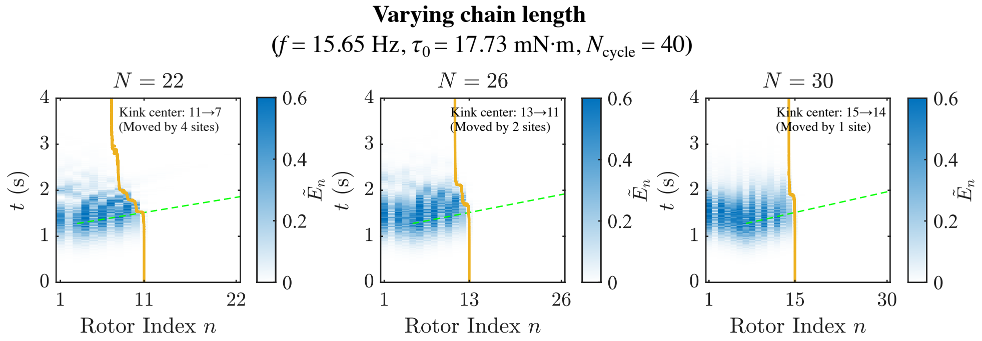

Supplementary Figure 51. Simulated spatiotemporal responses in terms of normalized total energy at each site of the KL chain with onsite damping and different chain lengths. The excitation is applied eight rotors to the left of the kink center. Green dashed lines denote the predicted wave packet center position based on the group velocity at the excitation frequency in the infinite homogeneous chain and yellow solid lines indicate the fitted position of the kink center. Color bars are truncated for visualization purposes.

Supplementary Note 24. ADDITIONAL SIMULATIONS OF KINK GENERATION VIA ACOUSTIC WAVES IN THE KL CHAIN WITH EXPERIMENTAL PARAMETERS AND ONSITE DAMPING

We performed numerical studies on the experimental ($N = 18$) and extended ($N = 280$) KL chains, both in the right-polarized state (RPS) and incorporating onsite damping, to examine the effect of varying excitation amplitude. The first rotor was driven according to Supplementary Eq. 99.

Supplementary Fig. 52 presents additional simulations for the 18-rotor RPS chain with finer amplitude steps around the representative case shown in Fig. 6. Nonuniform kink motion is observed in all these cases.

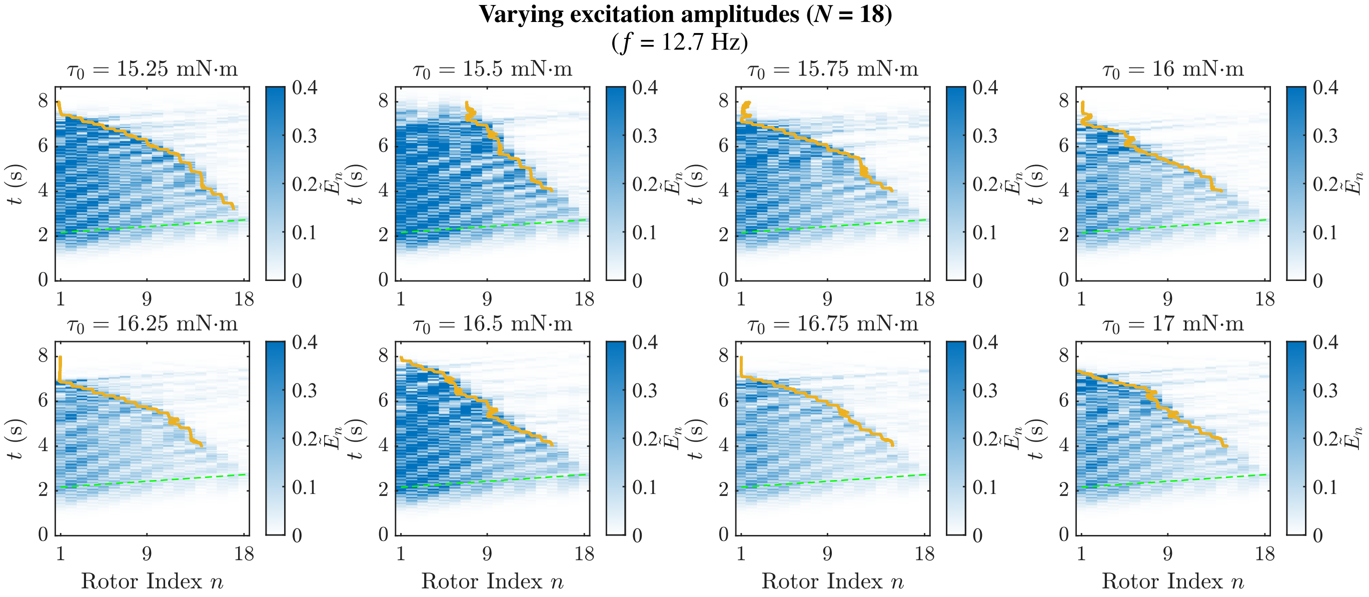

Supplementary Figure 52. Simulated spatiotemporal responses in terms of normalized total energy at each site of the 18-rotor RPS KL chain with onsite damping under different excitation amplitudes. The excitation is applied at rotor 1. Green dashed lines denote the group velocity at the excitation frequency in the infinite homogeneous chain (with start time corresponding to the end of the ramp) and yellow solid lines indicate the kink's fitted center position from 4 s to 8 s (4 s to 7.5 s for the last panel). Color bars are truncated for visualization purposes.

In the 280-rotor chain (Supplementary Fig. 53), the kink propagates a shorter distance at the same excitation amplitude, confirming that boundary effects contribute to the enhanced mobility observed in the shorter chain. At higher amplitudes (last row), the kink travels farther but exhibits increasingly nonuniform motion, suggesting that this behavior arises not only from boundary effects but also from strong excitation, close proximity, and prolonged acoustic-wave–kink interactions, with minor influence from reflections at the right boundary. Overall, these results demonstrate that kink generation and subsequent motion can be effectively tuned by adjusting the excitation amplitude.

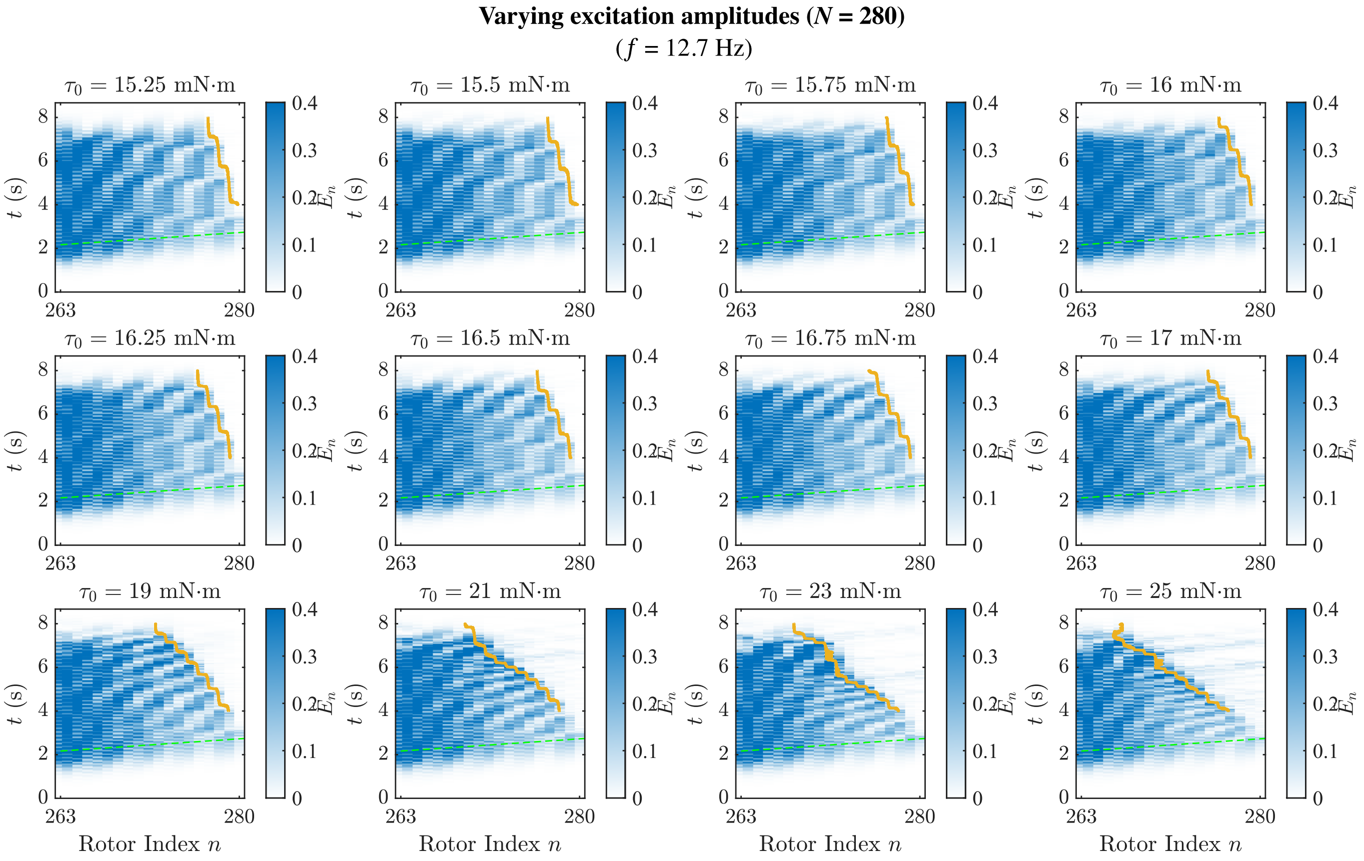

Supplementary Figure 53. Simulated spatiotemporal responses in terms of normalized total energy at each site of the 280-rotor RPS KL chain with onsite damping under different excitation amplitudes. The excitation is applied at rotor 263. Green dashed lines denote the group velocity at the excitation frequency in the infinite homogeneous chain (with start time corresponding to the end of the ramp) and yellow solid lines indicate the kink's fitted center position from 4 s to 8 s. Color bars are truncated for visualization purposes.

Supplementary Note 25. SIMULATION OF A REPULSION CASE OF A WF-PHASE KINK USING EXPERIMENTAL PARAMETERS

Here, we provide numerical simulations that demonstrate a repulsion case of the WF-phase kink for parameters corresponding to the experiment, both with and without onsite damping.

We adapt the experimental geometric parameters of the attraction case with $r = 0.8a = 16$ mm and $\bar{l} = 24$ mm, resulting in $d = 1.4548$ and the corresponding zero-energy kink state in the WF phase. The configuration of a rotor chain of $N = 18$ rotors, with the kink centered at the 9th rotor ($\psi_9 = 0$), is shown in Supplementary Fig. 54**a**. We use the same dynamical parameters as those in the experiment. A Gaussian-modulated sinusoidal wave packet is introduced from the 1th rotor as a driving torque, described by $\tau(t) = \tau_0 e^{-(t-t_0)^2/(2\sigma_t^2)} \sin 2\pi f t$, where $\tau_0 = 0.5$ mN m, $t_0 = N_{\text{cycle}}/(2f)$, $\sigma_t = 0.15 N_{\text{cycle}}/f$, $N_{\text{cycle}} = 40$, and $f = 7.98$ Hz (the median of the eigenfrequencies calculated from the chain configuration shown in Supplementary Fig. 54**a**). Supplementary Fig. 54**b** shows the simulated spatiotemporal response without onsite damping. In this case, we observe that the kink can move away from the excitation source by the acoustic waves. However, as including experimentally characterized onsite damping (c_{exp}), we observe that the chain enters a disordered state at larger amplitude ($\tau_0 = 11$ mN m) before the acoustic waves reach the kink, as shown in Supplementary Fig. 55.

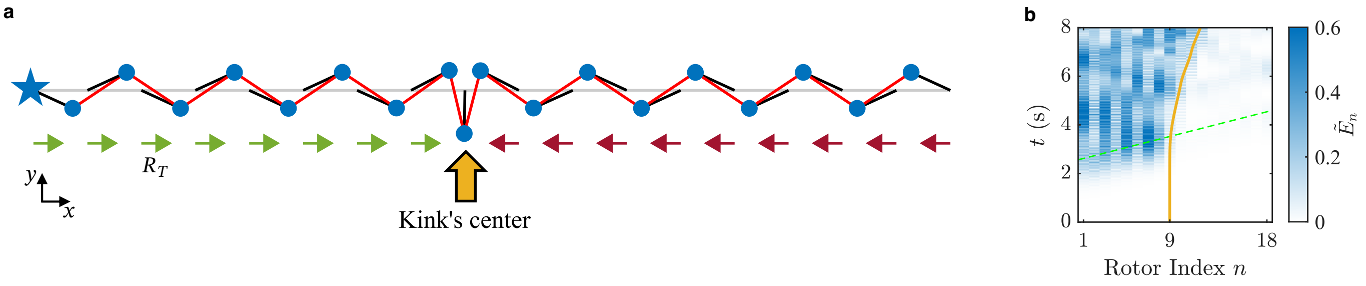

Supplementary Figure 54. Simulation of a repulsion case of a WF-phase kink in the KL chain, consisting of 18 rotors, using experimental parameters and without onsite damping. **a** Configuration of an 18-rotor KL chain with the zero-energy kink positioned at the center of the chain. The blue star indicates the excited rotor. **b** Simulated spatiotemporal response in terms of normalized total energy at each site. Color bar is truncated for visualization purposes. Green dashed line denotes the predicted wave packet center position based on the group velocity at the excitation frequency in the infinite homogeneous chain and the yellow solid line indicates the fitted position of the kink center.

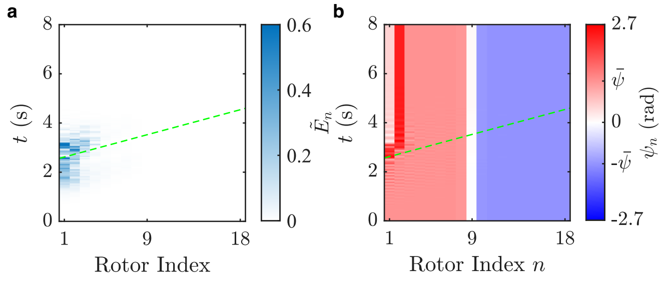

Supplementary Figure 55. Simulation of a repulsion case of a WF-phase kink in the KL chain, consisting of 18 rotors, using experimental parameters and experimentally characterized onsite damping. **a**, **b** Simulated spatiotemporal responses in terms of normalized total energy at each site (**a**) and angular position (**b**). Green dashed lines denote the predicted wave packet center position based on the group velocity at the excitation frequency in the infinite homogeneous chain.

Supplementary Note 26. EXAMPLES OF ENERGY TRANSMISSION FROM AN INPUT ACOUSTIC WAVE PACKET TO A MOVING KINK THROUGH ACOUSTIC-WAVE–KINK INTERACTION IN THE KL CHAIN

To illustrate the efficiency of acoustic driving, we present two examples of energy transmission from an input acoustic wave packet to a moving kink through acoustic-wave–kink interaction in the KL chain. We selected the kink state with parameters $\tilde{r} = 0.9$ and $\tilde{\tilde{l}} = 1.35$, which exhibits an attraction-type interaction, as shown in Supplementary Fig. 19. The acoustic-wave–kink interaction was simulated in a 280-rotor chain, where the first rotor was driven at the mid-band frequency with two different driving amplitudes ($\tilde{\tau}_0 = 0.006647$ and 0.02216). The corresponding spatiotemporal responses are shown in Supplementary Fig. 56 The normalized kink velocities (relative to the speed of sound[1]) after the interaction are fitted as -0.0137 and -0.0759, respectively. In Supplementary Fig. 56, the red dash-dotted line indicates the time $t = 3t_0$ (when the entire wave packet has entered the chain) used to quantify the acoustic wave energy, which corresponds to the total chain energy since the kink is initially static and has zero energy before the interaction. The blue dash-dotted line marks the time $t = Na/v_g$, used to evaluate the kink energy. Although the normalized kink width $\tilde{w}_0$ is approximately 1.3, we selected the kink center rotor (around rotor 137 and 120, respectively), together with its three neighboring rotors on each side (seven rotors in total), at $t = Na/v_g$ to compute the total kink energy. By comparing the moving-kink energy with the input acoustic wave packet energy, we find that approximately 2.27% of the acoustic wave packet energy is transferred to the kink in **a** and about 6.35% in **b**, while the remaining energy is transmitted, reflected, or radiated away from the kink during and after the interaction as the kink propagates.

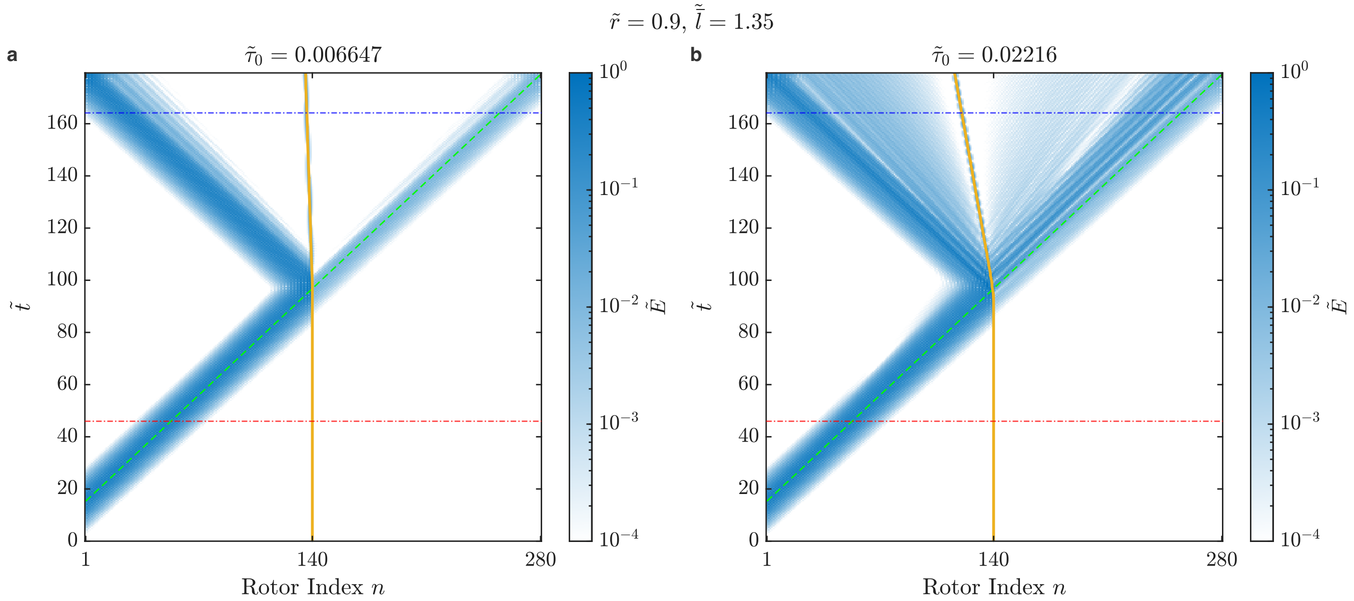

Supplementary Figure 56. Simulated spatiotemporal chain responses in terms of normalized total energy at each site of acoustic-wave–kink interaction for a kink state with parameters $\tilde{r} = 0.9$ and $\tilde{\tilde{l}} = 1.35$ in a 280-rotor KL chain. Green dashed lines denote the predicted wave packet center position based on the group velocity at the excitation frequency in the infinite homogeneous chain and yellow solid lines indicate the fitted position of the kink center. The applied torque is over three times greater in **a** compared to **b**.

Supplementary Note 27. PROSPECT OF EXPERIMENTALLY SCALING KL CHAINS TO USEFUL DIMENSIONS

Because the experimental size of our physical KL chain is strongly constrained by the need for z-stacking to avoid self-contact, we propose a potential solution to reduce the z-stacking requirement, as illustrated in Supplementary Fig. 57. One could imagine the two fixed boundaries in gray are mounted to a substrate in the z-x plane. In addition, scaling of such rotary systems has been demonstrated in, *e.g.*, 3D printed gear-based mechanical metamaterials with gears down to 3.6 mm diameter[15].

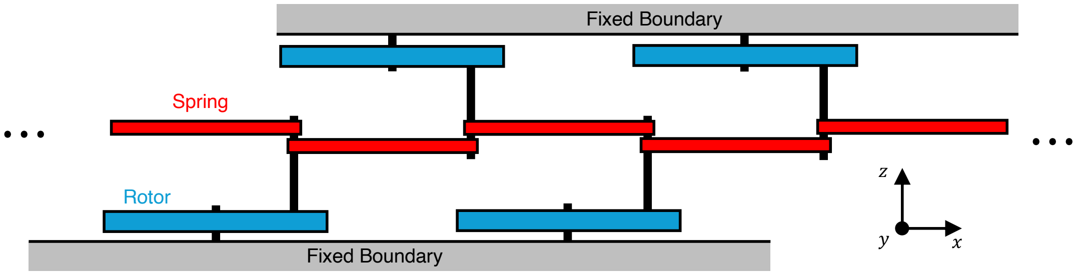

Supplementary Figure 57. Potential solution for reducing the z-stacking in realizing physical KL chains.

Supplementary Note 28. DETAILS ON SUPPLEMENTARY MOVIES

Supplementary Movies 1 and 2 demonstrate the kink in the middle of the chain ($\psi_9 = 0$) shows the attraction type of motion when excited within the pass band ($f = 15.65$ Hz) from either hard edge (bottom or top) of the chain. In both tests, the excitation signal followed Supplementary Eq. 96, $V_0 = 540$ mV$_{pp}$, and the gain of shaker 1 was set to 10 dB.

Supplementary Movies 3 and 4 show that the kink in the middle of the chain ($\psi_9 = 0$) remains stationary when excited outside the pass band ($f = 5$ Hz) from either hard edge (bottom or top) of the chain. In both tests, the excitation signal followed Supplementary Eq. 96, except for f, $V_0 = 540$ mV$_{pp}$, and the gain of shaker 1 was set to 10 dB.

Supplementary Movies 5–7 show the generation and propagation of the kink when excited at frequencies inside ($f = 12.7$ Hz) and outside the pass band ($f = 6.35$ Hz and $f = 25.4$ Hz) from the hard edge of the homogeneous chain, respectively. For all tests, the excitation signal followed Supplementary Eq. 98, except for f, $V_0 = 500$ mV$_{pp}$, and the gain of shaker 1 was set to 10 dB.

We note that the movies serve solely for demonstration and were not used in the data processing. The slight discrepancy in kink traveling distance and velocity observed between the movies and the data shown in Figs. 5 and 6 could result from small variations in the experimental setup, such as leveling or shaker alignment.

Supplementary References

1. Chen, B. G.-g., Upadhyaya, N. & Vitelli, V. Nonlinear conduction via solitons in a topological mechanical insulator. *Proc. Natl. Acad. Sci.* **111**, 13004–13009 (2014).
2. Kane, C. L. & Lubensky, T. C. Topological boundary modes in isostatic lattices. *Nat. Phys.* **10**, 39–45 (2014).
3. Dauxois, T. & Peyrard, M. *Physics of solitons* (Cambridge University Press, 2006).
4. Gilat, A. & Subramaniam, V. Numerical methods for engineers and scientists. *An Introd. with Appl. Using MATLAB,* **20014** (2013).
5. Chirilus-Bruckner, M., Chong, C., Cuevas-Maraver, J. & Kevrekidis, P. Sine-gordon equation: From discrete to continuum. *The sine-Gordon Model. its Appl. From Pendul. Josephson Junctions to Gravity High-Energy Phys.* 31–57 (2014).
6. Kevrekidis, P. & Weinstein, M. Dynamics of lattice kinks. *Phys. D: Nonlinear Phenom.* **142**, 113–152 (2000).
7. Kevrekidis, P. G. & Cuevas-Maraver, J. A dynamical perspective on the φ^4 model. *Past, Present. Futur.* **26** (2019).
8. Hasenfratz, W. & Klein, R. The interaction of a solitary wave solution with phonons in a one-dimensional model for displacive structural phase transitions. *Phys. A: Stat. Mech. its Appl.* **89**, 191–204 (1977).
9. Ishimori, Y. & Munakata, T. Kink dynamics in the discrete sine-gordon system a perturbational approach. *J. Phys. Soc. Jpn.* **51**, 3367–3374 (1982).
10. Peyrard, M. & Kruskal, M. D. Kink dynamics in the highly discrete sine-gordon system. *Phys. D: Nonlinear Phenom.* **14**, 88–102 (1984).
11. Theodorakopoulos, N., Wünderlich, W. & Klein, R. Lattice phonons in the presence of non-linear excitations. *Solid State Commun.* **33**, 213–216 (1980).
12. Saadatmand, D., Marjaneh, A. M., Askari, A. & Weigel, H. Phonons scattering off discrete asymmetric solitons in the absence of a peierls–nabarro potential. *Chaos, Solitons & Fractals* **180**, 114550 (2024).
13. Abdelhady, A. & Weigel, H. Wave-packet scattering off the kink-solution. *Int. J. Mod. Phys. A* **26**, 3625–3640 (2011).
14. Ugural, A. C. & Fenster, S. K. *Advanced mechanics of materials and applied elasticity* (Pearson Education, 2011).
15. Fang, X. *et al.* Programmable gear-based mechanical metamaterials. *Nat. Mater.* **21**, 869–876 (2022).
