# Supplementary material for: Observation of mechanical kink control and generation via acoustic waves
Source: Nat Commun. 2026 Feb 6;17:2428. doi: 10.1038/s41467-026-68688-7 (PMC12988211; doi:10.1038/s41467-026-68688-7)
Supplement: Supplementary file 2 — Description of Additional Supplementary Files [file 41467_2026_68688_MOESM2_ESM.pdf]

Description of Additional Supplementary Files

Supplementary Movie 1: Demonstration of acoustic-wave–kink interaction in the experimental Kane– Lubensky chain when excited within the pass band from the bottom edge of the chain.

Supplementary Movie 2: Demonstration of acoustic-wave–kink interaction in the experimental Kane– Lubensky chain when excited within the pass band from the top edge of the chain.

Supplementary Movie 3: Demonstration of no acoustic-wave–kink interaction in the experimental Kane– Lubensky chain when excited outside (below) the pass band from the bottom edge of the chain.

Supplementary Movie 4: Demonstration of no acoustic-wave–kink interaction in the experimental Kane– Lubensky chain when excited outside (below) the pass band from the top edge of the chain.

Supplementary Movie 5: Demonstration of kink generation and propagation via acoustic waves in the experimental Kane–Lubensky chain when excited within the pass band.

Supplementary Movie 6: Demonstration of kink generation and propagation via acoustic waves in the experimental Kane–Lubensky chain when excited below the pass band.

Supplementary Movie 7: Demonstration of kink generation and propagation via acoustic waves in the experimental Kane–Lubensky chain when excited above the pass band.
